# Mechanochemical *N*-alkylation of imides

**DOI:** 10.3762/bjoc.13.169

**Published:** 2017-08-22

**Authors:** Anamarija Briš, Mateja Đud, Davor Margetić

**Affiliations:** 1 Laboratory for Physical-organic Chemistry, Division of Organic Chemistry and Biochemistry, Ruđer Bošković Institute, Bijenička c. 54, 10000 Zagreb, Croatia

**Keywords:** ball milling, Gabriel reaction, imides, mechanochemistry, *N*-alkylation

## Abstract

The mechanochemical *N*-alkylation of imide derivatives was studied. Reactions under solvent-free conditions in a ball mill gave good yields and could be put in place of the classical solution conditions. The method is general and can be applied to various imides and alkyl halides. Phthalimides prepared under ball milling conditions were used in a mechanochemical Gabriel synthesis of amines by their reaction with 1,2-diaminoethane.

## Introduction

The development of environmentally friendly organic reactions is a growing area of interest [1]. The reduction of the impact of chemical reactions on the environment could be achieved by the minimization of waste produced in the process, the employment of the more efficient reagents and catalysts and by the application of microwave [2], photochemical [3] or high pressure conditions [4], thus reducing reaction time and energy consumption. In recent time, important progress was made in the development of various solvent-free organic reactions [5], especially by the use of the ball milling technique [6–8]. In continuation of our interest in eco-friendly organic syntheses [9–14], we studied mechanochemical *N*-alkylation reactions of imides with alkyl halogenides, and the results are presented in this paper. Until now, ball milling *N*-alkylations of ureas [15], hydrazones [16], imines [17–18], pyridines [19], pyrimidines [20], imidazoles [21], secondary amines [22], as well as allylic alkylation reactions [23] were reported in the literature. The aim of this study was to establish simple and effective imide alkylation mechanochemical protocols. Imides are usually alkylated with alkyl halides in solution (DMF, acetone, DMSO) and the reactions were heated for several hours in the presence of a base [24].

## Results and Discussion

**Scheme 1:**
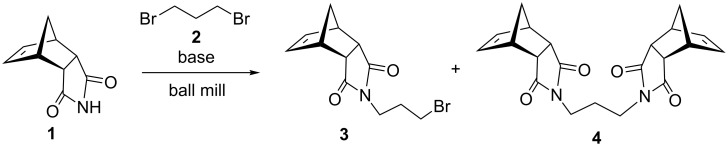
*N*-Alkylation of imide **1** with 1,3-dibromopropane (**2**) in a ball mill.

The reaction of the norbornene *endo*-succinimide **1** [25] with 1,3-dibromopropane (**2**) was used as a model system for the optimization of the reaction conditions [26]. Here, imide **1** is a solid, while dibromopropane is a liquid reagent. It was found that during the ball-milling process (Retsch MM400 mill at 30 Hz, stainless steel 10 mL vial, one 12 mm steel ball) of this solid/liquid system, mono-alkylation and formation of imide **3** was accompanied by the bisalkylated product **4** (Scheme 1).

To optimize the reaction conditions, the molar ratio of reagents, the reaction time and bases were varied. The addition of a small amount of solvent for LAG (liquid-assisted grinding) [27] was tested as well. The results are collected in Table 1. The best results were achieved by the use of K_2_CO_3_ as base, with large excess of dibromide and carbonate. Within one hour of milling, **1** was quantitatively converted to the mono-alkylated product **3** which was and isolated in 88% yield by simple work-up consisting of dissolving the reaction mixture in dichloromethane and washing with water (Table 1, entry 8). Under these milling conditions, an excess of inorganic base may have helped by acting as a grinding auxiliary. A comparison with the synthesis carried out in solution (acetone, 60 °C) showed a significant reduction in time (Table 1, entry 11). Also less efficient was the use of acetone under LAG conditions (Table 1, entry 9). It was found that the outcome of the reaction could be efficiently controlled by variation of molar ratios of reagents. When 0.3 equivalents of dibromide **2** were used, bisalkylation was the sole reaction and imide **4** was isolated in 52% yield (Table 1, entry 5). Other inorganic and organic bases employed were less reactive than K_2_CO_3_, whereas Cs_2_CO_3_ showed a higher reactivity, which, due to the inevitable formation of **4**, prevented clean mono-alkylation.

**Table 1:** *N*-Alkylation of imide **1** with 2.^a^

Entry	Base	Ratio **1**:**2**:base	Time [h]	Ratio **1**:**3**:**4**	Yield [%]^b^

1	K_2_CO_3_	1:1:5	0.5	68:28:4	
2		1:1:5	1	0:82:18	
3		1:1:5	2	0:80:20	
4		1:0.5:5	2	0:45:55	
5		1:0.3:5	2	43:0:57	**4**; 52
6		1:3:5	1	0:93:7	
7		1:3:5	1^c^	14:83:3	
8		1:12:5	1	0:100:0	**3**; 88
9		1:3:5	1^d^	16:65:19	
10		1:3:5	24^d^	0:0:100	**4**; >95
11		1:20:5	24^e^	0:100:0	**3**; >95
12		1:2:2	1 + 1^f^		**3**; 54, 4; 6
13	Na_2_CO_3_	1:12:5	2	89:11:0	
14	Cs_2_CO_3_	1:12:5	2	0:97:3	
15	Cs_2_CO_3_	1:12:3	1	0:97:3	
16	NaHCO_3_	1:1:5	1	48:38:12	
17	DBU	1:1:5	1	24:58:18	
18	DMAP	1:1:5	1	35:61:14	

^a^Retsch MM400 ball mill, 10 mL stainless steel vial, 1 × 12 mm stainless steel ball, 30 Hz; ^b^isolated yields, ratio determined by ^1^H NMR spectroscopy; ^c^2 × 6 mm balls; ^d^LAG acetone (η = 0.25 μL mg^−1^); ^e^acetone, 60 °C; ^f^milling of **1** with K_2_CO_3_ for 1 h, followed by the addition of **2** and LAG DMF (η = 2 μL mg^−1^) and ball milling for another 1 h.

**Scheme 2:**
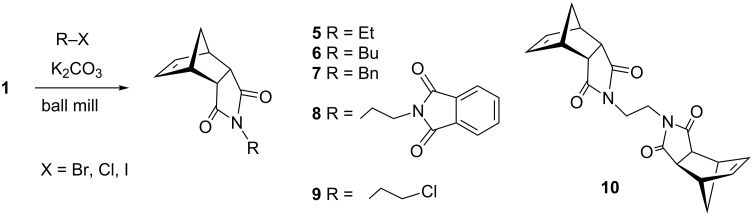
Mechanochemical *N*-alkylation of imide **1**.

The optimized reaction conditions were used to establish the scope of this reaction. Firstly, other alkyl halides were employed (Scheme 2). These experiments revealed that the solvent-free *N*-alkylation could be effectively carried out with different alkyl halides, however, the conditions had to be optimized for each substrate. In particular, reactions carried out by a one-pot, two-step process [28] of **1** with K_2_CO_3_ (producing in situ the potassium imide salt), followed by the addition of the halide and further milling in conjunction with LAG (DMF) proved useful. Ball milling of **1** with alkyl halides afforded after 2 h the corresponding *N*-alkylated products in high yields, with exception of butyl chloride (Table 2, entry 4). The sequential milling procedure is advantageous in terms of the use of smaller amounts of reagents and a significant reduction of the reaction time was achieved in comparison with the reaction in DMF. In contrast to the milling of imide **1** with 1,3-dibromopropane (**2**), the reaction with 1,2-dichloroethane gave products **9** and **10**, where bis-product **10** was the major, despite of large excess of reagent (Table 2, entry 9). The physical state of the halide reagents (either liquid or solid alkyl halides) did not influence the reaction outcome (see Supporting Information File 1, Table S1).

**Table 2:** Mechanochemical *N*-alkylation of imide **1**.

Entry	Halide	Product	Ratio **1**:RX:K_2_CO_3_	Time, conditions	Ratio **1**:product^a^	Yield [%]^b^

1	EtBr	**5**	1:10:5	2 h	0:100	>95
2		**5**	1:2:2	1 + 1 h^c^		87
3	EtI	**5**	1:10:5	2 h	10:90	
4	BuCl	**6**	1:6:2	1 + 1 h^c^	97:3	
5	BnBr	**7**	1:2:2	1 + 1 h^c^		81
6	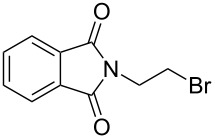	**8**	1:1:5	2 h	65:35	
7		**8**	1:2:4	1 + 1 h^c^		63
8		**8**	1:1:5	72 h^d^		59
9	ClCH_2_CH_2_Cl	**9**,**10**	1:12:5	2 h	51:6:43	

^a^Ratio determined by ^1^H NMR spectroscopy; ^b^isolated yields; ^c^milling of **1** with K_2_CO_3_ for 1 h, followed by the addition of RX and LAG (DMF, η = 2 μL mg^−1^) and ball milling for another 1 h; ^d^DMF, 50 °C, 3d.

**Figure 1:**
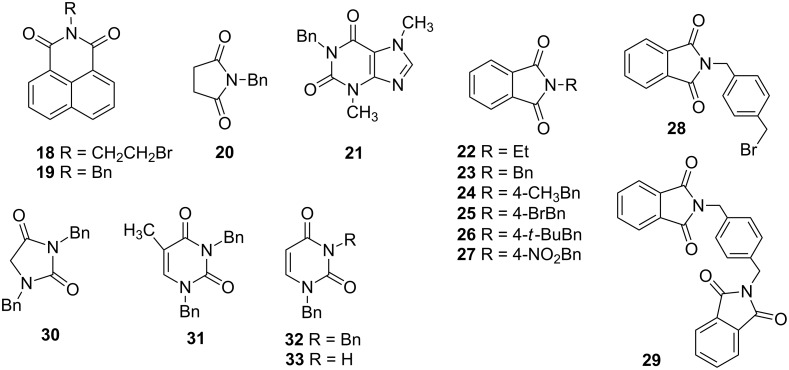
Products of alkylation of imides **11**–**17**.

Further alkylation experiments were carried out with selected imides **11**–**17** (Figure 1, Table 3). The sequential mechanochemical alkylation was found to be often advantageous over the reaction carried out by standard procedure in solvent, either by shorter reaction time, less vigorous conditions or better yields. Another advantage of solvent-free conditions is the circumvention of the problematic low solubility of some of the substrates employed. By conducting the reaction in a ball mill, solubility problems and the issues associated with the selection of the most suitable solvent could be avoided. In addition, solid-state reaction diminishes the heterogeneous character of alkylation, since inorganic bases in general are not soluble in organic solvents.

**Table 3:** Mechanochemical *N*-alkylation of imides **11–17**.^a^

Entry	Substrate	Bromide	Ratio imide:RX:K_2_CO_3_	Product, yield [%]^b^

1 2	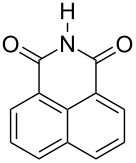 **11**	**2** BnBr	1:2:4 1:2:2	**18**, 73 **19**, 98
3	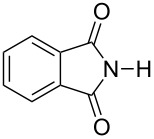 **12**	EtBr	1:2:2	**22, 75**
4		BnBr	1:2:2	**23, 97**
5		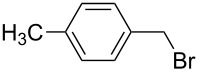	1:2:2	**24**, 94
6		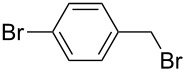	1:2:2	**25**, 95
7		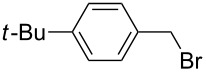	1:2:2	**26**, 90
8		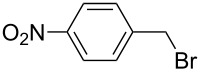	1:2:2	**27**, 98
9		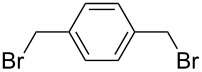	1:2:2	**28**, 32; **29**, 4
10	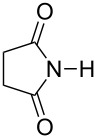 **13**	BnBr	1:2:2	**20**, 89
11	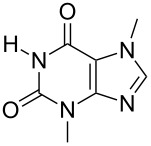 **14**	BnBr	1:2:2	**21**, 67
12	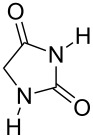 **15**	BnBr	1:4:4	**30**, 93
13	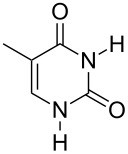 **16**	BnBr	1:4:4	**31**, 99
14 15	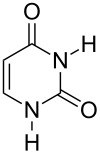 **17**	BnBr	1:2:2 1:1:2	**32**, 98 **32**, 37; **33**, 17

^a^Milling of imides with K_2_CO_3_ for 1 h, followed by the addition of RX and LAG (DMF, η = 2 μL mg^−1^) and ball milling for another 1 h; ^b^isolated yields.

**Figure 2:**
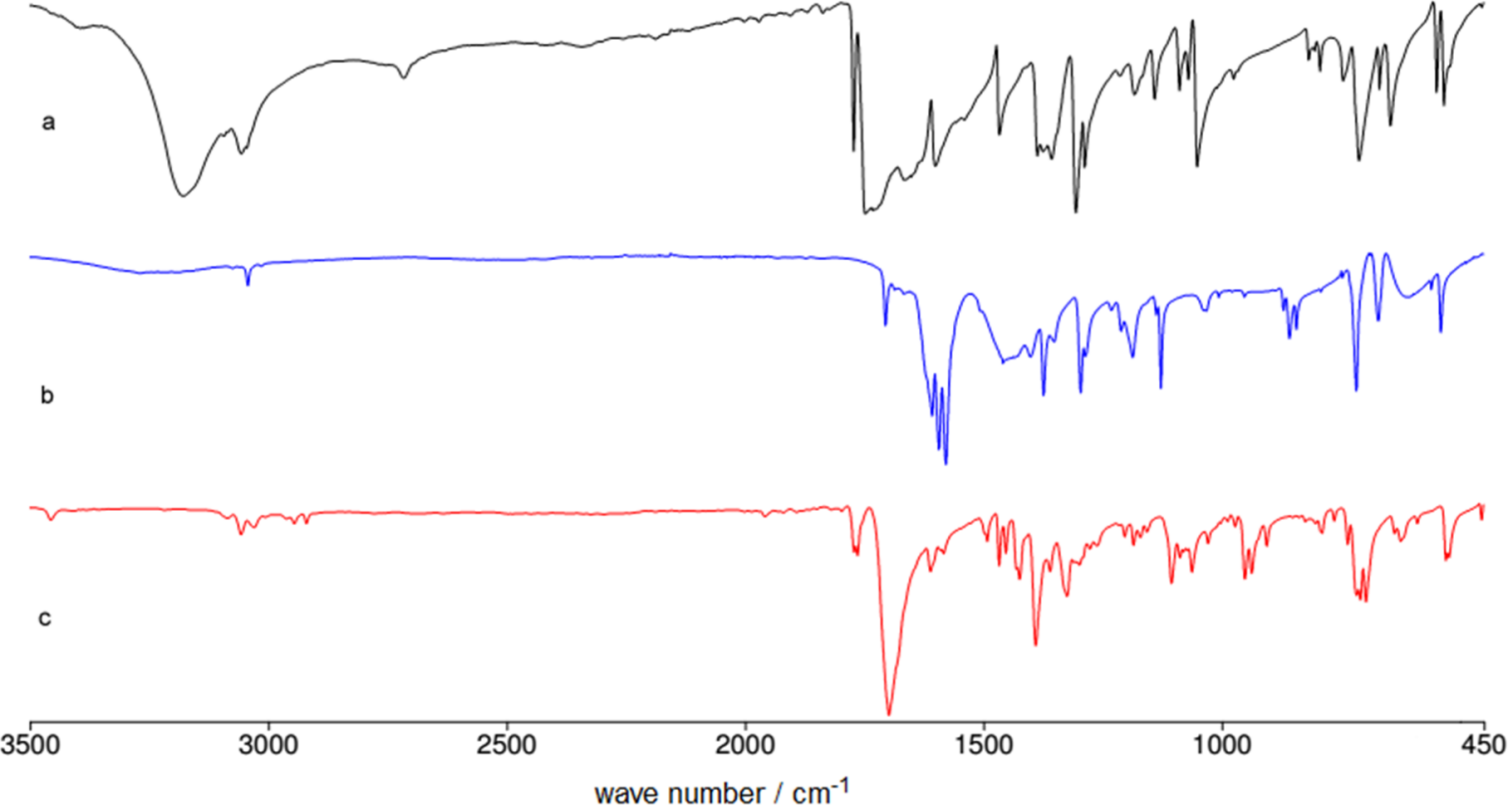
Ex situ IR spectroscopy of the reaction of **12** and benzyl bromide in the ball mill: a) phthalimide **12**; b) first step: phthalimide + K_2_CO_3_, 1 h milling; c) second step: addition of benzyl bromide, LAG DMF and further 1 h milling.

Ex situ IR spectroscopy (ATR) of milling of imides **11**–**17** with K_2_CO_3_ was used for monitoring the reaction progress, which showed for instance, that potassium phthalimide [29] was formed after one hour of grinding (Figure 2). This salt was, without isolation, subjected to further milling with benzyl bromide with LAG (DMF) to obtain alkylated products in high yields. Formation of potassium salts of other imides listed in Table 3 by K_2_CO_3_ has been also proven by ex situ IR monitoring (see Supporting Information File 1). It indicates that potassium carbonate is capable of the deprotonation of the imides with p*K*_a_ values at least within the range of 8.3–9.9 units [30] under ball milling conditions.

Deprotonation of phthalimide in solution is usually carried out with the use of bases stronger than K_2_CO_3_ [31] and this difference in reactivity in comparison to solvent free conditions has a precedence in the application of weaker base in mechanochemical synthesis of triphenylphosphoranes [32]. Often DMF is used as solvent in imide alkylation reactions, which promotes S_N_2 reactions [33] and its low volatility is advantageous over more environmentally friendly solvents which might be considered for LAG in mechanosynthesis.

A comparison of results with literature values demonstrates the benefits of mechanosynthesis. For instance, alkylation of theobromine (**14**) [34] in a microwave reactor in solution gives two side-products, an *O*-alkylated and a uracil ring-opened product (induced by base). The reaction selectivity is highly influenced by the solvent. The formation of the ring-opened product could be fully suppressed under mechanochemical conditions, due to the mild conditions and the absence of solvent. An additional advantage of the solvent-free milling procedure is that there is no need for tetrabutylammonium iodide as phase-transfer catalyst to increase the limited solubility of **14**.

**Scheme 3:**
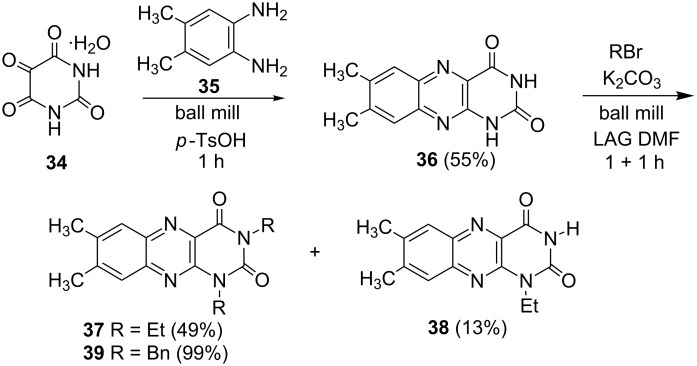
Mechanosynthesis of 7,8-dimethylalloxazine (**36**) and its *N*-alkylation.

The selectivity was observed for certain substrates. For example, the alkylation of phthalimide **12** with 1,4-bis(bromomethyl)benzene led to the formation of two products, namely **28** and **29**. By keeping the ratio of the alkyl halide reagent at two equivalents, ball milling afforded mainly the targeted mono-alkylated product **28** (Table 3, entry 9). The regioselectivity of substrates with two nitrogen-sites available for alkylation could be also controlled by reagent ratio or choice of the alkyl halide. For instance in the reaction of uracil (**17**) or 7,8-dimethylalloxazine (**36**). The required substrate **36** was prepared by mechanochemical condensation [35] of alloxane (**34**) and 4,5-dimethyl-1,2-phenylenediamine (**35**) in the presence of *p*-toluenesulfonic acid [36–37] (Scheme 3). The α-dione/α-diamine reaction proceeds in a similar manner and yield to the condensation reaction carried out under classical reaction conditions (1 M HCl, 60 °C, 30 min) [38]. Mechanochemical one-pot, two-step solid-state *N*-alkylation of **36** with benzyl bromide yielded 1,3-dibenzylalloxazine **39** in quantitative yield, whereas the reaction of **36** with less reactive ethyl bromide (four equivalents) under LAG conditions afforded bis*-* and mono-alkylated products **37** and **38** (in 62% overall yield), with 1,3-diethylalloxazine **37** as the major component (4:1 ratio). A change of the stoichiometry of reagents by milling with an equimolar amount of ethyl bromide resulted in the dominant formation of the mono-alkylated 1-ethyl product **38**.

**Scheme 4:**
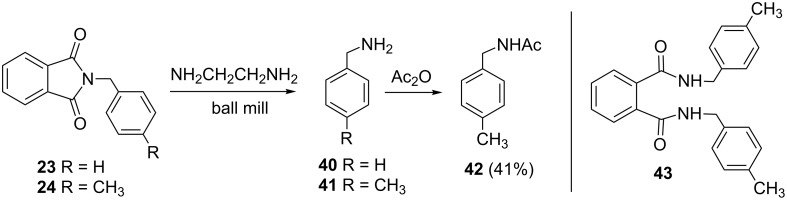
Gabriel synthesis of amines in ball mill.

**Scheme 5:**
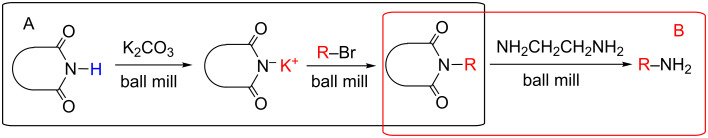
Three-step, two-pot Gabriel synthesis of amines in ball mill.

The *N*-alkylated phthalimides **23** and **24**, which were prepared in the previous section were employed in solvent-free Gabriel synthesis of primary amines (Scheme 4). In these milling reactions, the hazardous hydrazine hydrate was replaced by 1,2-diaminoethane [39] and conversion to the corresponding benzylamines was quantitative within 1 h. As a proof of concept of reaction, *p*-methylbenzylamine was isolated in 41% yield in the form of acetamide **42**. In this way, a three-step, two-pot (A and B, Scheme 5) Gabriel synthesis of amines was carried out in a ball mill. The synthetically desired development of a three-step, one-pot mechanochemical Gabriel synthesis of amines could not be accomplished, as the complex reaction mixtures containing considerable amounts of various side products such as bisamide **43**.

### Computational section

To elucidate reasons for the observed regioselectivities, the reactions of uracil and 7,8-dimethylalloxazine with benzylamine and ethyl bromide were studied by DFT calculations using the B3LYP/6-311+G**//B3LYP/6-31G*+ZPVE method. The transition-state calculations of the S_N_2 reaction of imides and bromides were used to determine the activation energies. It was found that for benzyl and ethyl bromides the activation energy differences are 2–3 kcal mol^−1^ in favor of the N1 positions in uracil and 7,8-dimethylalloxazine. These calculations are in good accordance with the experimentally observed results and could be further rationalized by the more nucleophilic character of these two imide N1 positions in comparison to the N3 positions.

## Conclusion

We have shown that *N*-alkylation of imides could be effectively carried out by ball milling, affording the products in high yields. Effective in situ preparation of potassium phthalimide and its alkylation has a potential for the application in mechanochemical Gabriel synthesis of amines. This account illustrates that organic chemists should explore the advantages of mechanosynthesis and apply this method routinely for screening of the best conditions for various organic reactions.

## Supporting Information

File 1 Additional experimental details, ^1^H, ^13^C NMR and IR spectra.
