# Supplementary material for: Mechanochemical N-alkylation of imides
Source: Beilstein J Org Chem. 2017 Aug 22;13:1745–52. doi: 10.3762/bjoc.13.169 (PMC5588455; doi:10.3762/bjoc.13.169)
Supplement: File 1 — Additional experimental details, 1H, 13C NMR and IR spectra. [file Beilstein_J_Org_Chem-13-1745-s001.pdf]

Supporting information

for

Mechanochemical *N*-alkylation of imides

Anamarija Briš, Mateja Đud and Davor Margetić*§

Address: Laboratory for Physical-organic Chemistry, Division of Organic Chemistry and Biochemistry, Ruđer Bošković Institute, Bijenička c. 54, 10000 Zagreb, Croatia

Email: Davor Margetić - margetid@irb.hr

§Fax: +385-1-456-1008

Additional experimental details, ^{1}H, ^{13}C NMR and IR spectra

Experimental

General remarks

Commercially available reagents were used in all reactions that are not dried nor further purified unless specifically indicated. Reagents were purchased from the following manufacturers of chemicals: Acros, Alfa Aesar or Sigma Aldrich. Solvents were supplied from Kemika or Sigma Aldrich and dryed using standard methods [1]. The mechanochemical reactions are performed in Retsch MM400 vibrating mill at a frequency of 30 Hz in a stainless steel 10 mL vial with one steel ball of 12 mm diameter. For column chromatography silica gel (silica gel 60, 70–230 mesh, Fluka) was used. The prepared compounds were identified by ^{1}H and ^{13}C NMR spectroscopy using a Bruker Avance 300 MHz or a Bruker Avance 600 MHz NMR spectrometer. For NMR spectroscopy commercially available chloroform-*d* and dimethyl sulfoxide-d_6 were used. Chemical shifts (δH and δC) are expressed relative to tetramethylsilane (TMS) as internal standard. Signals are designated as s = singlet, d = doublet, t = triplet, q = quartet, quin = quintet, m = multiplet, dd = doublet of doublets, dt = doublet of triplets. Melting points were measured on a Kofler melting point apparatus and are uncorrected. IR spectra were collected on an Perkin Elmer Spectrum Two FTIR spectrophotometer equipped with UATR (single reflection diamond) at resolution of 4 cm^{-1}. The spectra were recorded with a total of 8 scans using the horizontal single-reflection ATR diamond prism method. Each spectrum was recorded as the ratio of the sample spectrum to the spectrum of empty ATR plate. Imide **1** was prepared by literature method.

(1α,2α,6α,7α)-4-azatricyclo[5.2.1.0^{2,6}]deca-8-ene-3,5-dione (1) [2]:
Yellow-brown solid; m.p. = 183-185 °C; IR (ATR): 1754, 1699 (C=O) cm^{-1}; ^{1}H NMR (300 MHz, DMSO-d_6), *δ*/ppm: 1.56 (1H, d, *J*=8.3 Hz, H10a), 1.63 (1H, dt, *J*=8.3, 1,7 Hz, H10b), 3.23-3.31 (2H, m, H1, H7), 3.34-3.38 (2H, m, H2, H6), 6.20 (2H, t, *J*=1.7 Hz, H8, H9), 10.69 (1H, s NH); ^{13}C NMR (75 MHz, DMSO) *δ*/ppm: 46.3, 48.9, 54.1, 136.6, 181.1.

Mechanochemical synthesis

Typical procedure for ball milling *N*-alkylations

A mixture of 50 mg of imide and the required amount of K_2CO_3 were placed in a 10 mL stainless steel grinding jar and milled for 1 hour at 30 Hz. Upon completion, the required amount of alkyl halide was added and milling was continued for 1 hour in the presence of 100 µL of dry DMF (LAG experiment, $\eta = 2$ µL mg^{-1}). The obtained mixture was suspended in dichloromethane and washed with water. The organic layers were collected and the solvent was evaporated. Where it was necessary, the products were separated by using column chromatography.

(1α,2α,6α,7α)-4-(3-Bromopropyl)-4-azatricyclo[5.2.1.0^{2,6}]dec-8-ene-3,5-dione (3) [3]:
Yellow oil; IR (ATR): 1766, 1694 (C=O) cm^{-1}; ^{1}H NMR (300 MHz, $CDCl_3$), *δ*/ppm: 1.54 (1H, d, *J*=8.9 Hz, H10a), 1.74 (1H, dt, *J*=8.9, 1.7 Hz, H10b), 2.02 (2H, quin, *J*=6.9 Hz, H2'a,b), 3.25-3.27 (2H, m, H1, H7), 3.29 (2H, t, *J*=6.9 Hz, H3'a,b), 3.36-3.42 (2H, m, H2, H6), 3.47 (2H, t, *J*=6.9 Hz, H1'a,b), 6.10 (2H, t, *J*=1.7 Hz, H8, H9); ^{13}C NMR (75 MHz, $CDCl_3$) *δ*/ppm: 29.6, 30.8, 36.9, 44.8, 45.6, 52.1, 134.3, 177.4.

1',3'-bis[(1α,2α,6α,7α)-4-Azatricyclo[5.2.1.0^{2,6}]dec-8-ene-3,5-dione]propane (**4**):
Yellow-brown crystals; m.p. =153-155 °C; IR (ATR): 1767, 1686 (C=O) cm^{-1}; ^{1}H NMR (300 MHz, $CDCl_3$), *δ*/ppm: 1.50 (2H, d, *J*=8.9 Hz, H10a), 1.55 (2H, t, *J*=7.1 Hz, H2'a,b) 1.70 (2H, dt, *J*=8.9, 1.7 Hz, H10b), 3.19-3.22 (4H, m, H1, H7), 3.27 (4H, t, *J*=7.1 Hz, H1'a,b), 3.32-3.38 (4H, m, H2, H6), 6.12 (4H, t, *J*=1.7 Hz, H8, H9); ^{13}C NMR (150 MHz, $CDCl_3$) *δ*/ppm: 26.6, 36.3, 45.1, 45.9, 52.4, 134.7, 177.9.

(1α,2α,6α,7α)-4-Ethyl-4-azatricyclo[5.2.1.0^{2,6}]dec-8-ene-3,5-dione (5) [4]:
Light yellow solid; m.p. = 50-52 °C; IR (ATR): 1775, 1684 (C=O) cm^{-1}; ^{1}H NMR (300 MHz, $CDCl_3$), *δ*/ppm: 1.01 (3H, t, *J*=7.1 Hz, H2'), 1.50 (1H, d, *J*=8.9 Hz, H10a), 1.70 (1H, dt, *J*=8.9, 1.7 Hz, H10b), 3.17-3.24 (2H, m, H1, H7), 3.32-3.42 (4H, m, H2, H6, H1'a,b), 6.06 (2H, t, *J*=1.7 Hz, H8, H9); ^{13}C NMR (150 MHz, $CDCl_3$) *δ*/ppm: 13.3, 33.5, 45.2, 45.9, 52.4, 134.5, 177.8.

(1α,2α,6α,7α)-4-Benzyl-4-azatricyclo[5.2.1.0^{2,6}]dec-8-ene-3,5-dione (7) [5]:
Light yellow crystals; m.p. = 70-72 °C; IR (ATR): 1767, 1688 (C=O) cm^{-1}; ^{1}H NMR (600 MHz, $CDCl_3$), *δ*/ppm: 1.50 (1H, d, *J*=8.9 Hz, H10a), 1.70 (1H, dt, *J*=8.9, 1.7 Hz, H10b), 3.23-3.27 (2H, m, H1, H7), 3.35-3.39 (2H, m, H2, H6), 4.48 (2H, s, H1'a,b), 5.9 (2H, t, *J*=1.7 Hz, H8, H9), 7.23-7-32 (5H, m, *Ar*); ^{13}C NMR (75 MHz, $CDCl_3$) *δ*/ppm: 42.2, 45.2, 45.9, 52.3, 127.9, 128.6, 129.1, 134.5, 136.2, 177.6.

(1α,2α,6α,7α)-4-(2'-Ethylphthalimido)-4-azatricyclo[5.2.1.0^{2,6}]dec-8-ene-3,5-dione (8) [6]:
Colorless solid; m.p. = 150-152 °C; IR (ATR): 1772, 1696 (C=O) cm^{-1}; ^{1}H NMR (300 MHz, $CDCl_3$), *δ*/ppm: 1.47 (1H, d, *J*=8.9 Hz, H10a), 1.66 (1H, dt, *J*=8.9, 1.7 Hz, H10b), 3.18-3.22 (2H, m, H1, H7), 3.24-3.30 (2H, m, H2, H6), 3.61-3.68 (2H, m, H1'a,b), 3.76-3.83 (2H, m, H2'a,b), 6.00 (2H, t, *J*=1.7 Hz, H8, H9), 7.65-7.71 (2H, m, H5", H6"), 7.76-7.83 (2H, m, H4", H7"); ^{13}C NMR (75 MHz, $CDCl_3$) *δ*/ppm: 36.5, 37.0, 44.8, 46.1, 52.5, 123.5, 132.1, 134.2, 134.6, 168.4, 177.8.

***N*-(3-Bromopropyl)-1,8-naphthalimide (18)** [7]:
Light pink solid; m.p. = 129-132 °C; IR (ATR): 1698, 1675 (C=O) cm^{-1}; ^{1}H NMR (300 MHz, $CDCl_3$), *δ*/ppm: 2.34 (2H, quin, *J*=6.9 Hz, H2'a,b), 3.51 (2H, t, *J*=6.9 Hz, H3'a,b), 4.34 (2H, t, *J*=6.9 Hz, H1'a,b), 7.76 (2H, t, *J*=7.4 Hz, H3, H6), 8.22 (2H, d, *J*=7.4 Hz, H4, H5), 8.61 (2H, d, *J*=7.4 Hz, H2, H7); ^{13}C NMR (150 MHz, $CDCl_3$) *δ*/ppm: 30.7, 31.7, 39.5, 122.7, 127.2, 128.4, 131.6, 131.8, 134.3 164.5.

***N*-Benzyl-1,8-naphthalimide (19)** [8]:
Pale orange solid; m.p. = 195-197 °C; IR (ATR): 3065, 3040, 2970, 1690, 1652, 1624, 1583, 1512, 1497, 1456, 1436, 1378, 1362, 1348, 1333, 1314, 1234, 1176, 1145, 1070, 1025, 948, 846, 824, 776, 760, 737, 697, 647, 597, 528, 494.7 cm^{-1};
^{1}H NMR (300 MHz, $CDCl_3$), *δ*/ppm: 5.39 (2H, s, CH_2), 7.23-7.34 (3H, m, ArH-phenyl), 7.55 (2H, d, *J*=6.97 Hz, ArH-phenyl), 7.73-7.78 (2H, m, naphthyl-H4,5), 8.21 (2H, dd, *J*=8.4, 1.1 Hz, naphthyl-H2,7), 8.62 (2H, dd, *J*=7.4, 1.1 Hz, naphthyl-H3,6).

1-(Phenylmethyl)-2,5-pyrrolidinedione (20) [9]:
White crystals; m.p. = 97-99 °C; IR (ATR): 3455, 3063, 2925, 1773, 1688, 1499, 1454, 1424, 1405, 1361, 1332, 1255, 1164, 1055, 1000, 921, 878, 820, 719, 692, 659, 638, 457 cm^{-1};
^{1}H NMR (300 MHz, $CDCl_3$), *δ*/ppm: 2.73 (4H, s, CH_2CH_2), 4.68 (2H, s, CH_2Ph), 7.29-7.35 (3H, m, Ar-H3',4',5'), 7.41 (2H, d, *J*=7.4 Hz, Ar-H2',6').

***1*-*N*-Benzyltheobromine (21)** [10]:
Purified on silica gel (CH_2Cl_2) to obtain colorless oil; IR (ATR): 3110, 2950, 1697, 1653, 1602, 1545, 1484, 1452, 1430, 1368, 1350, 1319, 1283, 1231, 1180, 1121, 1073, 949, 759, 747, 703, 647, 616, 605, 504, 463 cm^{-1}; ^{1}H NMR (300 MHz, $CDCl_3$), *δ*/ppm: 3.56 (3H, s, 3-NCH_3), 3.96 (3H, s, 7-NCH_3), 5.19 (2H, s, 1-NCH_2), 7.22-7.25 (1H, m, ArH), 7.27-7.30 (2H, m, ArH), 7.48 (2H, d, *J*=7.3 Hz, ArH), 7.47 (1H, s, H8); ^{13}C NMR (150 MHz, $CDCl_3$) *δ*/ppm: 29.7 (-NCH_3), 33.6 (-NCH_3), 44.4 (1-NCH_2), 107.7, 127.5, 128.4, 128.8, 137.3, 141.6, 148.9, 151.6 (C=O), 155.2 (C=O).

***N*-Ethyl-1*H*-isoindol-1,3(2*H*)-dione (22)** [11]:
White crystals; m.p. =66-68 °C; IR (ATR): 1773, 1710 (C=O) cm^{-1}; ^{1}H NMR (300 MHz, $CDCl_3$), *δ*/ppm: 1.24 (3H, t, *J*=7.2 Hz, H2'), 3.70 (2H, q, *J*=7.2 Hz, H1'a,b), 7.66 (2H, dd, *J*=5.6, 3.1 Hz, H6, H7), 7.79 (2H, dd, *J*=5.6, 3.1 Hz, H5, H8); ^{13}C NMR (75 MHz, $CDCl_3$) *δ*/ppm: 14.1, 33.1, 123.3, 132.4, 134.0, 168.4.

2-Benzyl-1*H*-isoindol-1,3(2*H*)-dione (23) [12,13]:
White crystals; m.p. = 112-114 °C (lit. 116 °C); IR (ATR): 3464, 3060, 2916, 2849, 1764, 1705, 1611, 1583, 1491, 1466, 1452, 1431, 1389, 1330, 1297, 1184, 1102, 1086, 1062, 936, 792, 763, 715, 695, 623, 521 cm^{-1}; ^1H NMR (300 MHz, $CDCl_3$), δ/ppm: 4.85 (2H, s, CH_2), 7.24-7.32 (3H, m, ArH-phenyl), 7.43 (2H, d, *J*=7.26 Hz, ArH-phenyl), 7.69 (2H, dd, *J*=6.1, 3.1 Hz, ArH-phthalimido), 7.84 (2H, dd, *J*=6.1, 3.1 Hz, ArH-phthalimido).

2-(4-Methyl-benzyl)-1*H*-isoindol-1,3(2*H*)-dione (24) [12]:
Purified on silica gel (CH_2Cl_2) to obtain white crystals; m.p. = 118-120 °C (lit. 117-118 °C); IR (ATR): 3468, 3059, 3033, 2939, 1764, 1711, 1613, 1510, 1465, 1427, 1390, 1332, 1302, 1180, 1083, 933, 849, 807, 751, 712, 618, 572, 529, 467 cm^{-1}; ^1H NMR (300 MHz, $CDCl_3$), δ/ppm: 2.32 (3H, s, CH_3), 4.82 (2H, s, CH_2), 7.13 (2H, d, *J*=7.9 Hz, ArH-phenyl), 7.34 (2H, d, *J*=7.9 Hz, ArH-phenyl), 7.71 (2H, dd, *J*=5.6, 2.7 Hz, ArH-phthalimido), 7.85 (2H, dd, *J*=5.5, 2.7 Hz, ArH-phthalimido).

2-(4-Bromobenzyl)-1*H*-isoindol-1,3(2*H*)-dione (25) [14]:
Purified on silica gel (CH_2Cl_2) to obtain white crystals; m.p. = 129-131 °C (lit. 128 °C); IR (ATR): 3464, 3106, 3044, 2938, 1769, 1700, 1486, 1465, 1392, 1336, 1298, 1172, 1083, 1069, 1008, 957, 934, 848, 792, 728, 711, 642, 548, 529, cm^{-1}; ^1H NMR (300 MHz, $CDCl_3$), δ/ppm: 4.79 (2H, s, CH_2), 7.32 (2H, d, *J*=8.4 Hz, ArH-phenyl), 7.44 (2H, d, *J*=8.4 Hz, ArH-phenyl), 7.72 (2H, dd, *J*=5.8, 3.2 Hz, ArH-phthalimido), 7.85 (2H, dd, *J*=5.8, 3.2 Hz, ArH-phthalimido); ^{13}C NMR (150 MHz, $CDCl_3$) δ/ppm: 40.9, 121.9, 123.4, 130.4, 131.8, 132.0, 134.1, 135.3, 167.9.

2-(4-(*tert*-Butyl)benzyl)-1*H*-isoindol-1,3(2*H*)-dione (26) [15]:
Purified on silica gel (CH_2Cl_2 / petroleum ether 1:1) to obtain white crystals; m.p. = 81-83 °C; IR (ATR): 2956, 2868, 1768, 1702, 1513, 1465, 1435, 1393, 1333, 1305, 1281, 1169, 1086, 939, 854, 841, 807, 753, 716, 621, 578, 529 cm^{-1}; ^1H NMR (300 MHz, $CDCl_3$), δ/ppm: 1.28 (9H, s, *t*-Bu), 4.82 (2H, s, CH_2), 7.33 (2H, d, *J*=8.3 Hz, ArH-phenyl), 7.38 (2H, d, *J*=8.3 Hz, ArH-phenyl), 7.69 (2H, dd, *J*=5.7, 3.1 Hz, ArH-phthalimido), 7.84 (2H, dd, *J*=5.7, 3.1 Hz, ArH-phthalimido).

2-(4-Nitrobenzyl)-1*H*-isoindol-1,3(2*H*)-dione (27) [16]:
Purified on silica gel (CH_2Cl_2 / petroleum ether 1:1) to obtain pale yellow crystals; m.p. = 167-169 °C (Lit. 173-174°C); IR (ATR): 3464, 3074, 2931, 1766, 1697, 1599, 1508, 1466, 1422, 1391, 1343, 1327, 1205, 1185, 1101, 1088, 1012, 964, 943, 858, 833, 790, 744, 723, 710, 693, 641, 612, 558, 529 (C=O) cm^{-1}; ^1H NMR (300 MHz, $CDCl_3$), δ/ppm: 4.94 (2H, s, CH_2), 7.58 (2H, d, *J*=8.8 Hz, ArH-phenyl), 7.75 (2H, dd, *J*=5.5, 3.1 Hz, ArH-phthalimido), 7.88 (2H, dd, *J*=5.5, 3.1 Hz, ArH-phthalimido), 8.18 (2H, d, *J*=8.8 Hz, ArH-phenyl).

2-{[4-(Bromomethyl)phenyl]methyl}-1*H*-isoindol-1,3(2*H*)-dione (28) [17]:
Purified on silica gel (CH_2Cl_2 / petroleum ether 1:1) to obtain white crystals; m.p. = 147-149 °C; IR (ATR): 3468, 3053, 2938, 1766, 1714, 1464, 1432, 1390, 1331, 1229, 1084, 941, 761, 716, 623, 594, 529 cm^{-1}; ^1H NMR (300 MHz, $CDCl_3$), δ/ppm: 4.45 (2H, s, CH_2Br), 4.84 (2H, s, CH_2N), 7.34 (2H, d, *J*=8.3 Hz, ArH-phenyl), 7.41 (2H, d, *J*=8.3 Hz, ArH-phenyl), 7.71 (2H, dd,

J=5.3, 3.2 Hz, ArH-phthalimido), 7.85 (2H, dd, *J*=5.3, 3.2 Hz, ArH-phthalimido); ^{13}C NMR (150 MHz, $CDCl_3$) δ/ppm: 33.0, 41.2, 123.4, 129.1, 129.4, 132.1, 134.0, 136.6, 137.4, 167.9.

α,α-4-Bis(1*H*-isoindol-1,3(2*H*)-dione)xylene (29) [18]:
Purified on silica gel (CH_2Cl_2 / petroleum ether 1:1) to obtain white solid; IR (ATR): 3468, 3053, 2938, 1766, 1714, 1464, 1432, 1390, 1331, 1229, 1084, 941, 761, 716, 623, 594, 529 cm^{-1};
^{1}H NMR (300 MHz, $CDCl_3$), δ/ppm: 4.80 (4H, s, CH_2), 7.38 (4H, s, ArH-phenyl), 7.69 (4H, dd, *J*=5.6, 3.0 Hz, ArH-phthalimido), 7.82 (4H, dd, *J*=5.6, 3.0 Hz, ArH-phthalimido). (obtained from crude spectrum)

1,3-Dibenzylimidazolidin-2,4-dione (30) [19]:
Purified on silica gel (CH_2Cl_2) to obtain colorless oil; IR (ATR): 3066, 3029, 2961, 2923, 2852, 716, 1494, 1452, 1226, 1145, 1205, 1070, 1031, 824, 756, 693, 635, 606, 570, 550 cm^{-1}; ^{1}H NMR (300 MHz, $CDCl_3$), δ/ppm: 3.72 (2H, s, CH_2), 4.55 (2H, s, NCH_2), 4.68 (2H, s, NCH_2), 7.22-7.24 (2H, m, ArH), 7.29-7.37 (6H, m, ArH), 7.42-7.44 (2H, m, ArH).

1,3-Dibenzylthymine (31) [10]:
Purified on silica gel (CH_2Cl_2) to obtain colorless oil; IR (ATR): 3063, 3031, 2951, 1697, 1661, 1637, 1584, 1495, 1463, 1448, 1348, 1251, 1216, 1076, 1029, 983, 823, 765, 733, 698, 603, 527, 486 cm^{-1}; ^{1}H NMR (300 MHz, $CDCl_3$), δ/ppm: 1.92 (3H, s, CH_3), 4.93 (2H, s, CH_2), 5.19 (2H, s, CH_2), 6.98 (1H, d, *J*=1.1 Hz, H6), 7.28-7.40 (8H, m, ArH), 7.52 (2H, d, *J*=7.04 Hz, ArH); ^{13}C NMR (150 MHz, $CDCl_3$) δ/ppm: 13.1, 44.7, 52.0, 110.4, 127.6, 127.9, 128.3, 128.4, 129.0, 129.1, 135.8, 137.1, 138.1, 151.8, 163.6.

1,3-Dibenzyluracil (32) [20]:
Colorless oil; IR (ATR): 3066, 3031, 2951, 1702, 1654, 1585, 1494, 1448, 1390, 1366, 1336, 1219, 1088, 1029, 802, 734, 697, 595, 536, 515 cm^{-1}; ^{1}H NMR (300 MHz, $CDCl_3$), δ/ppm: 4.91 (2H, s, CH_2), 5.14 (2H, s, CH_2), 5.74 (1H, d, *J*=7.8 Hz, H5), 7.11 (1H, d, *J*=7.8 Hz, H6), 7.24-7.38 (8H, m, ArH), 7.48 (2H, d, *J*=7.27 Hz, ArH); ^{13}C NMR (150 MHz, $CDCl_3$) δ/ppm: 44.4, 52.3, 102.1, 127.6, 128.0, 128.4, 128.5, 128.9, 129.1, 135.3, 136.9, 141.9, 151.8, 162.9.

1-Benzyluracil (33) [21]:
Purified on silica gel (CH_2Cl_2 / ethyl acetate 1:1) to obtain brown crystals; m.p. = 169-174 °C; IR (ATR): 3115, 3092, 3003, 2925, 2855, 2811, 1663, 1496, 1462, 1434, 1404, 1380, 1341, 1295, 1241, 1198, 1174, 953, 889, 830, 768, 757, 726, 689, 625, 594, 550, 517 cm^{-1}; ^{1}H NMR (300 MHz, $CDCl_3$), δ/ppm: 4.92 (2H, s, CH_2), 5.69 (1H, dd, *J*=7.8, 2.2 Hz, H5), 7.15 (1H, d, *J*=7.8 Hz, H6), 7.21-7.31 (2H, m, ArH), 7.34-7.42 (3H, m, ArH), 8.56 (1H, brs, NH); ^{13}C NMR (150 MHz, $CDCl_3$) δ/ppm: 51.2, 102.7, 128.1, 128.6, 129.2, 143.8.

7,8-Dimethyl-1,3-diethylalloxazine (37) [22]:
Yellow solid; m.p. =168-170 °C; IR (ATR): 1723, 1670 (C=O) cm^{-1}; ^{1}H NMR (300 MHz, $CDCl_3$), δ/ppm: 1.29 (3H, t, *J*=7.0 Hz, H2'), 1.34 (3H, t, *J*=7.0 Hz, H2''), 2.45 (3H, s, CH_3), 2.47 (3H, s, CH_3), 4.19 (2H, q, *J*=7.0 Hz, H1'a,b), 4.44 (2H, q, *J*=7.0 Hz, H1''a,b), 7.72 (1H, s, H6), 7.98 (1H, s, H9); ^{13}C NMR (150 MHz, $CDCl_3$) δ/ppm: 13.1, 13.3, 20.4, 21.0, 37.8, 38.0, 127.0, 129.0, 129.5, 139.2, 139.9, 142.6, 144.7, 145.5, 150.0, 159.8.

7,8-Dimethyl-1-ethylalloxazine (38) [22]:
Yellow solid; m.p. = 300-302 °C; IR (ATR): 1717, 1701 (C=O) cm^{-1}; ^{1}H NMR (600 MHz, DMSO-d_6), δ/ppm: 1.25 (3H, t, *J*=7.0 Hz, H2'a,b), 2.47 (3H, s, CH_3), 2.49 (3H, s, CH_3), 4.23 (2H, q, *J*=7.0 Hz, H1'a,b), 7.78 (1H, s, H6), 7.91 (1H, s, H9), 11.89 (1H, s, NH); ^{13}C NMR (75 MHz, DMSO-d_6) δ/ppm: 12.6, 19.6, 20.1, 36.2, 126.3, 128.5, 130.7, 137.5, 139.1, 141.0, 144.8, 145.7, 149.7, 159.6.

1,3-Dibenzyl-7,8-dimethyl-alloxazine (39) [23]:
Purified on silica gel (CH_2Cl_2 / petroleum ether 1:1) to obtain orange solid; m.p. = 196-198 °C; IR (ATR): 3031, 1721, 1671, 1551, 1478, 1444, 1383, 1356, 1296, 1252, 1229, 1059, 1001, 877, 722, 694, 576, 515, 484 cm^{-1}; ^{1}H NMR (300 MHz, $CDCl_3$), δ/ppm: 2.49 (3H, s, CH_3), 2.53 (3H, s, CH_3), 5.36 (2H, s, CH_2), 5.62 (2H, s, CH_2), 7.26-7.32 (6H, m, ArH), 7.56-7.62 (4H, m, ArH), 7.79 (1H, s, allox), 8.03 (1H, s, allox); ^{13}C NMR (150 MHz, $CDCl_3$) δ/ppm: 20.3, 20.9, 45.4, 45.5, 126.9, 127.8, 127.9, 128.4, 128.5, 128.7, 129.0, 129.4, 129.5, 136.5, 136.6, 139.4, 140.1, 142.3, 144.6, 145.7, 150.6, 159.9.

4-Methylbenzylacetamide (**42**) [24]:
The mixture obtained by deprotection in ball-mill was transferred with dry methanol to a round bottom flask and treated with acetic anhydride/triethylamine at rt overnight.[25] The excess of anhydride was removed by vacuum distillation and the residue was subjected to column chromatography on silica gel (ethyl acetate/hexane 1:3) to obtain **42** (13.7 mg, 41%) as white crystals; m.p. = 96-98 °C; IR (ATR): 3289, 2923, 2854, 1742, 1633, 1548, 1516, 1462, 1373, 1355, 1315, 1288, 1093, 1023, 804, 726, 599, 546, 477 cm^{-1}; ^{1}H NMR (300 MHz, $CDCl_3$), δ/ppm: 2.01 (3H, s, CH_3, acetyl), 2.34 (3H, s, 4-CH_3), 4.39 (2H, d, *J*=5.7 Hz, CH_2), 5.69 (1H, brs, NH), 7.14 (2H, d, *J*=8.5 Hz, ArH), 7.18 (2H, d, *J*=8.5 Hz, ArH).

7,8-Dimethylalloxazine (36) [22]:
Mixture of alloxan monohydrate (100 mg, 0.62 mmol), 4,5-dimethyl-1,2-phenylenediamine (85 mg, 0.62 mmol) and *p*-toluenesulfonic acid (119 mg, 0.62 mmol) was placed in a stainless steel grinding jar (10 mL) with one 12 mm stainless steel ball and milled for one hour at 30 Hz. Upon completion, the reaction mixture was washed with methanol and filtered. The product was obtained as yellow solid (83 mg, 55%). The spectroscopic data correspond to the literature values.
Yellow solid; m.p. >303 °C; IR (ATR): 1715, 1697 (C=O) cm^{-1}; ^{1}H NMR (300 MHz, DMSO-d_6), δ/ppm: 2.46 (3H, s, CH_3), 2.48 (3H, s, CH_3), 7.69 (1H, s, H6), 7.91 (1H, s, H9), 11.66 (1H, s, NH), 11.83 (1H, s, NH).

NMR SPECTRA

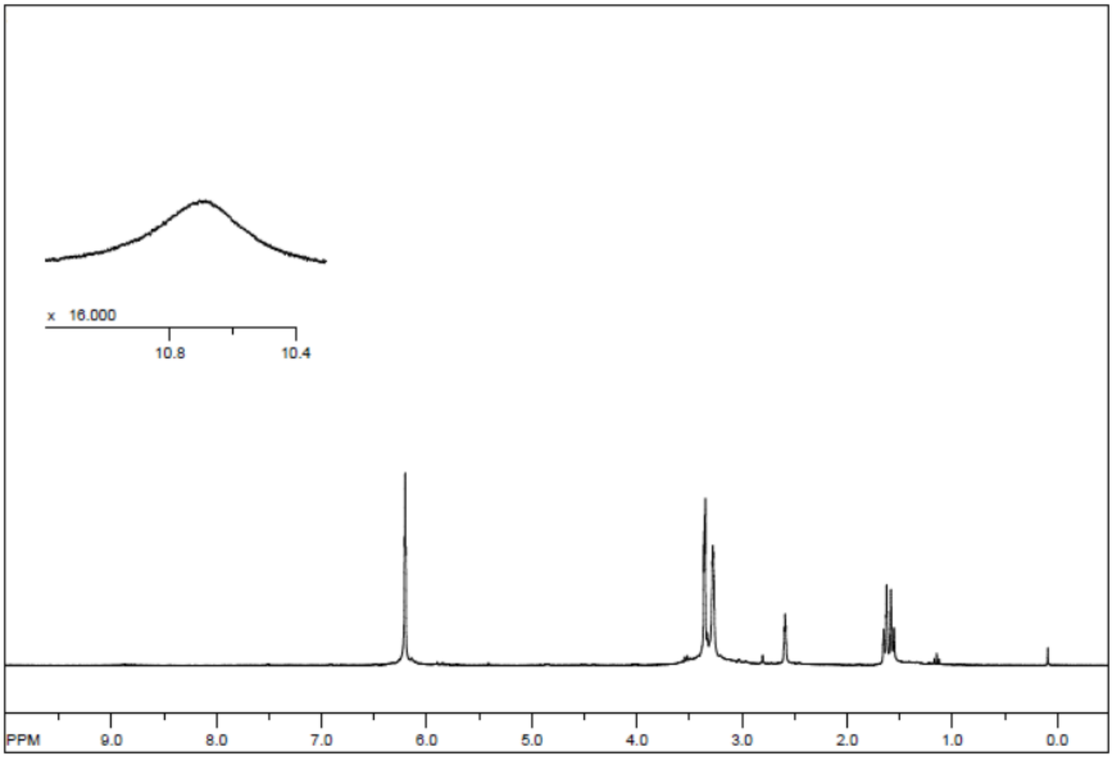

Figure S1. ^{1}H NMR spectrum of **1** in DMSO-d_6

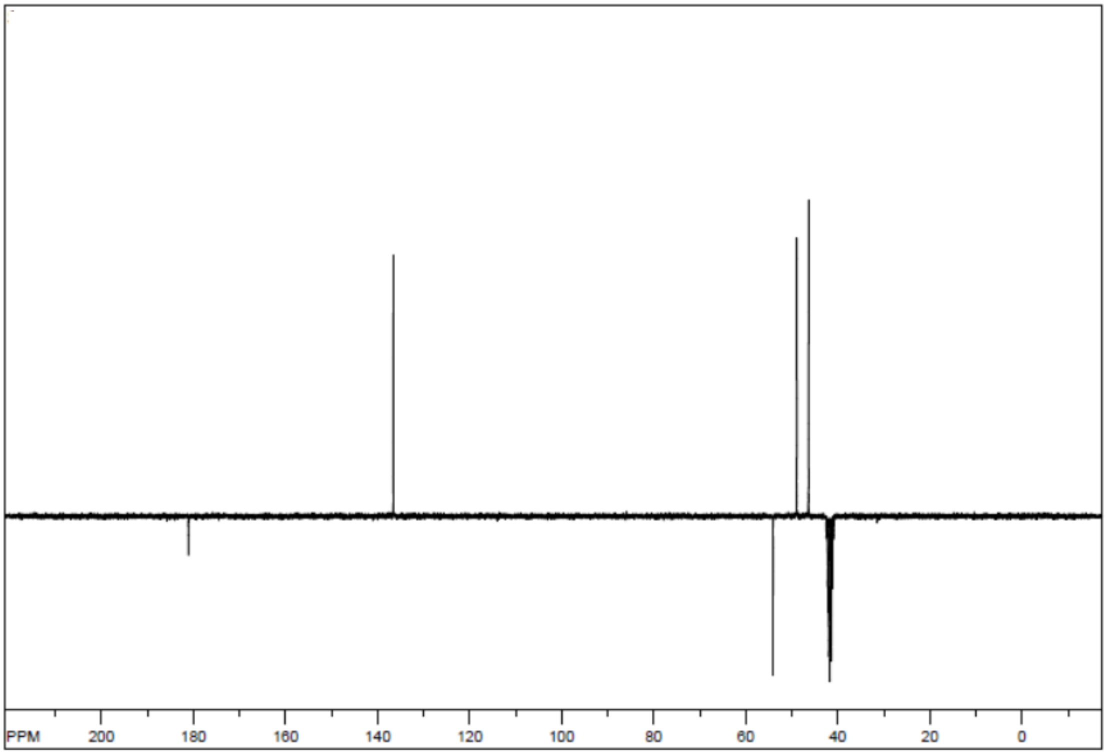

Figure S2. ^{13}C NMR spectrum of **1** in DMSO-d_6

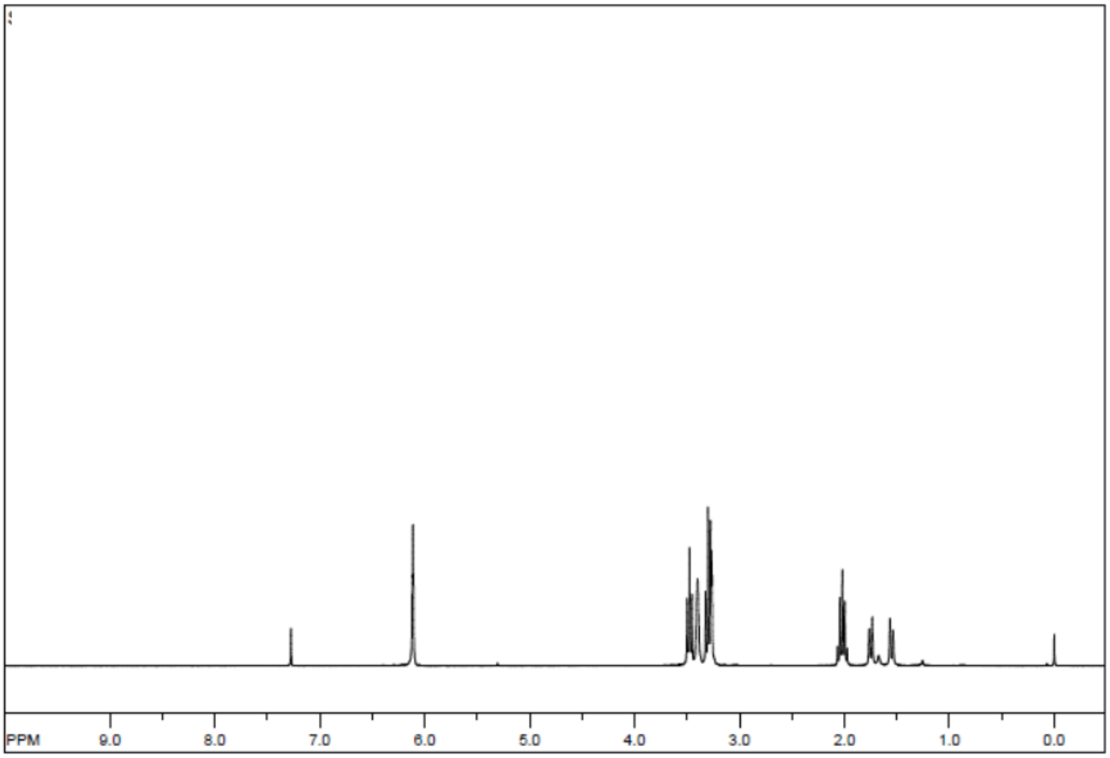

Figure S3. ^{1}H NMR spectrum of **3** in $CDCl_3$

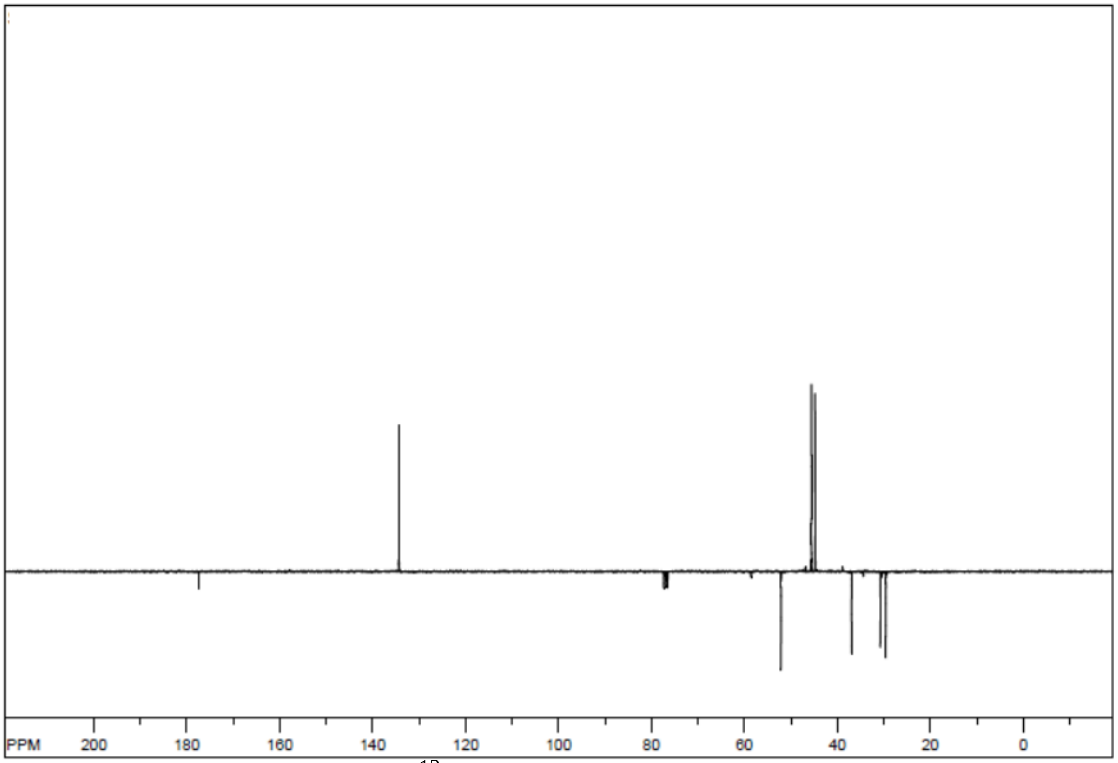

Figure S4. ^{13}C NMR spectrum of **3** in $CDCl_3$

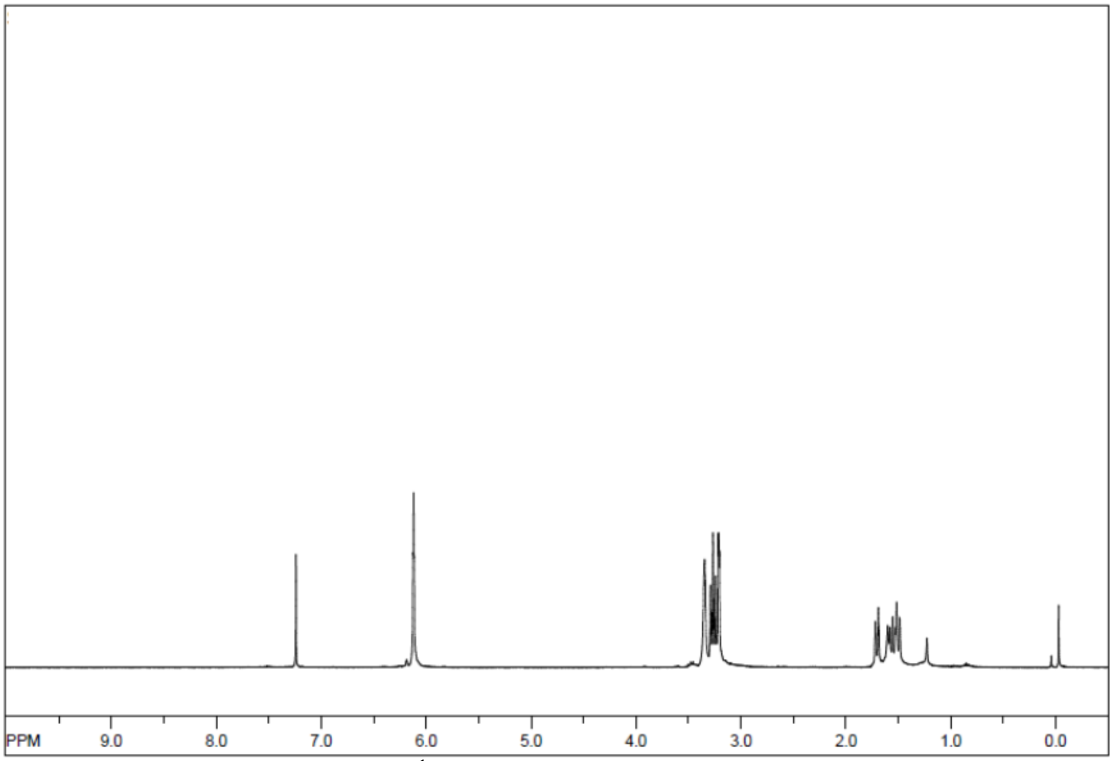

Figure S5. ^{1}H NMR spectrum of **4** in $CDCl_3$

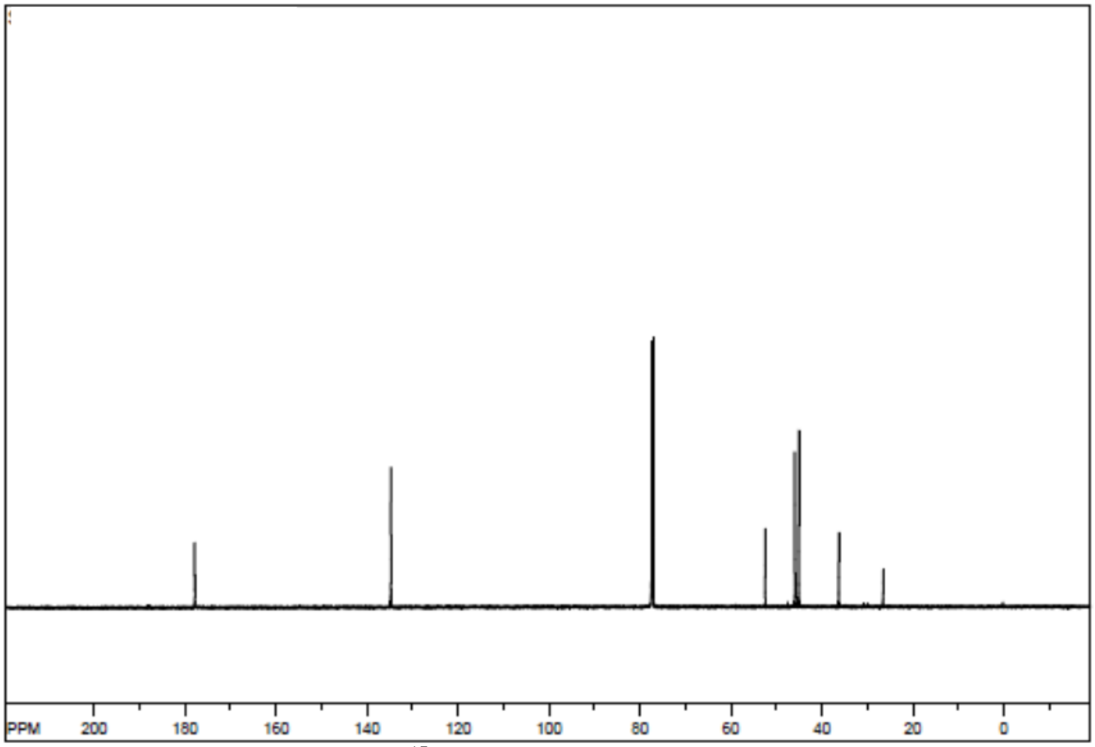

Figure S6. ^{13}C NMR spectrum of **4** in $CDCl_3$

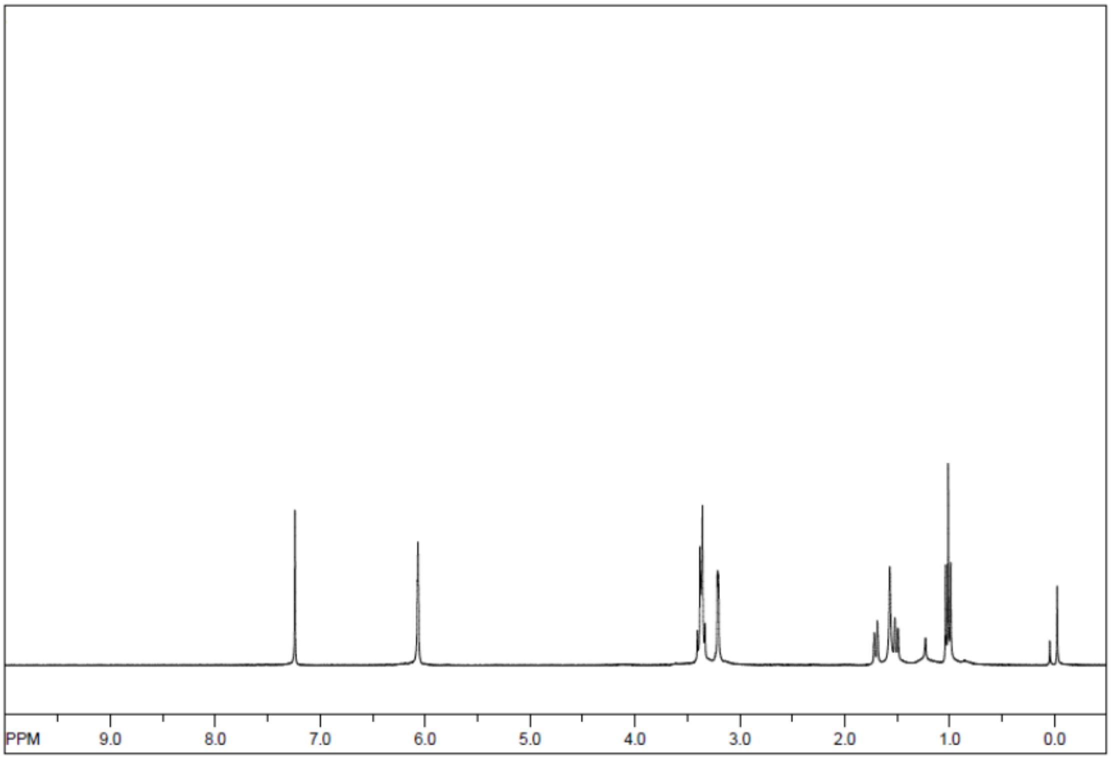

Figure S7. ^{1}H NMR spectrum of **5** in $CDCl_3$

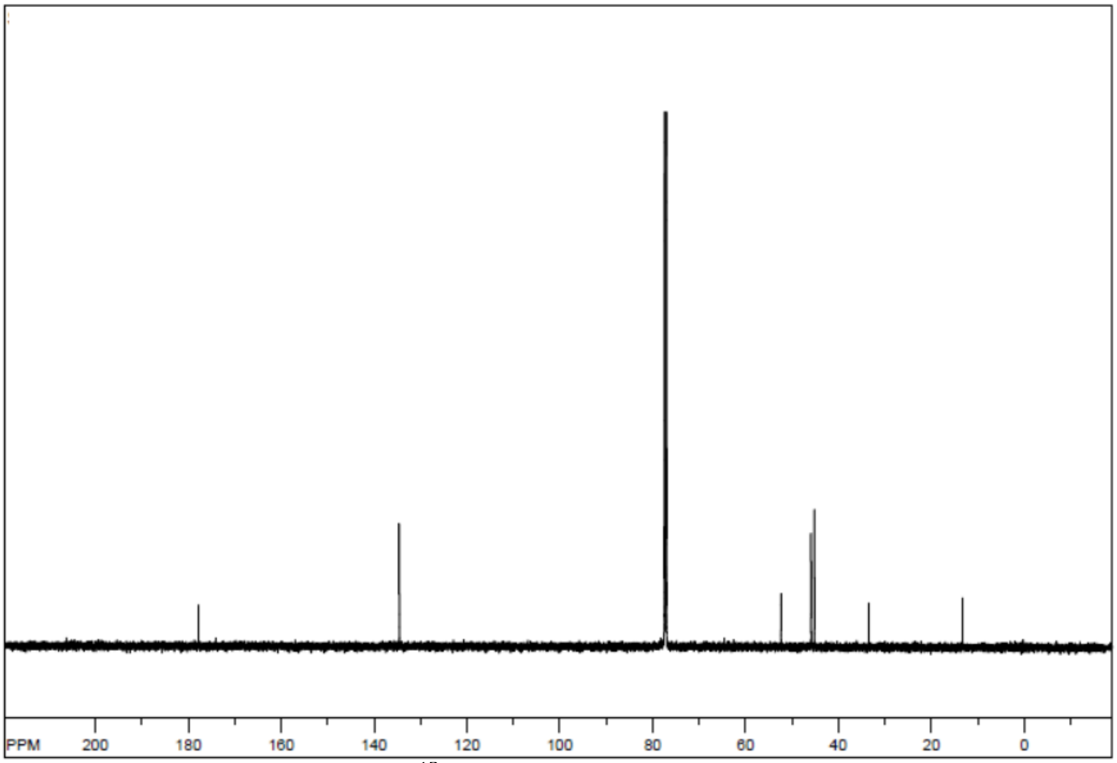

Figure S8. ^{13}C NMR spectrum of **5** in $CDCl_3$

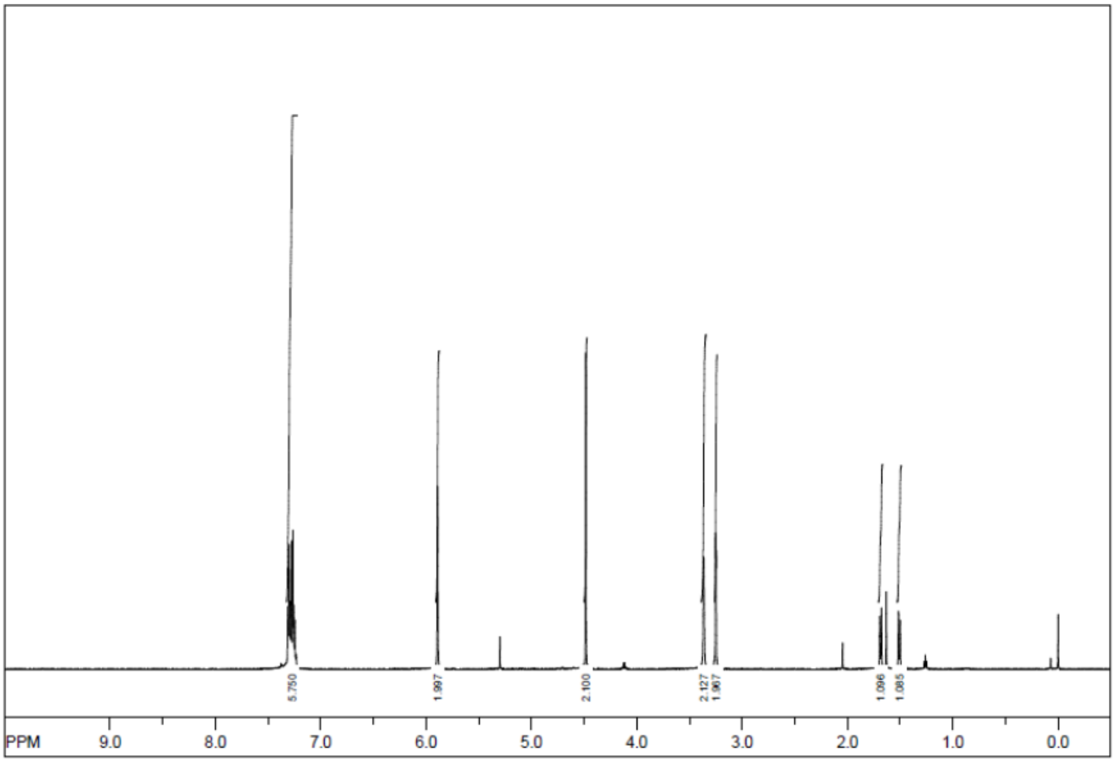

Figure S9. ^{1}H NMR spectrum of **7** in $CDCl_3$

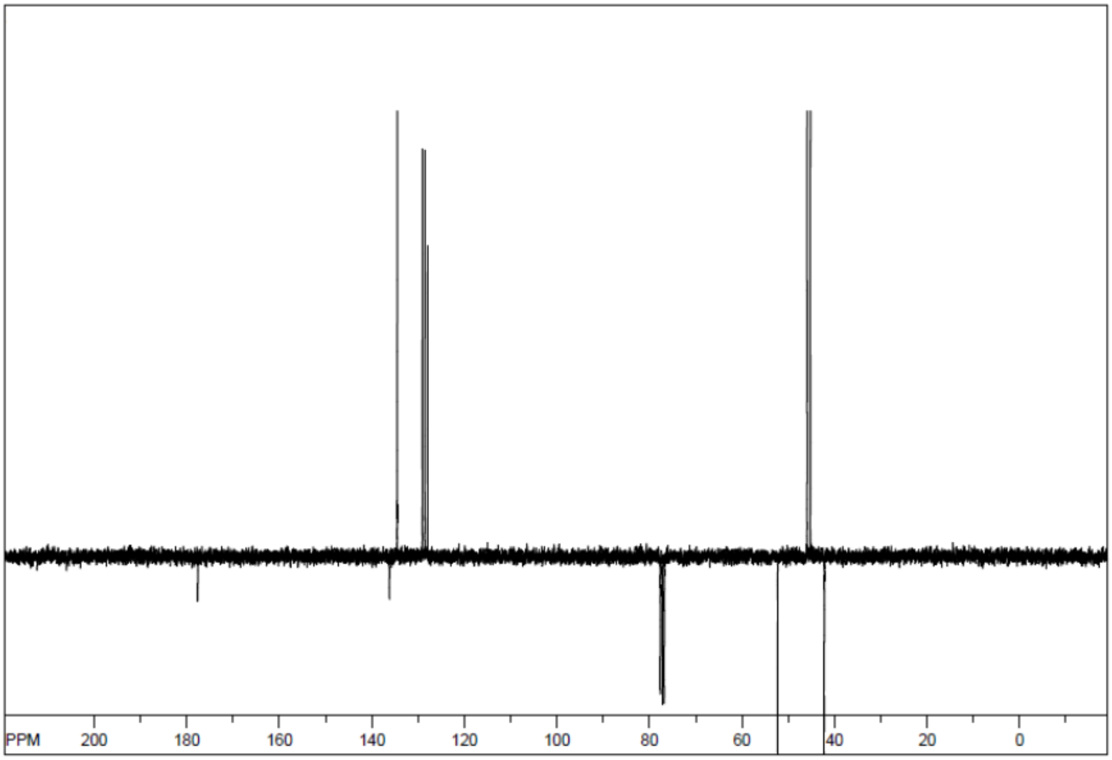

Figure S10. ^{13}C NMR spectrum of **7** in $CDCl_3$

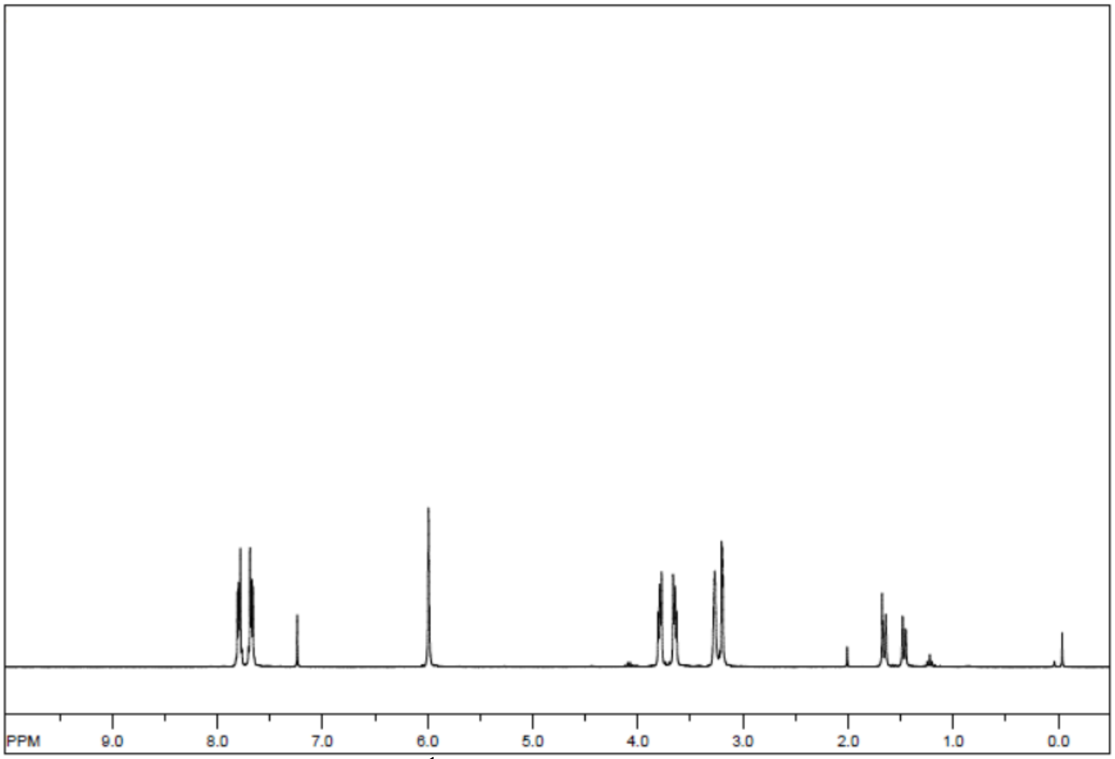

Figure S11. ^{1}H NMR spectrum of **8** in $CDCl_3$

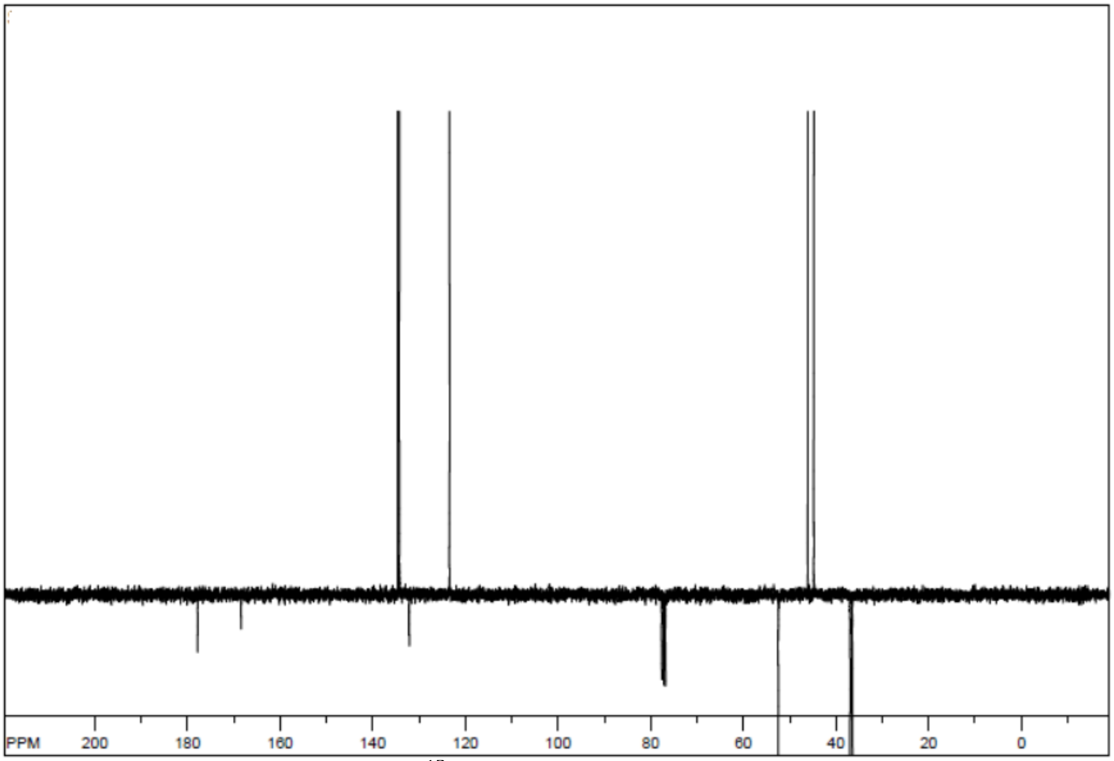

Figure S12. ^{13}C NMR spectrum of **8** in $CDCl_3$

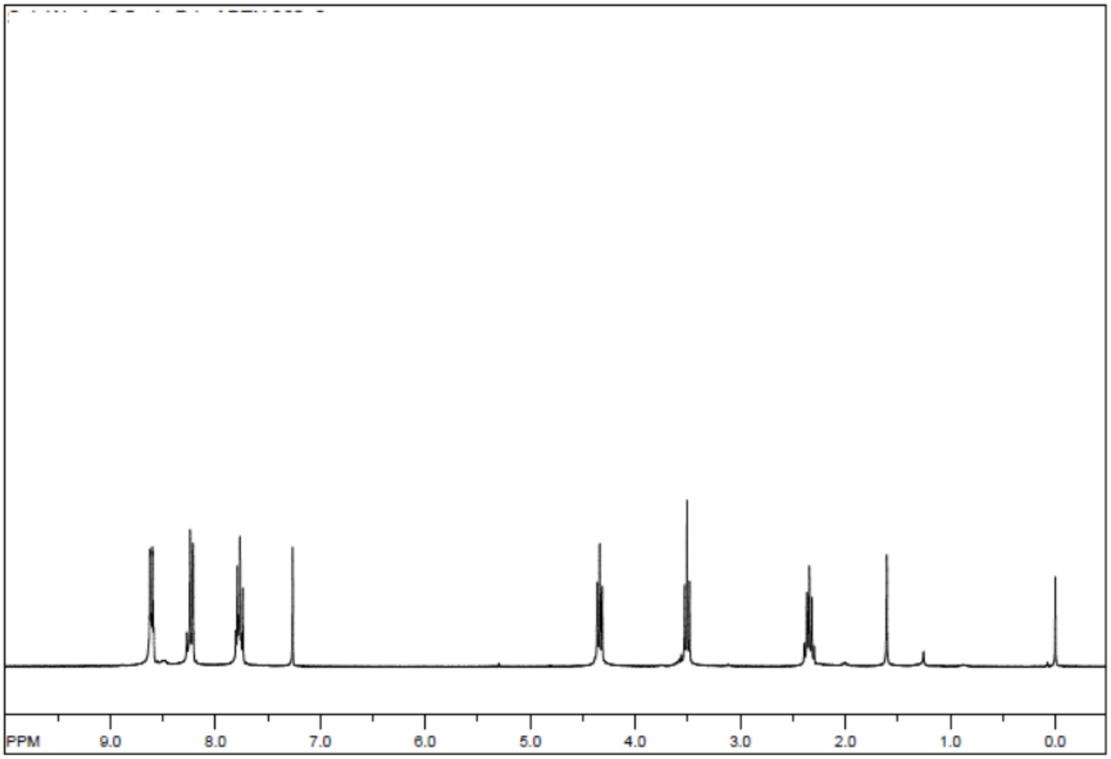

Figure S13. ^{1}H NMR spectrum of **18** in $CDCl_3$

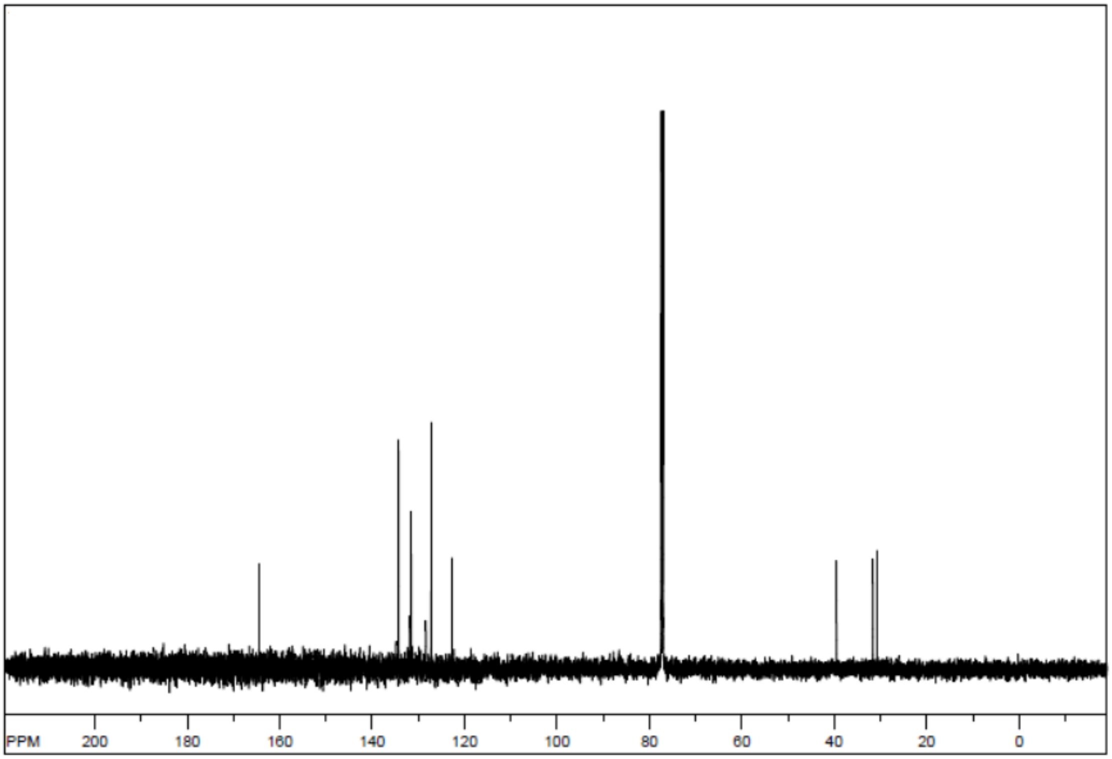

Figure S14. ^{13}C NMR spectrum of **18** in $CDCl_3$

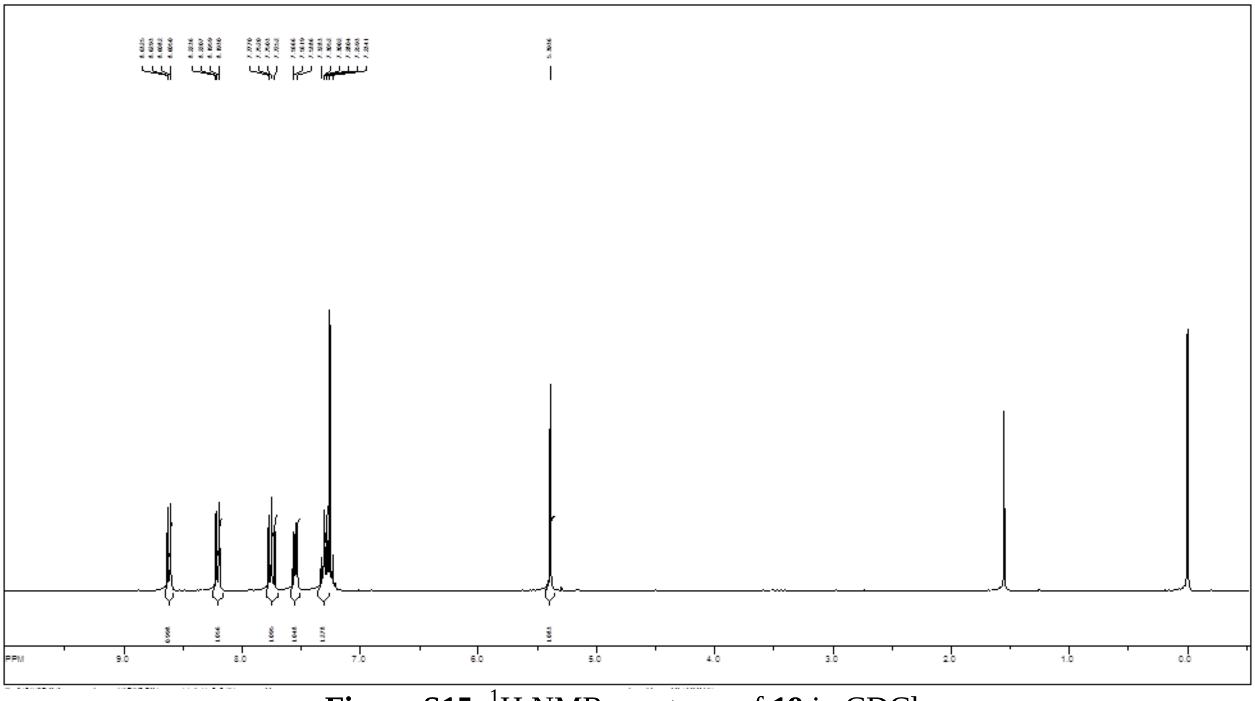

Figure S15. ^{1}H NMR spectrum of **19** in $CDCl_3$

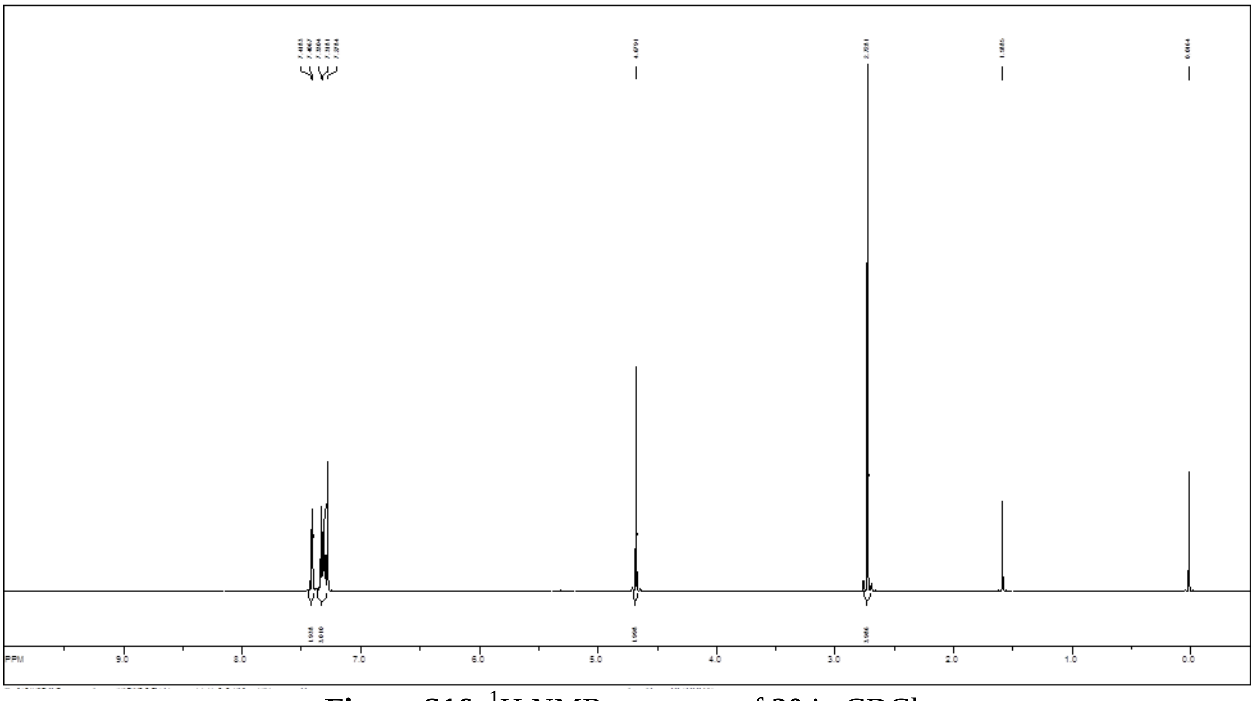

Figure S16. ^{1}H NMR spectrum of **20** in $CDCl_3$

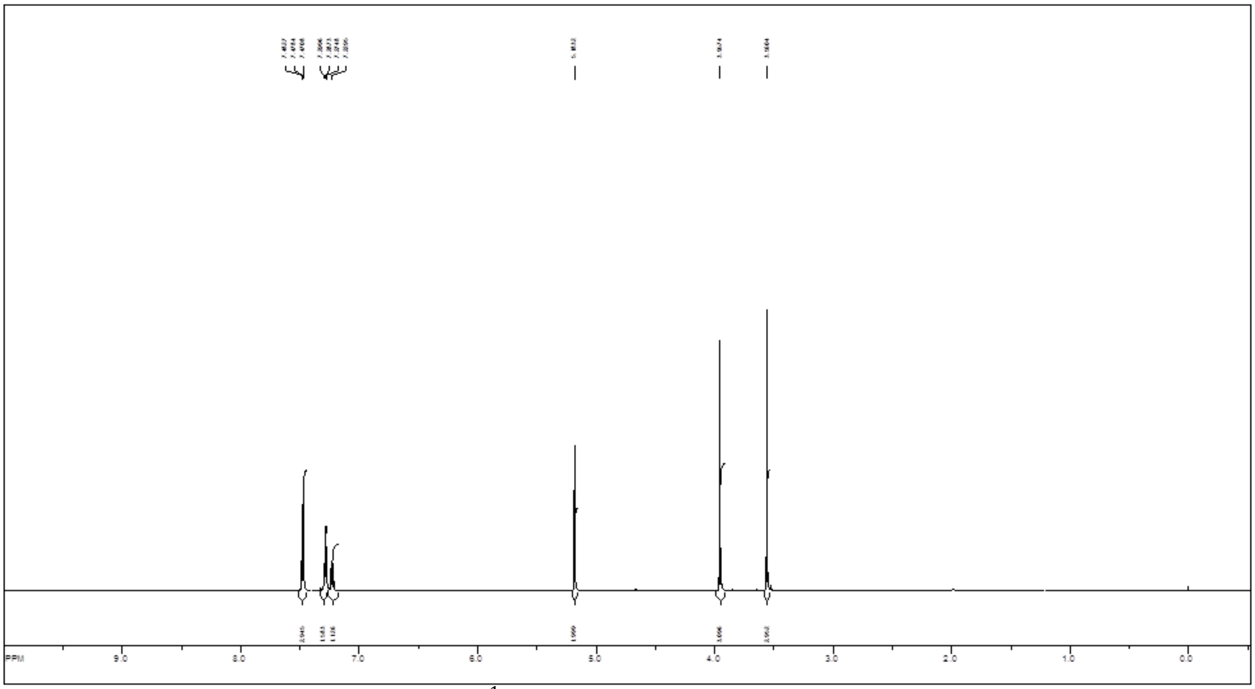

Figure S17. ^{1}H NMR spectrum of **21** in $CDCl_3$

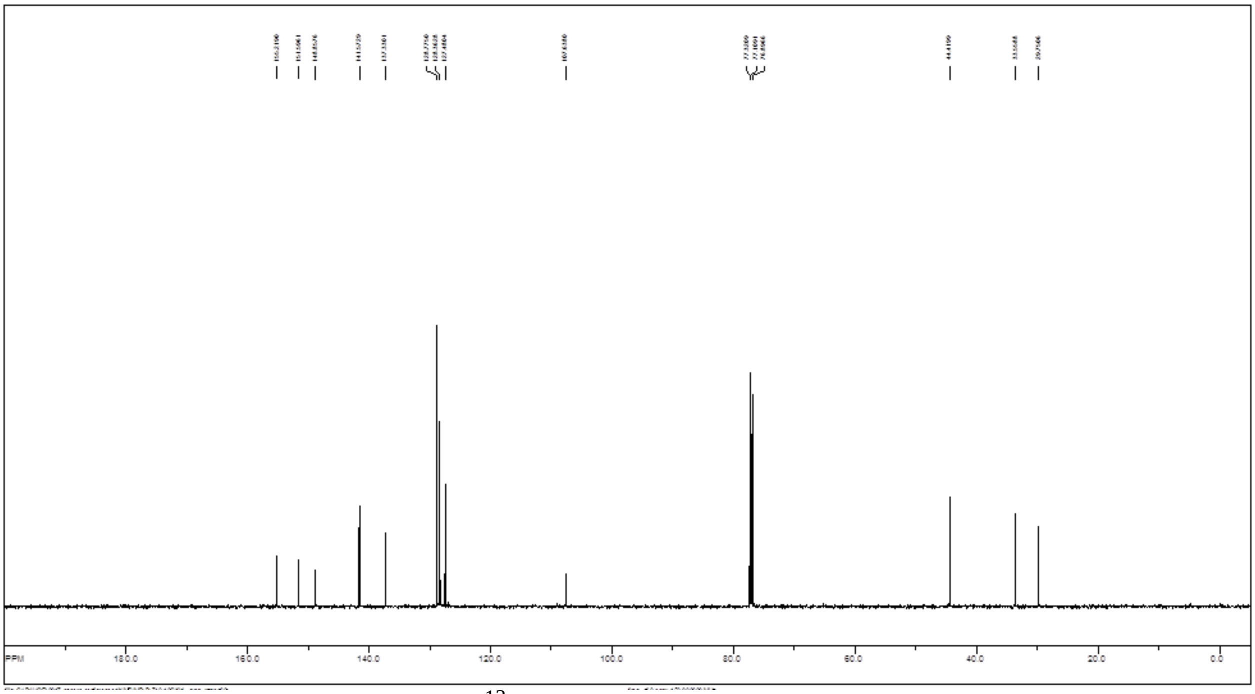

Figure S18. ^{13}C NMR spectrum of **21** in $CDCl_3$

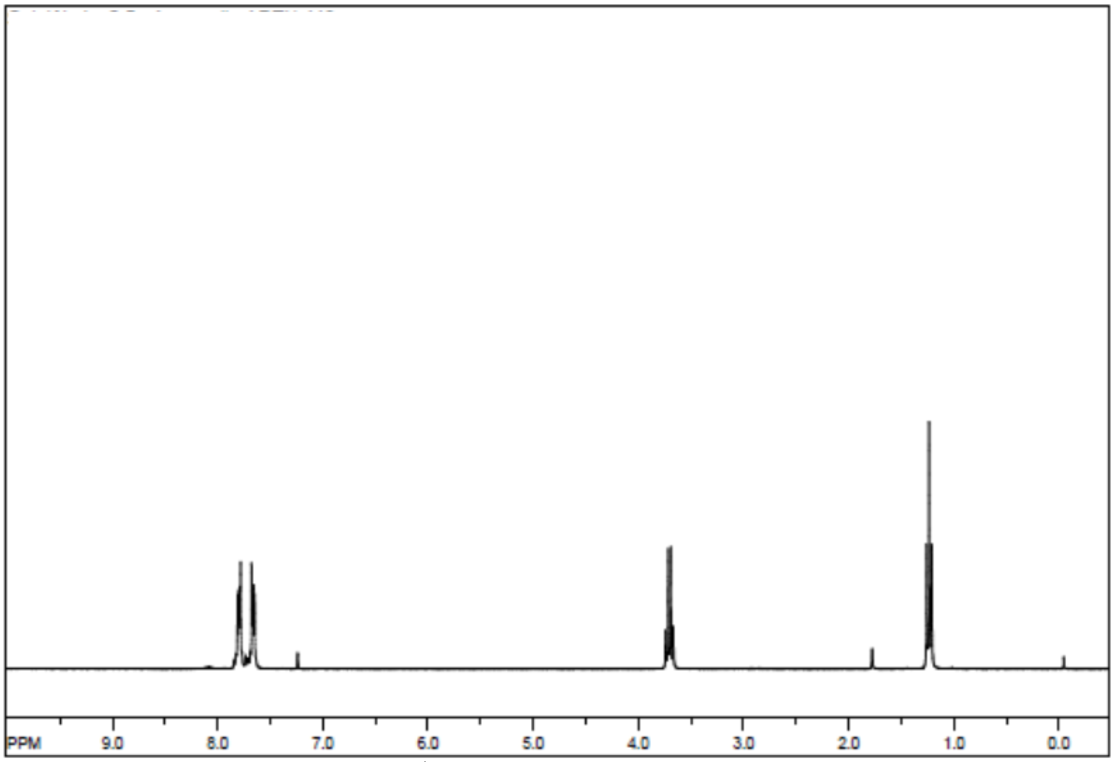

Figure S19. ^{1}H NMR spectrum of **22** in $CDCl_3$

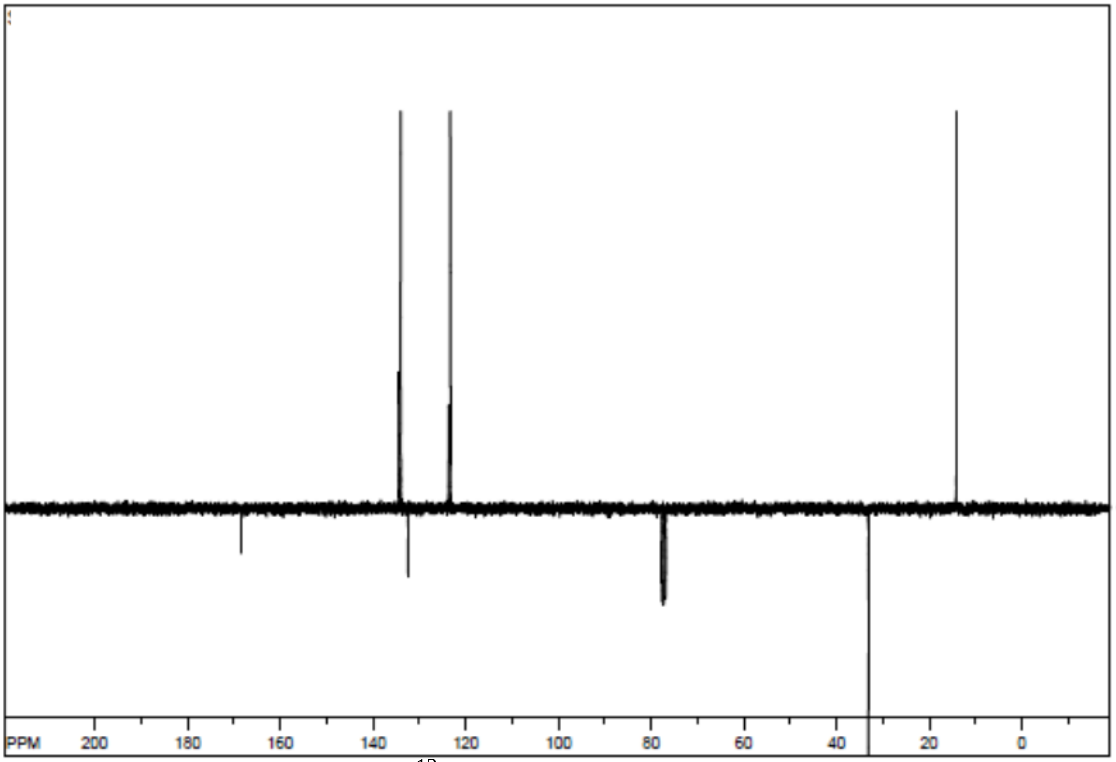

Figure S20. ^{13}C NMR spectrum of **22** in $CDCl_3$

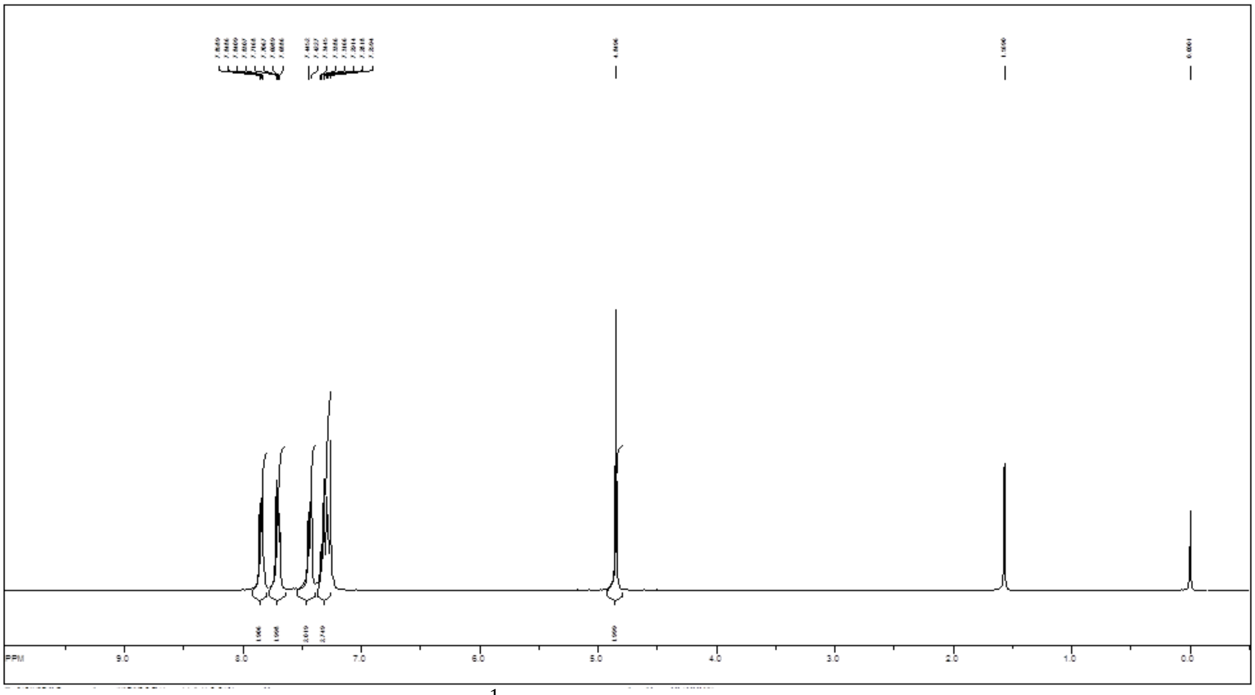

Figure S21. ^{1}H NMR spectrum of **23** in $CDCl_3$

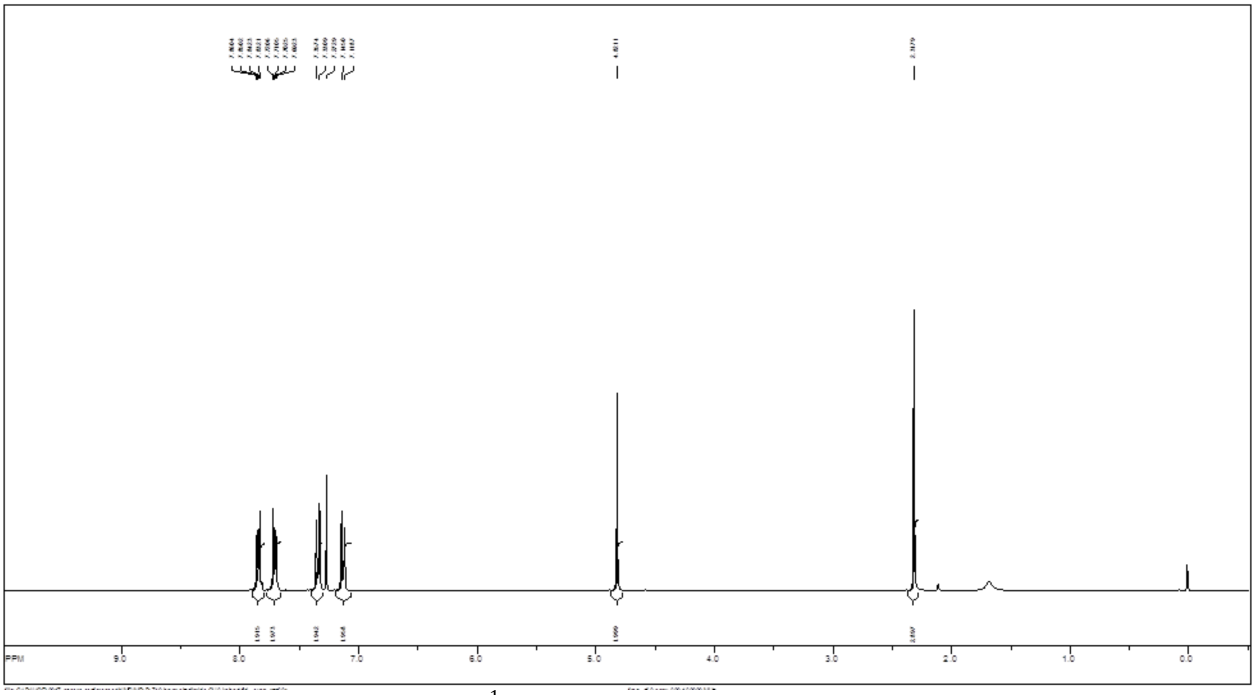

Figure S22. ^{1}H NMR spectrum of **24** in $CDCl_3$

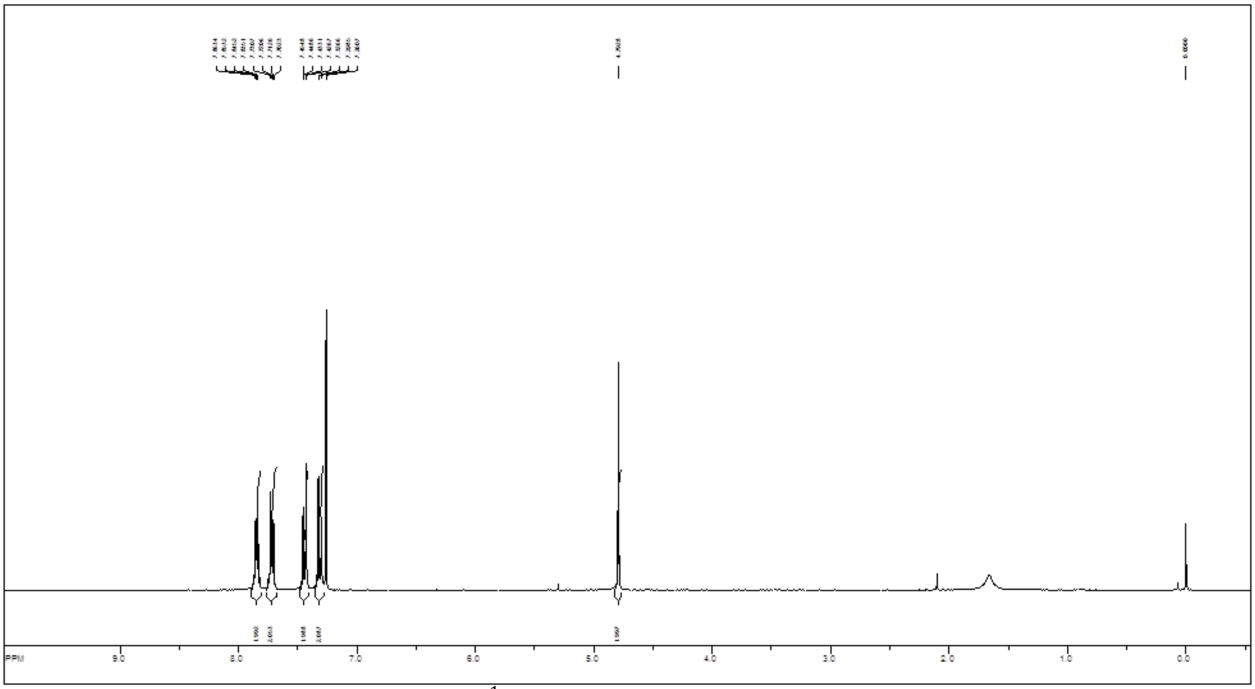

Figure S23. ^{1}H NMR spectrum of **25** in $CDCl_3$

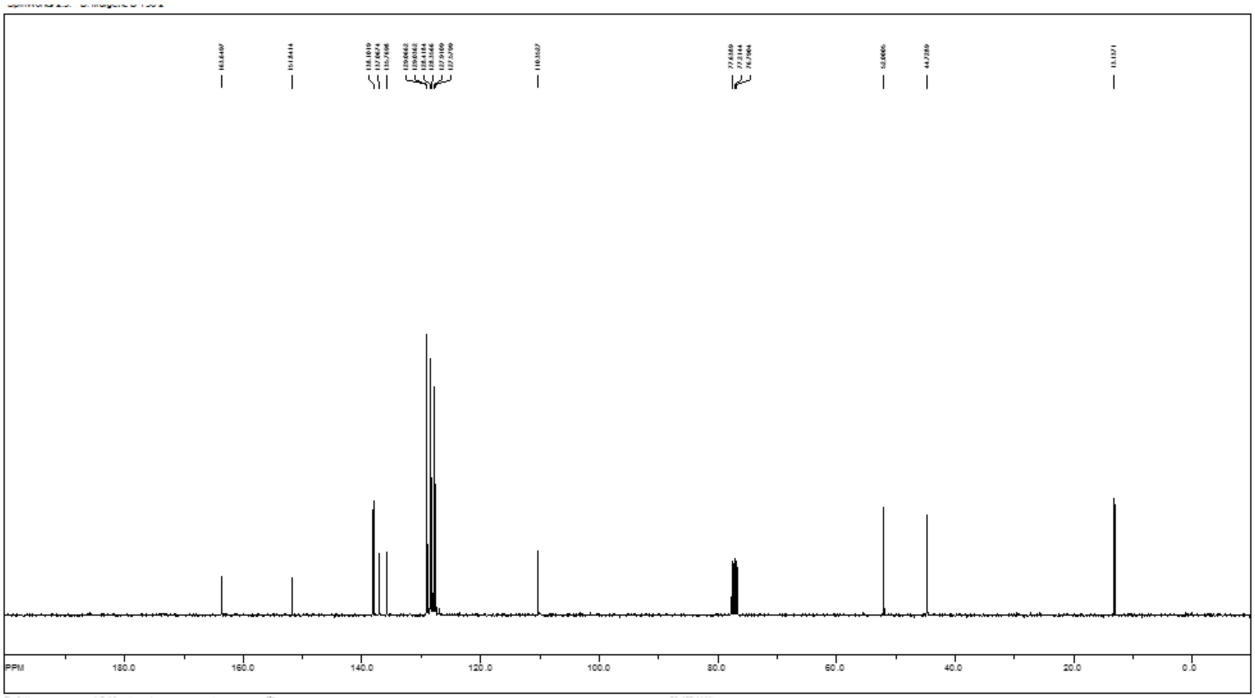

Figure S24. ^{13}C NMR spectrum of **25** in $CDCl_3$

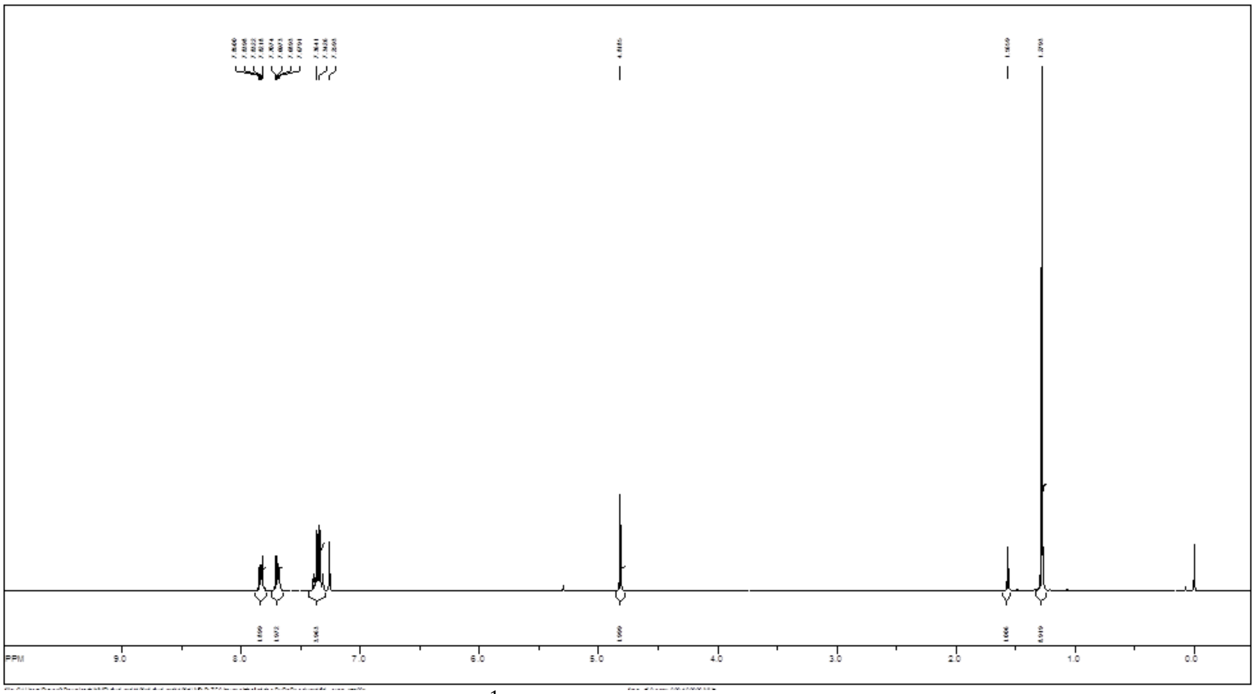

Figure S25. ^{1}H NMR spectrum of **26** in $CDCl_3$

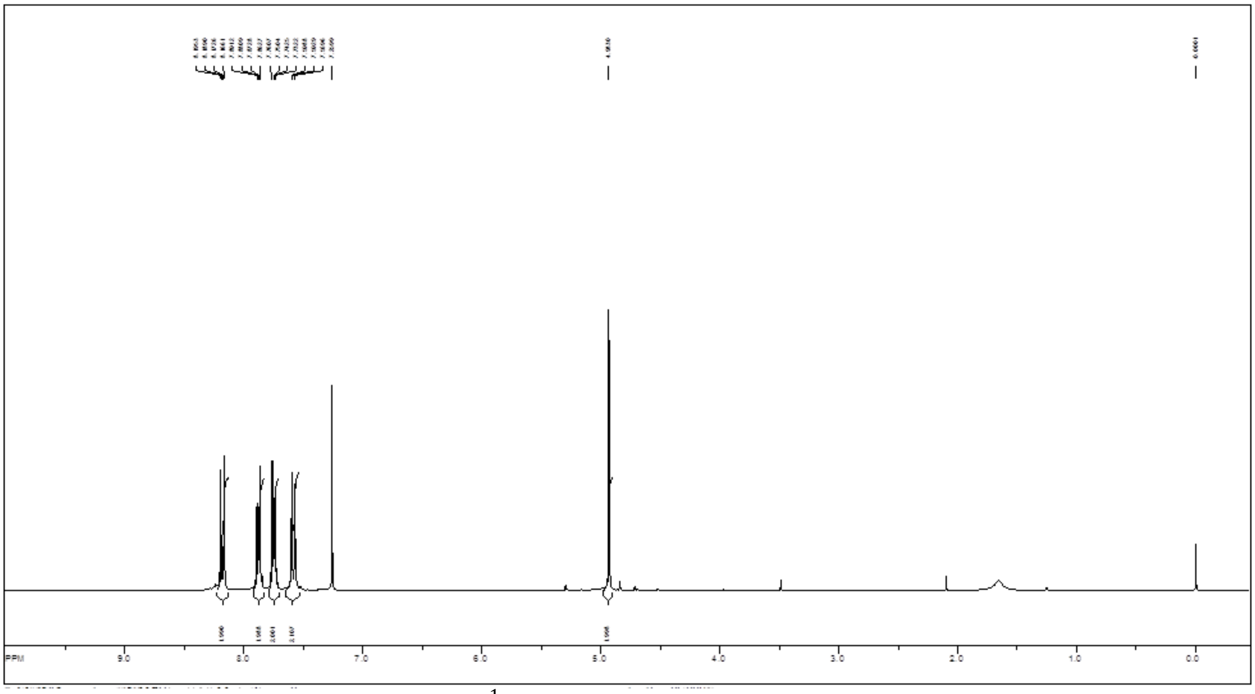

Figure S26. ^{1}H NMR spectrum of **27** in $CDCl_3$

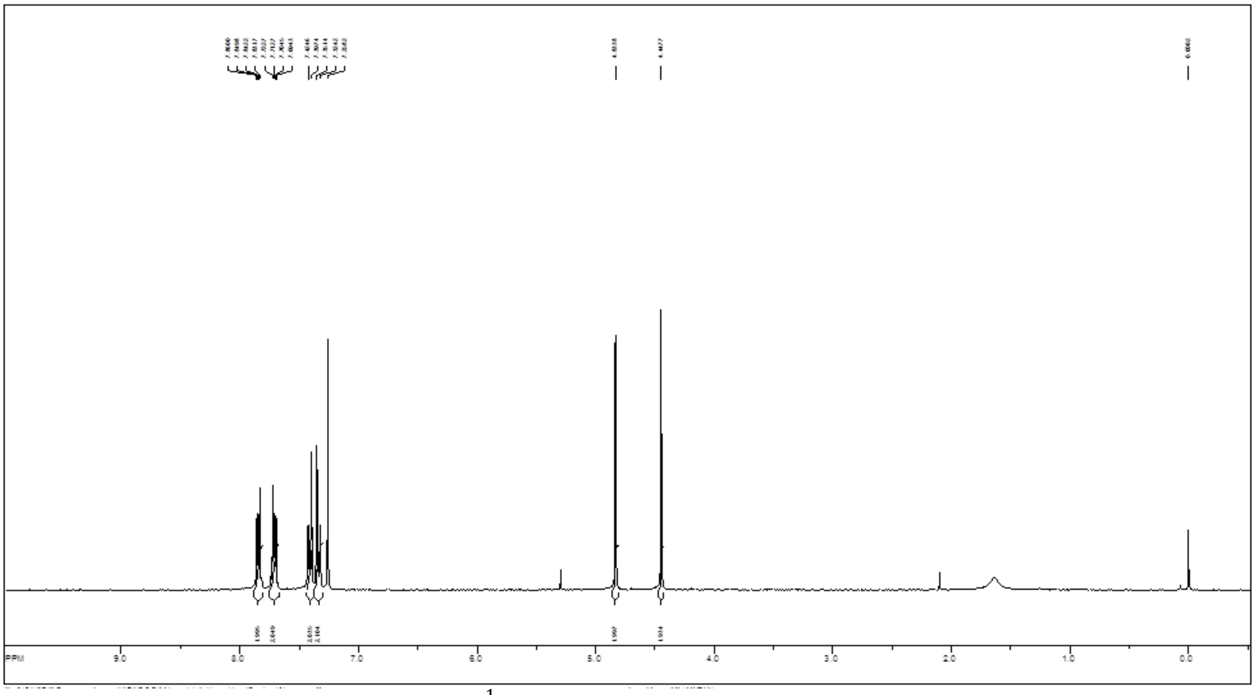

Figure S27. ^{1}H NMR spectrum of **28** in $CDCl_3$

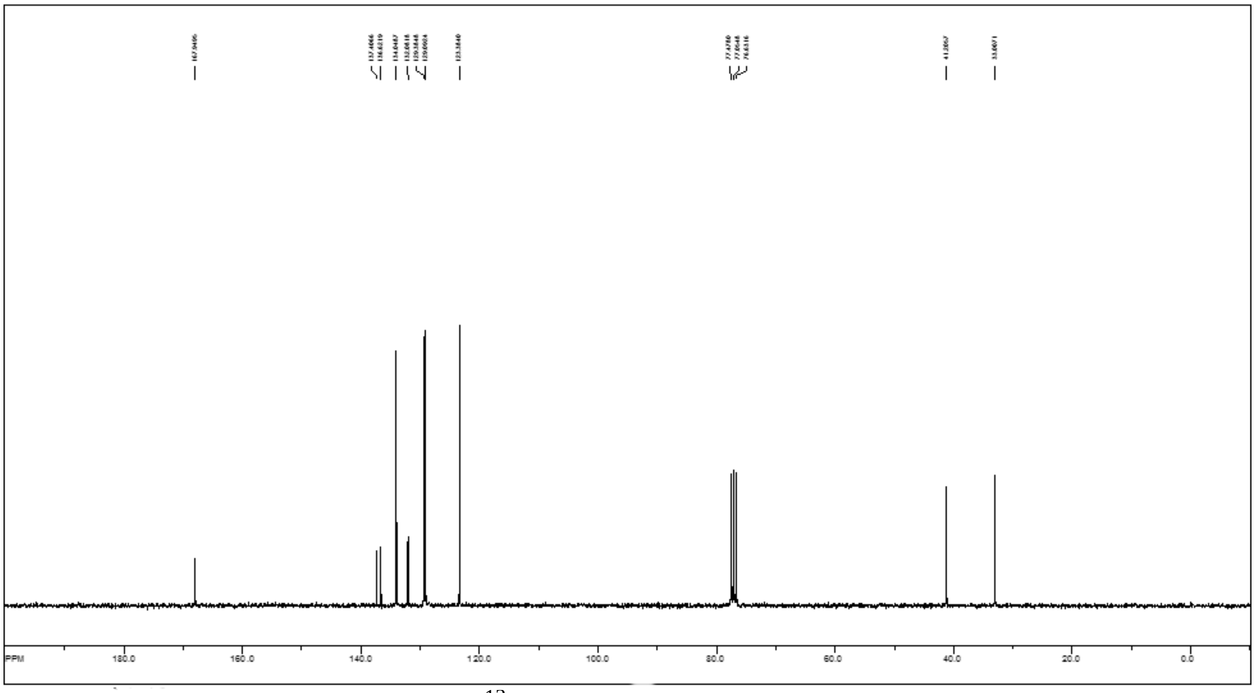

Figure S28. ^{13}C NMR spectrum of **28** in $CDCl_3$

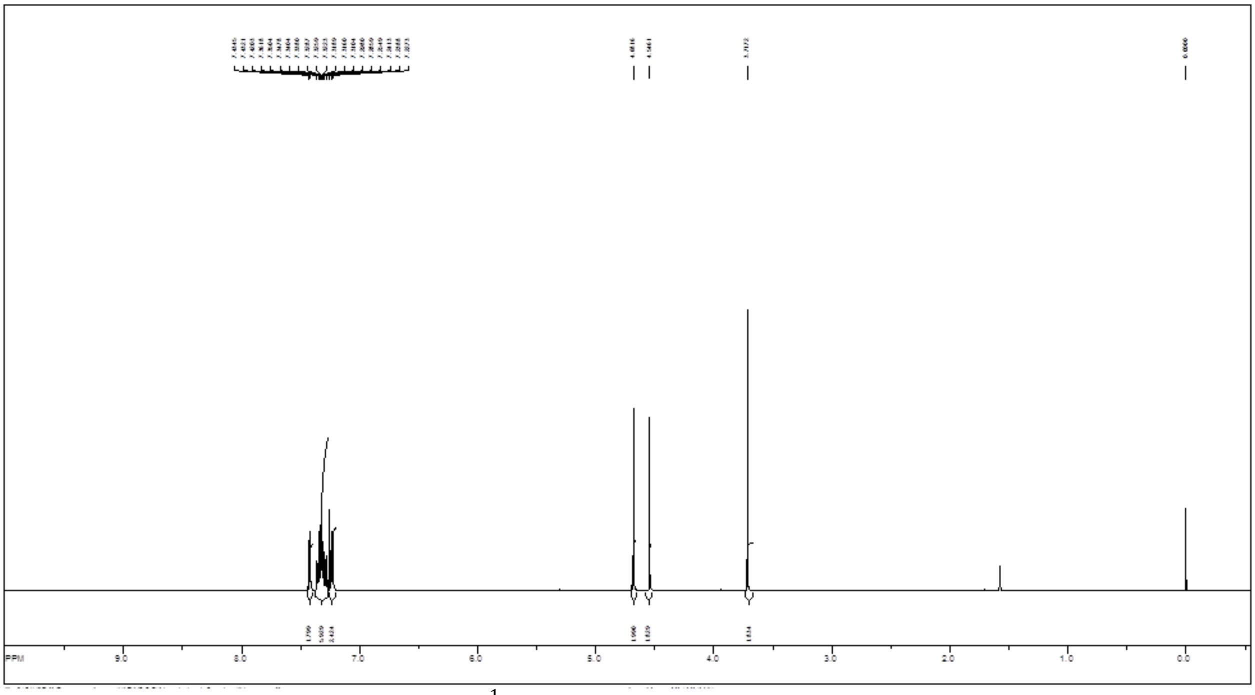

Figure S29. ^{1}H NMR spectrum of **30** in $CDCl_3$

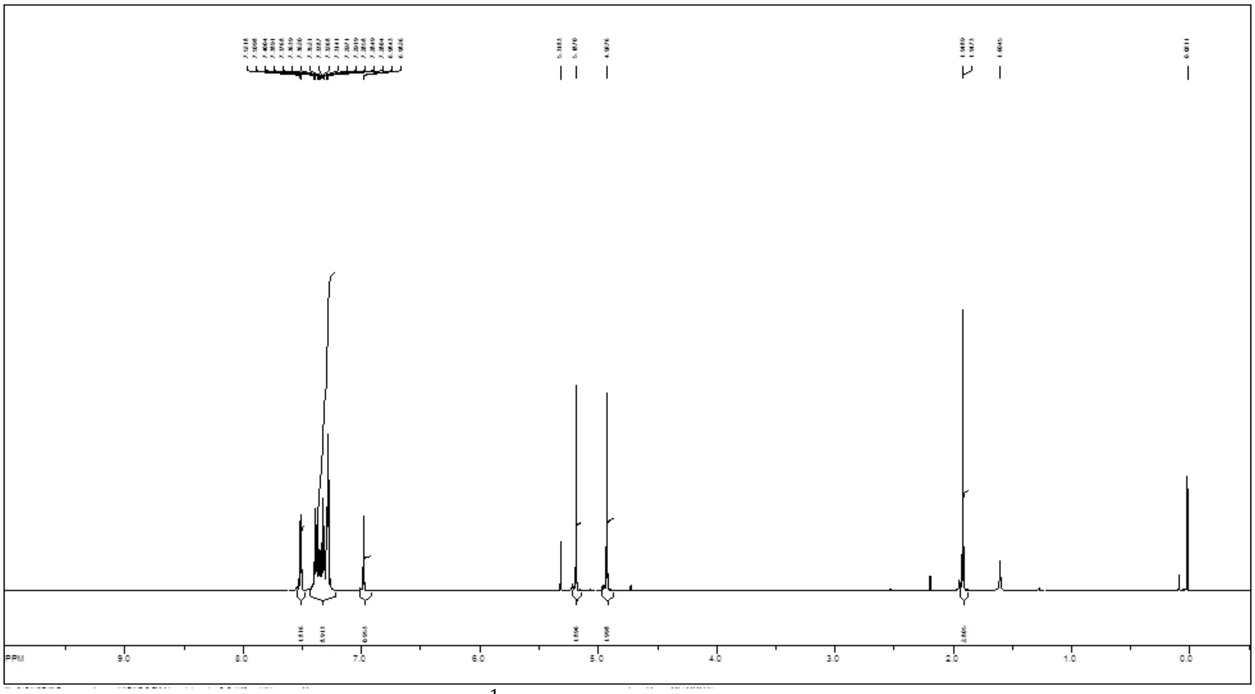

Figure S30. ^{1}H NMR spectrum of **31** in $CDCl_3$

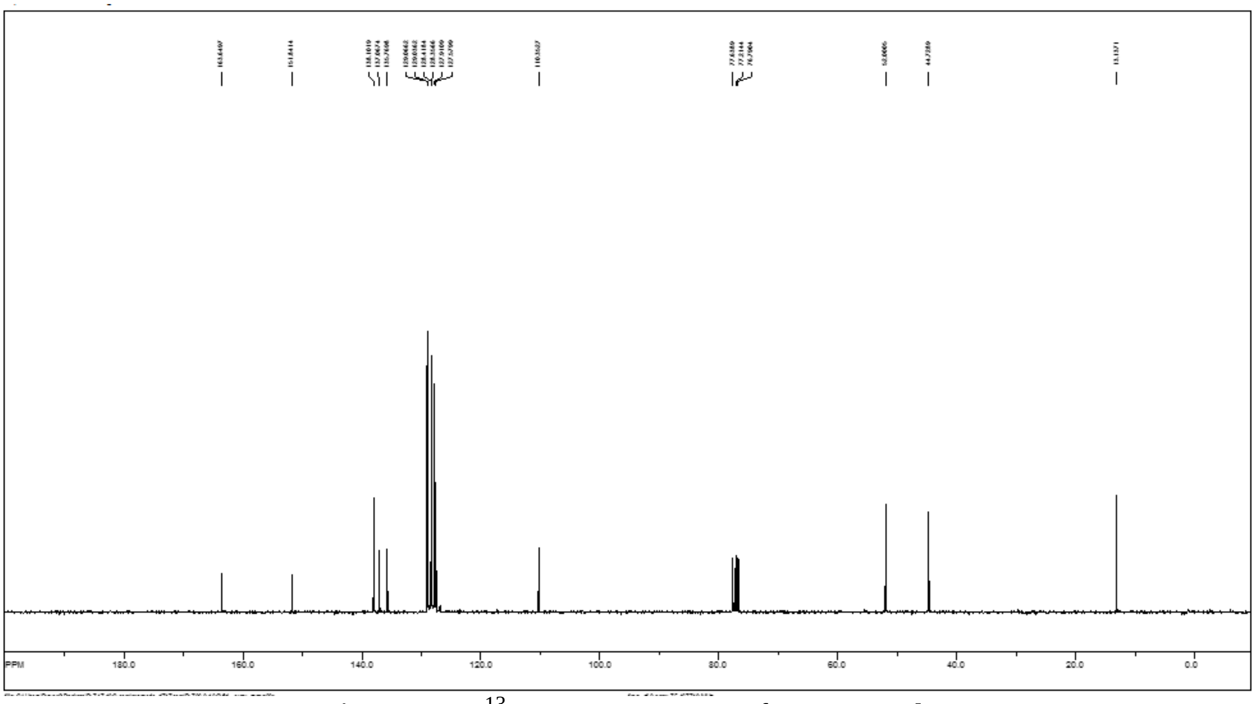

Figure S31. ^{13}C NMR spectrum of **31** in $CDCl_3$

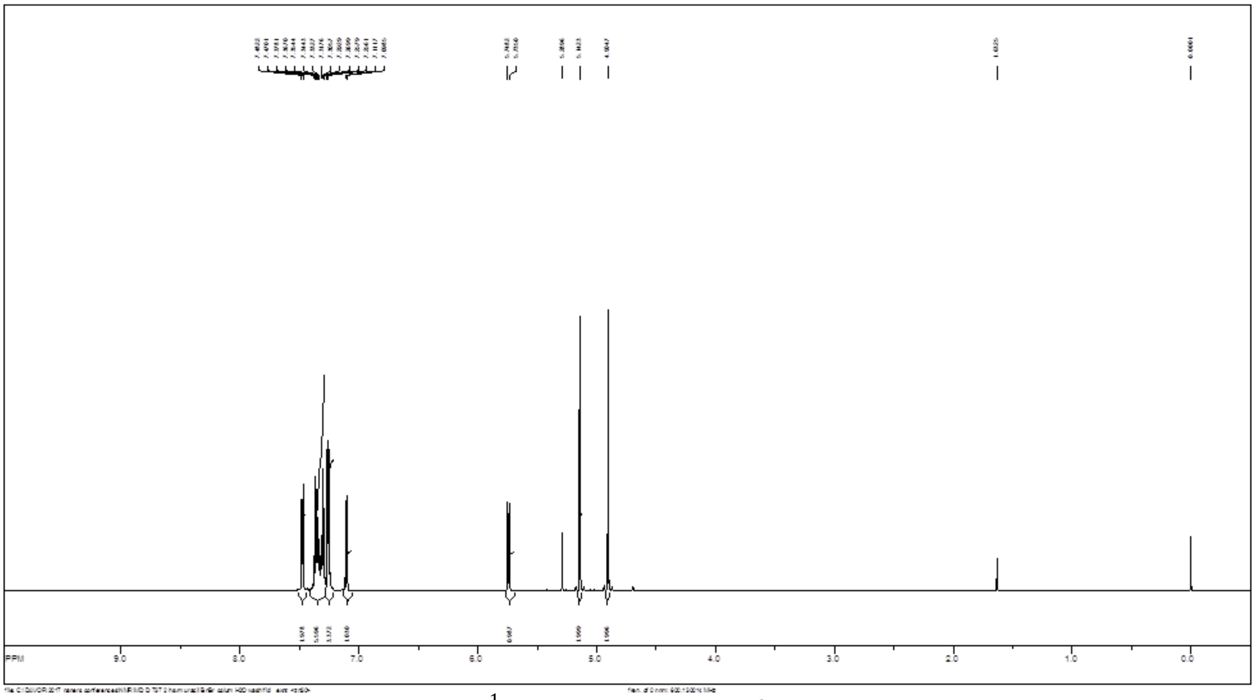

Figure S32. ^{1}H NMR spectrum of **32** in $CDCl_3$

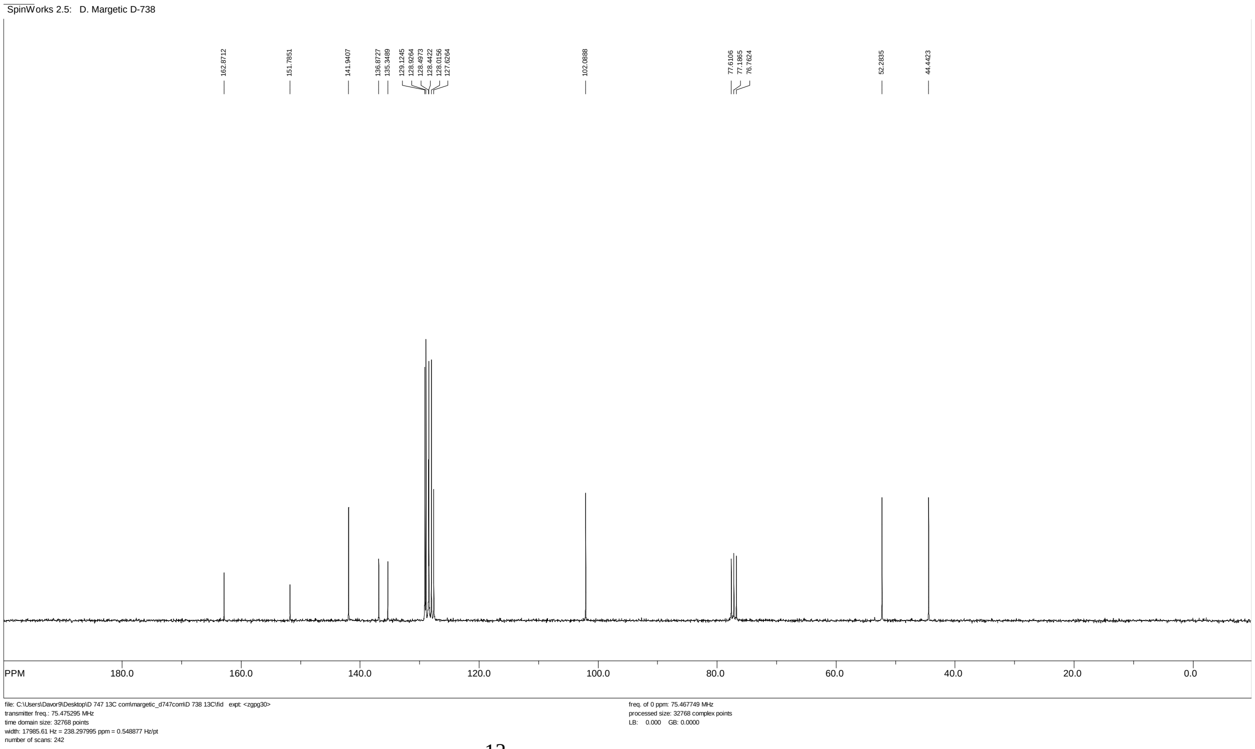

Figure S33. ^{13}C NMR spectrum of **32** in $CDCl_3$

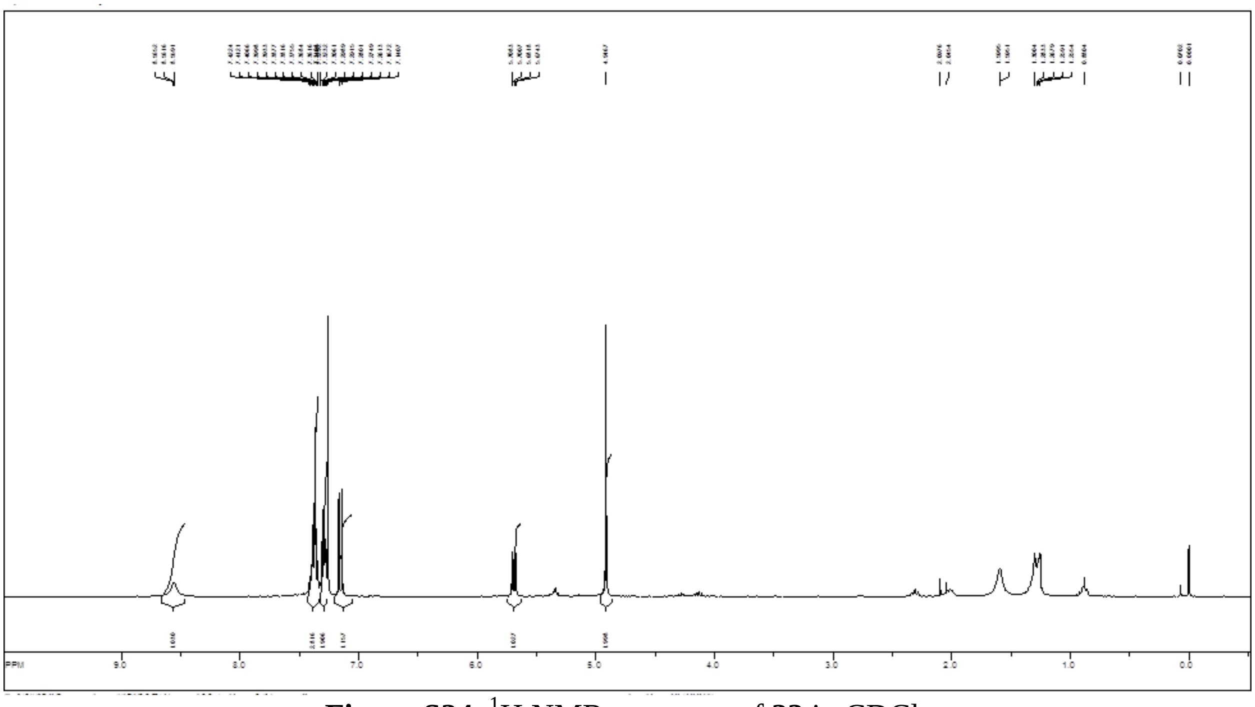

Figure S34. ^{1}H NMR spectrum of **33** in $CDCl_3$

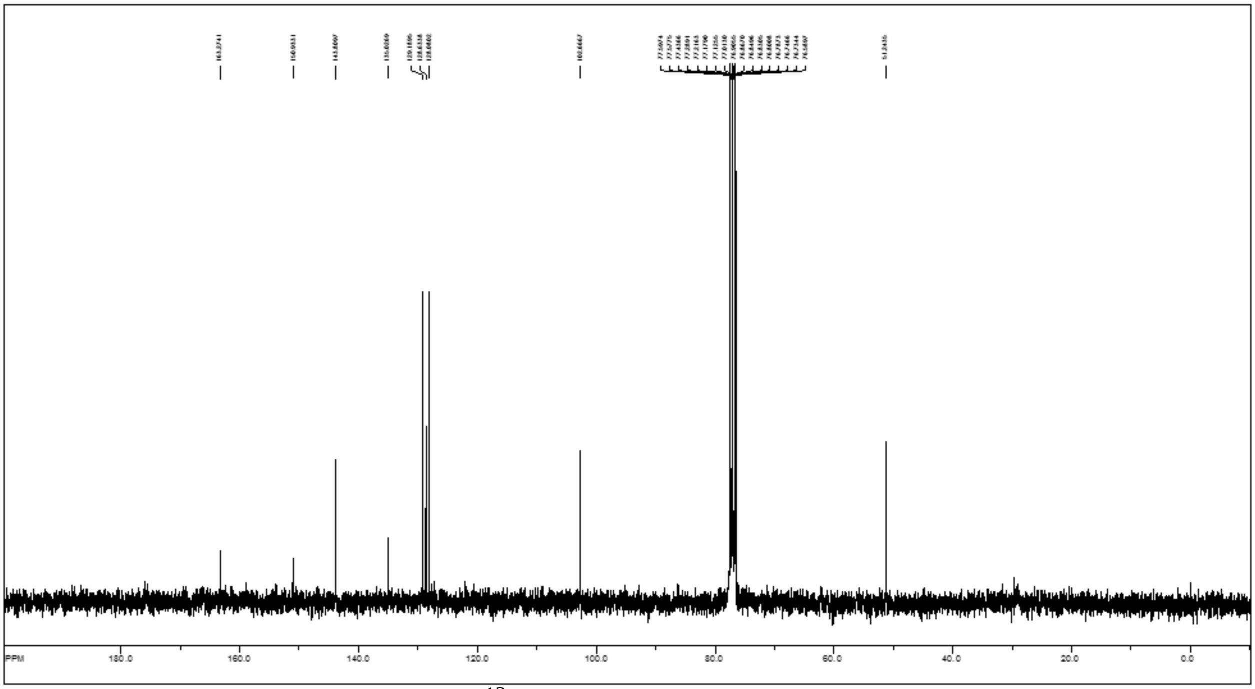

Figure S35. ^{13}C NMR spectrum of **33** in $CDCl_3$

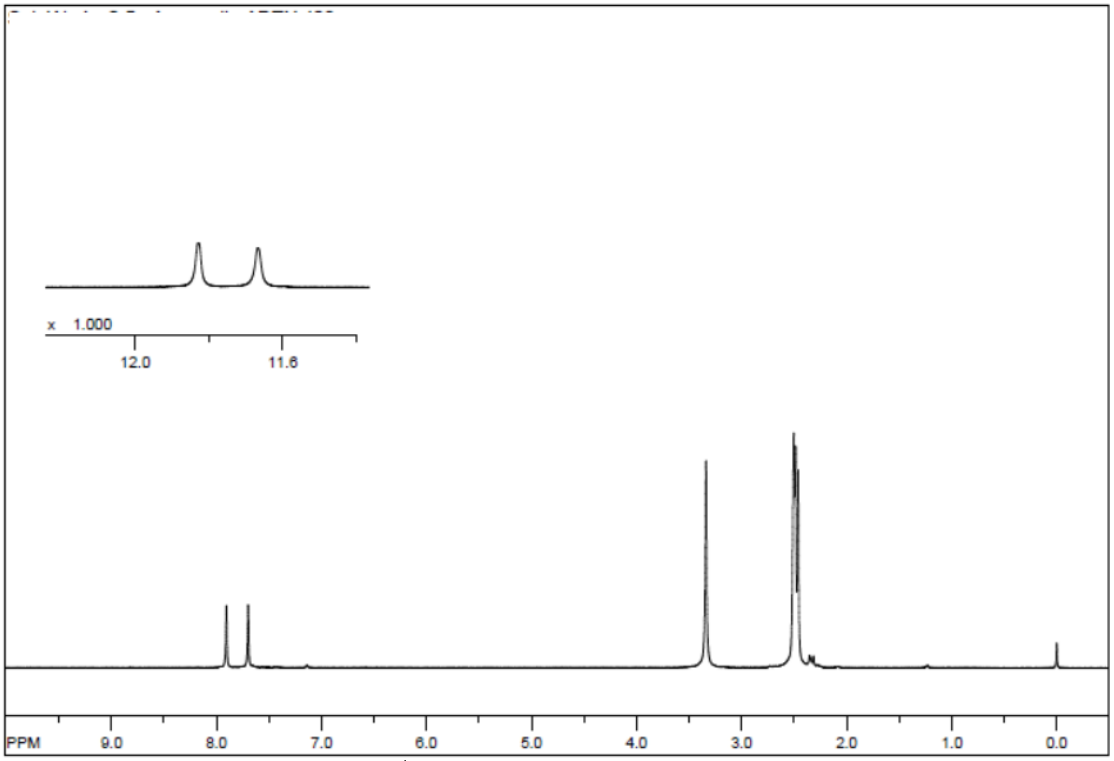

Figure S36. ^{1}H NMR spectrum of **36** in DMSO-d_6

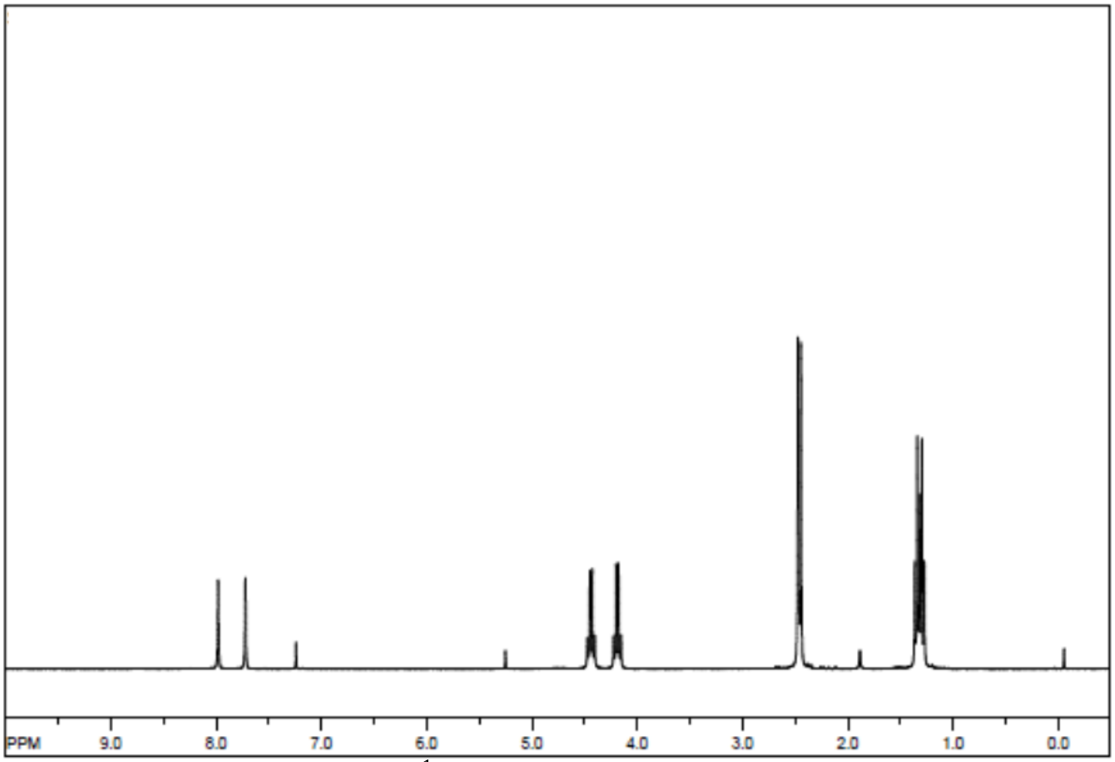

Figure S37. ^{1}H NMR spectrum of **37** in $CDCl_3$

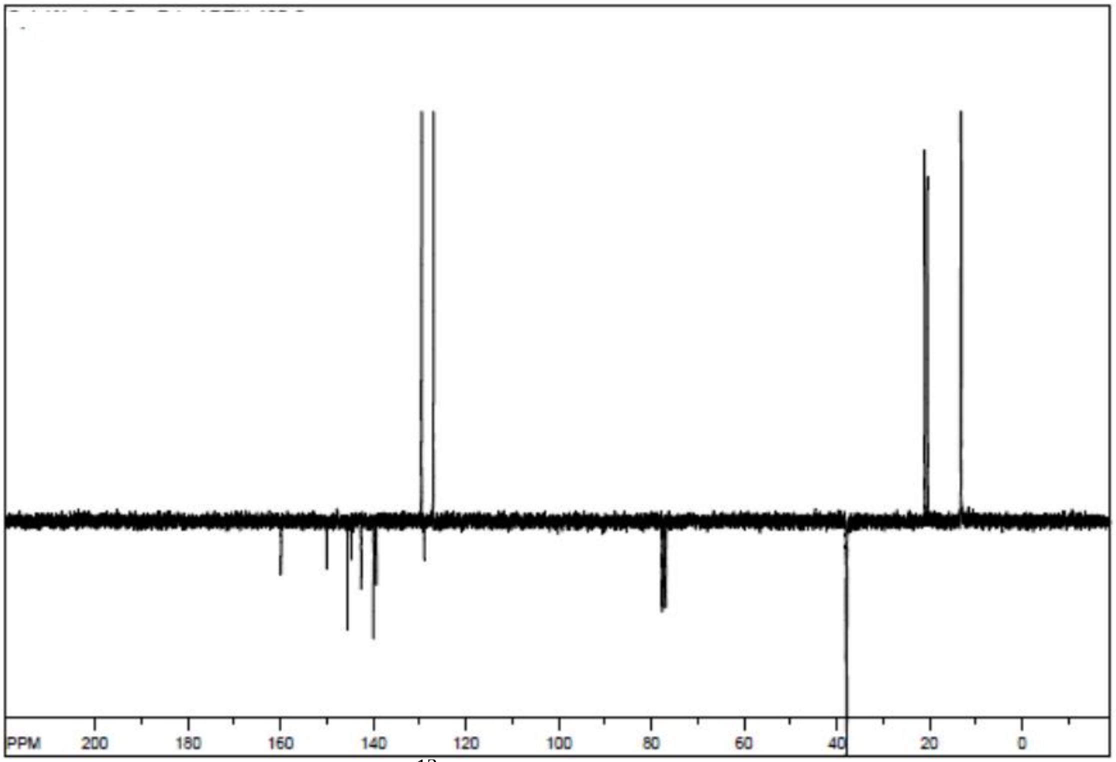

Figure S38. ^{13}C NMR spectrum of **37** in $CDCl_3$

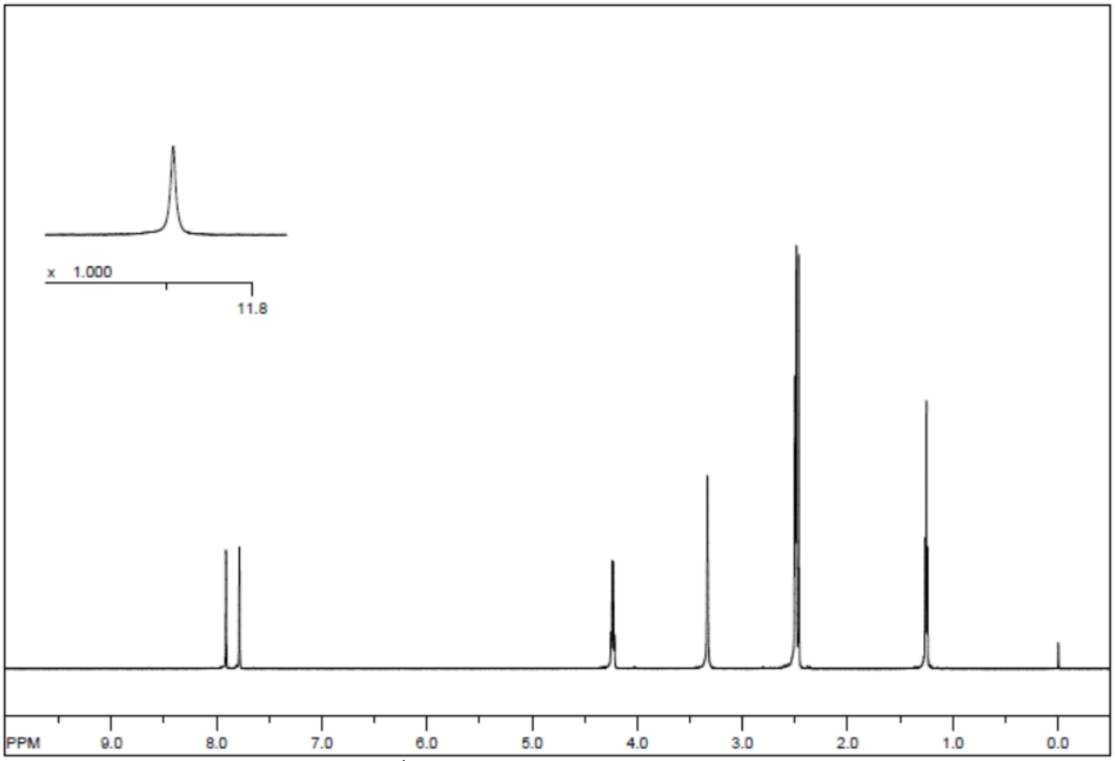

Figure S39. ^{1}H NMR spectrum of **38** in DMSO-d_6

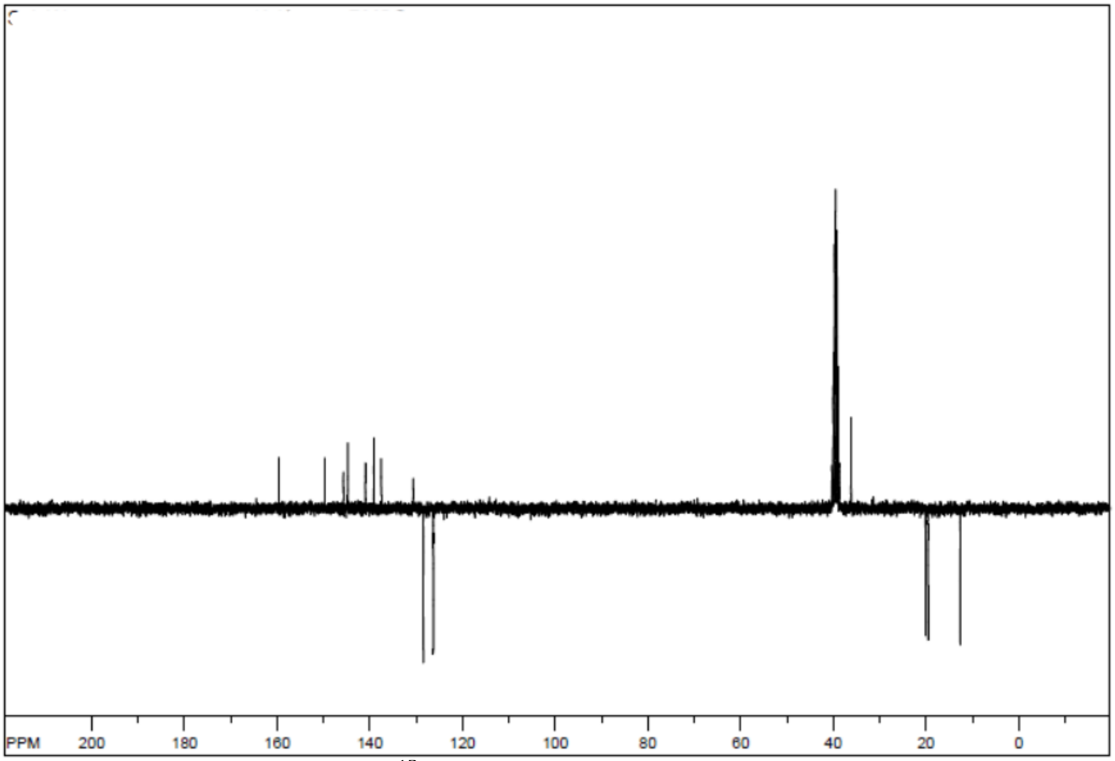

Figure S40. ^{13}C NMR spectrum of **38** in DMSO-d_6

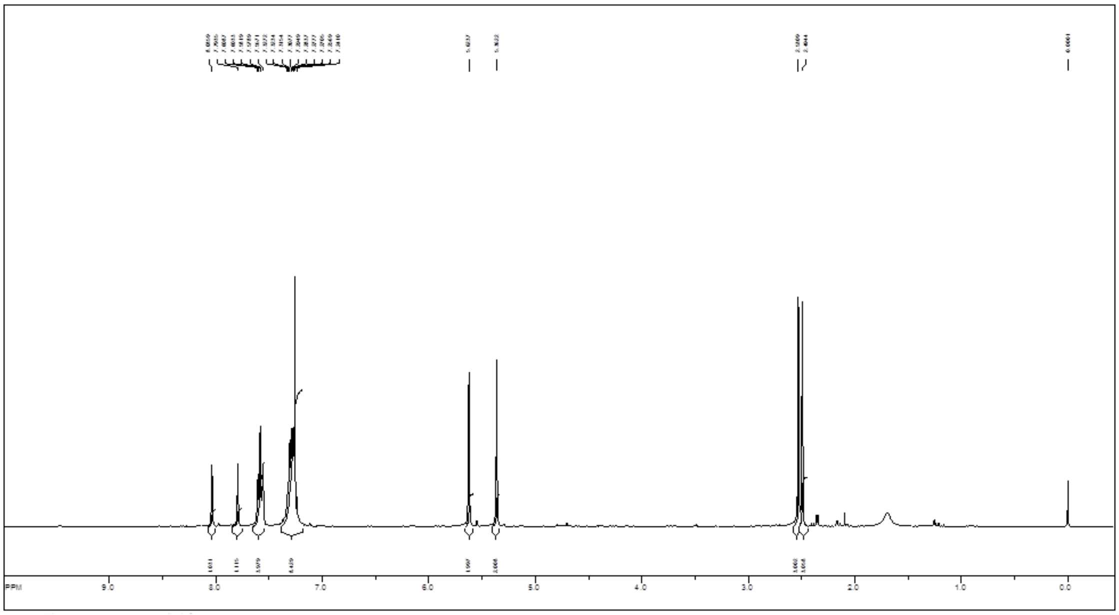

Figure S41. ^{1}H NMR spectrum **39** in $CDCl_3$

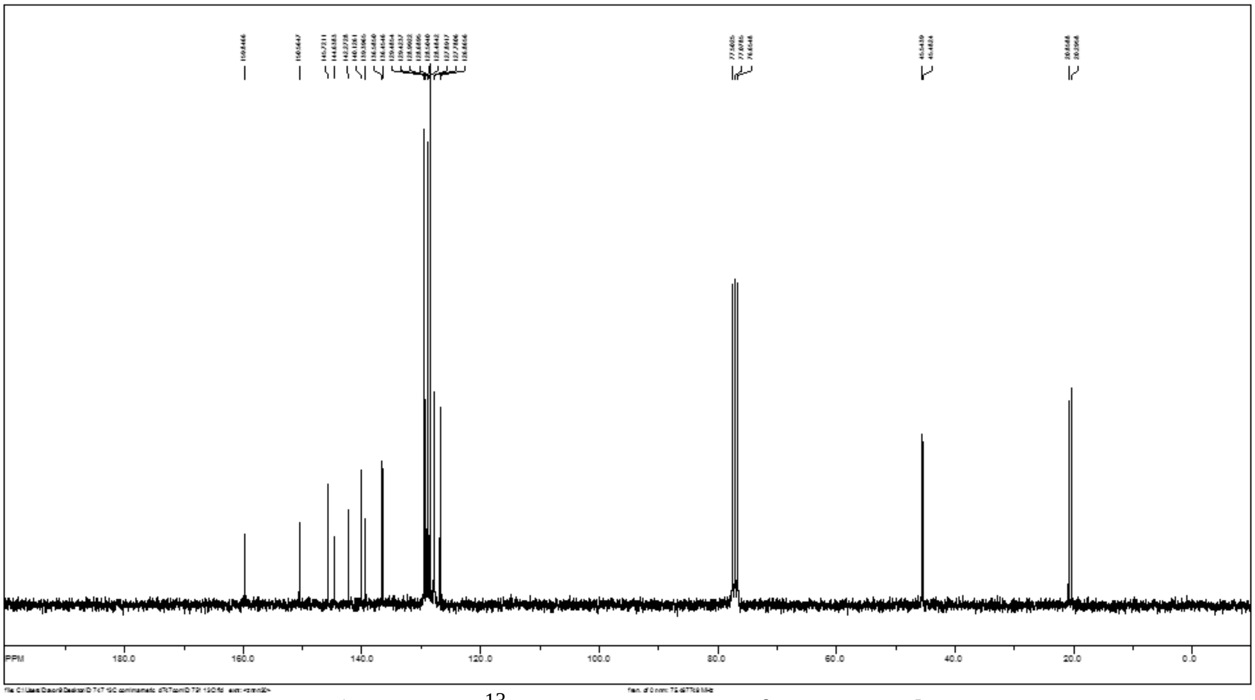

Figure S42. ^{13}C NMR spectrum of **39** in $CDCl_3$

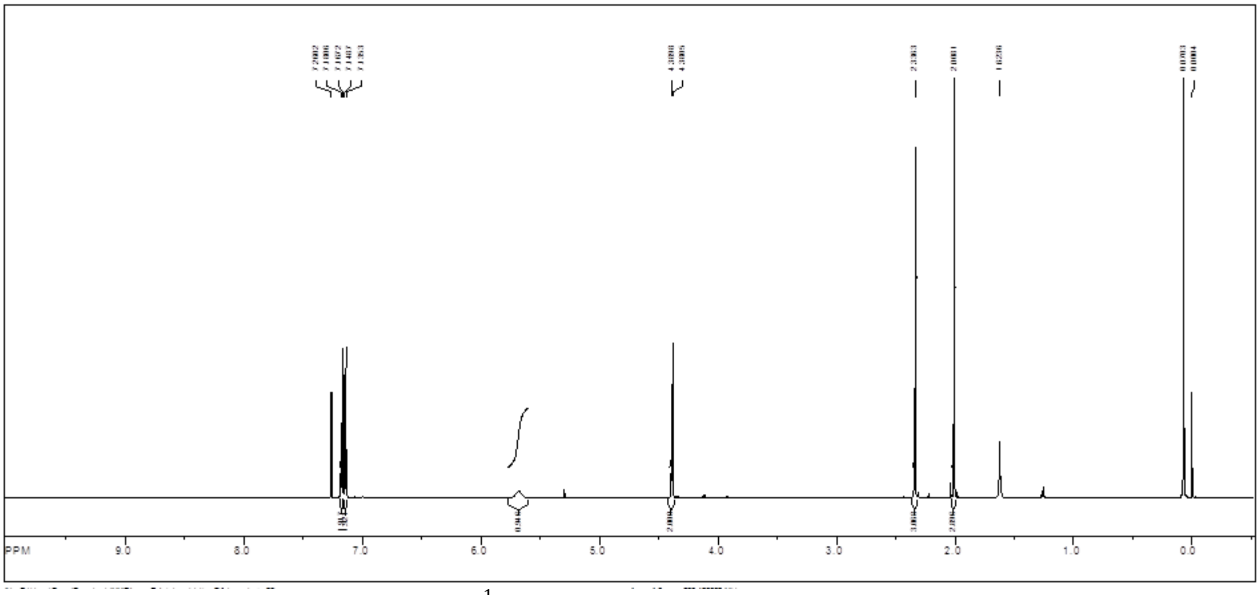

Figure S43. ^{1}H NMR spectrum of **42** in $CDCl_3$

IR SPECTRA

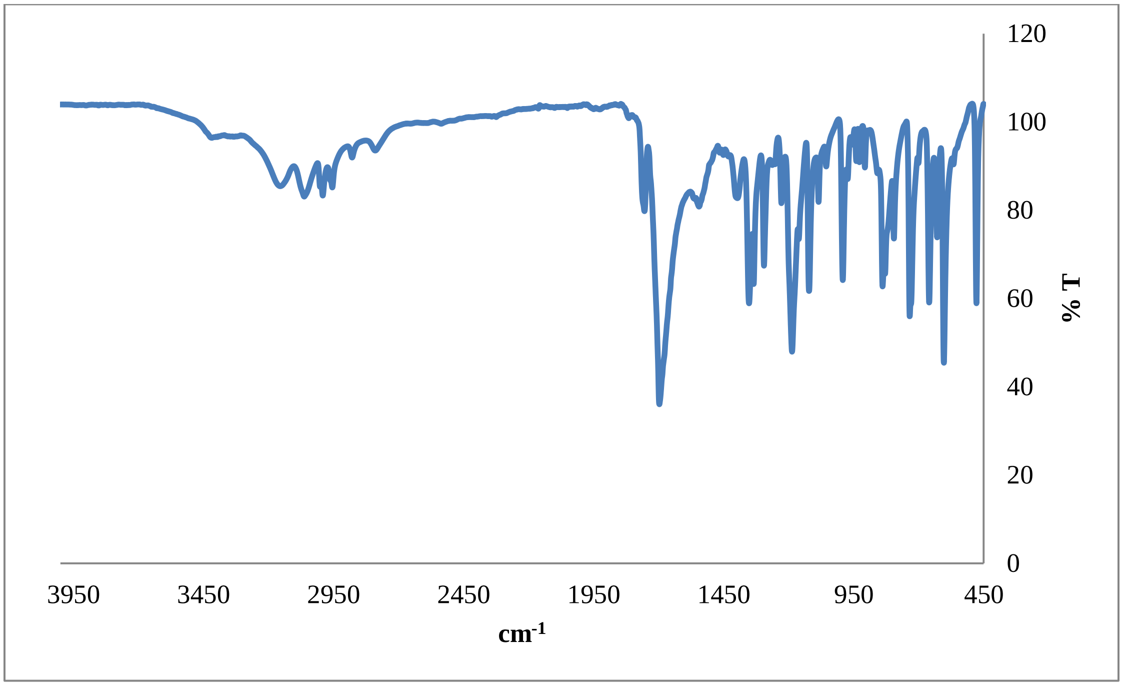

Figure S44. IR spectrum of **1**

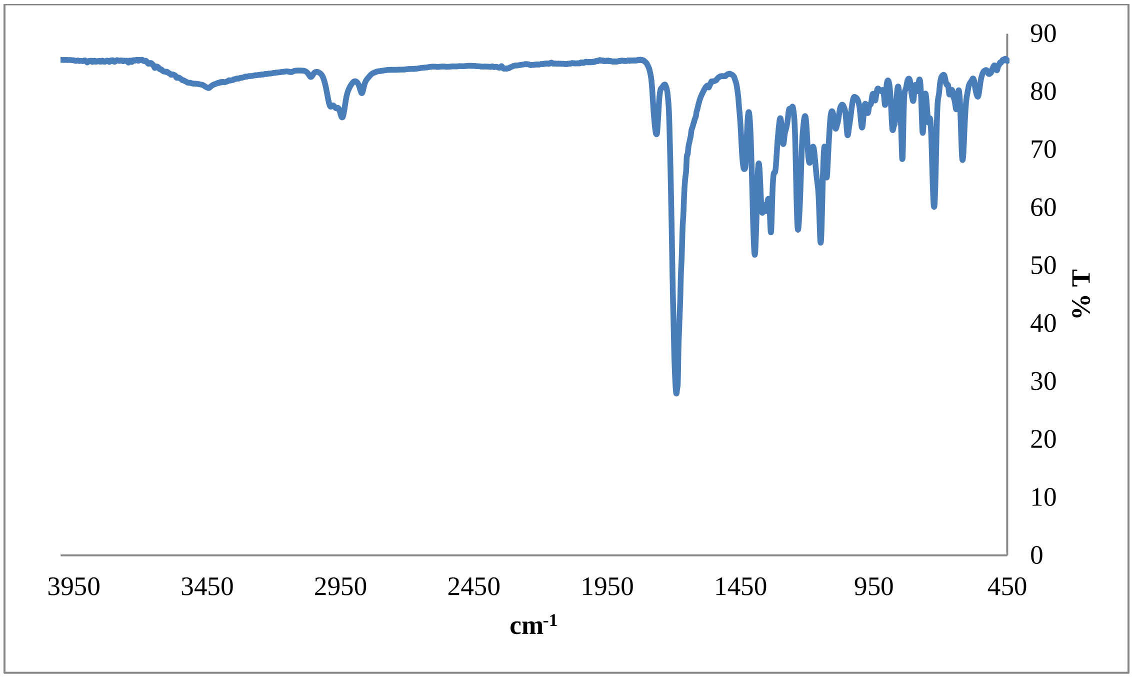

Figure S45. IR spectrum of **3**

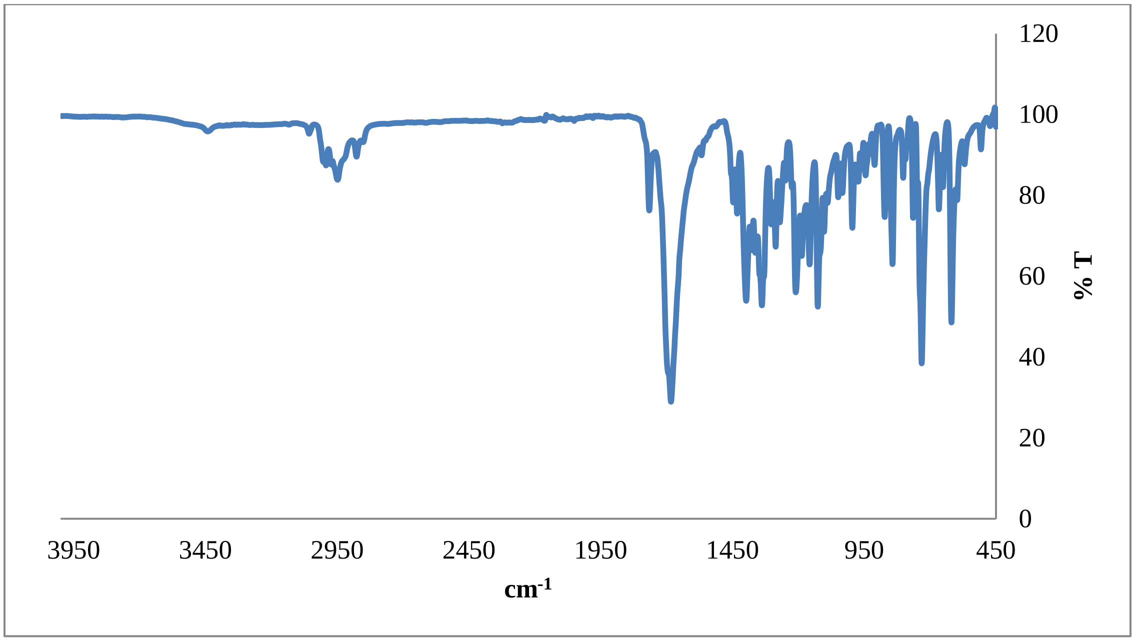

Figure S46. IR spectrum of **4**

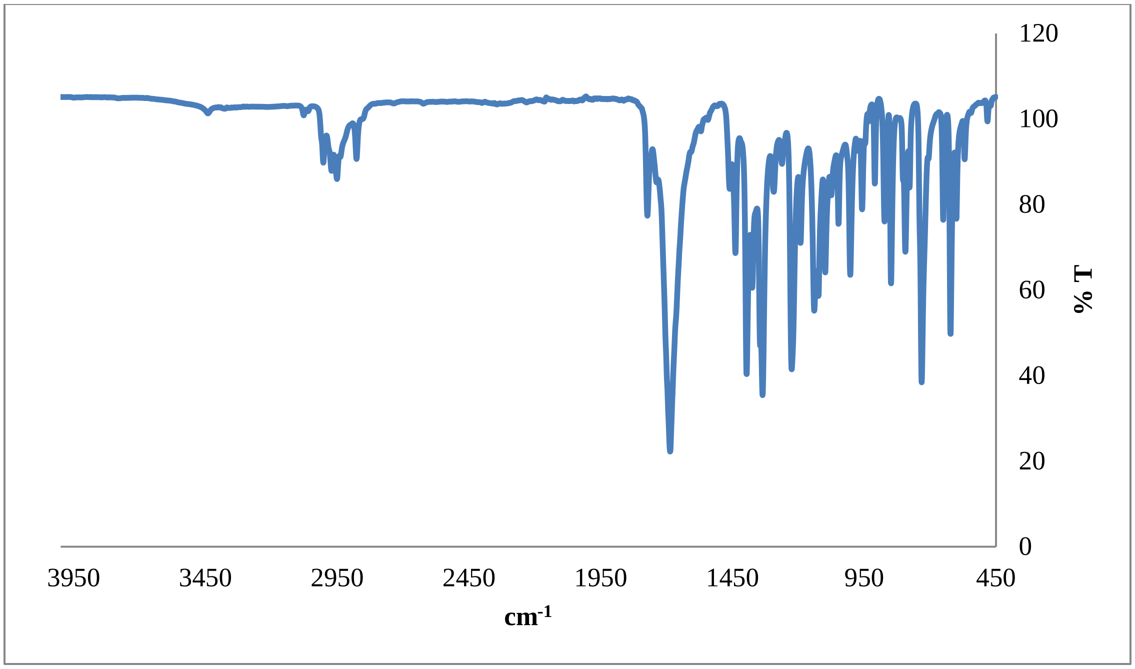

Figure S47. IR spectrum of **5**

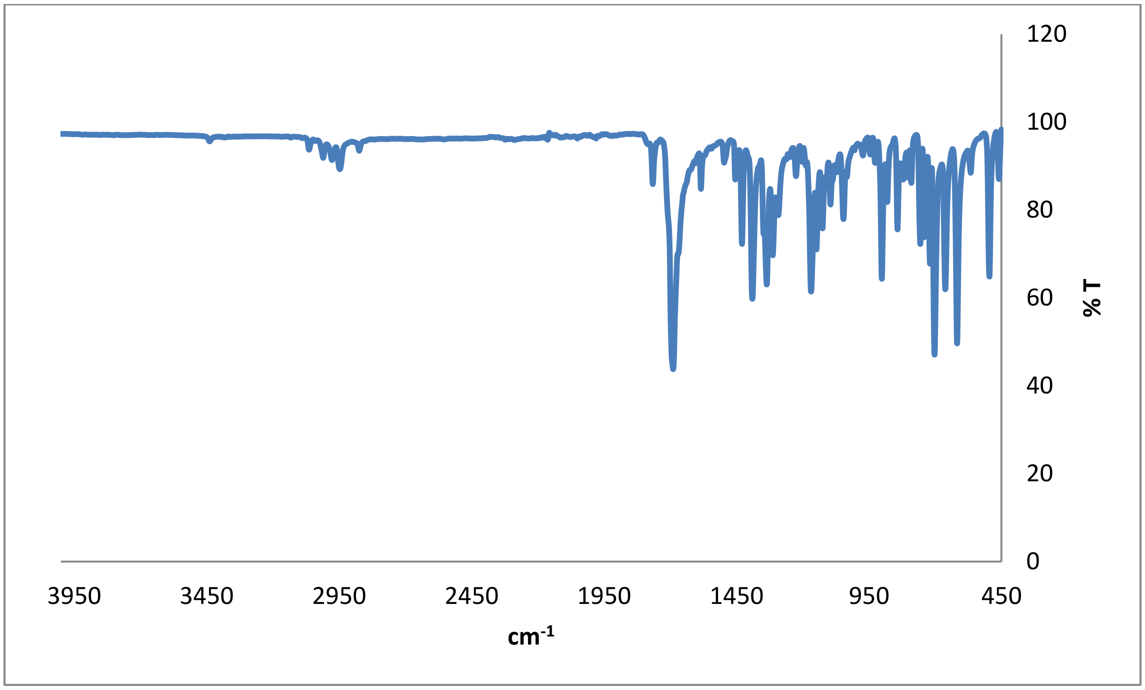

Figure S48. IR spectrum of **7**

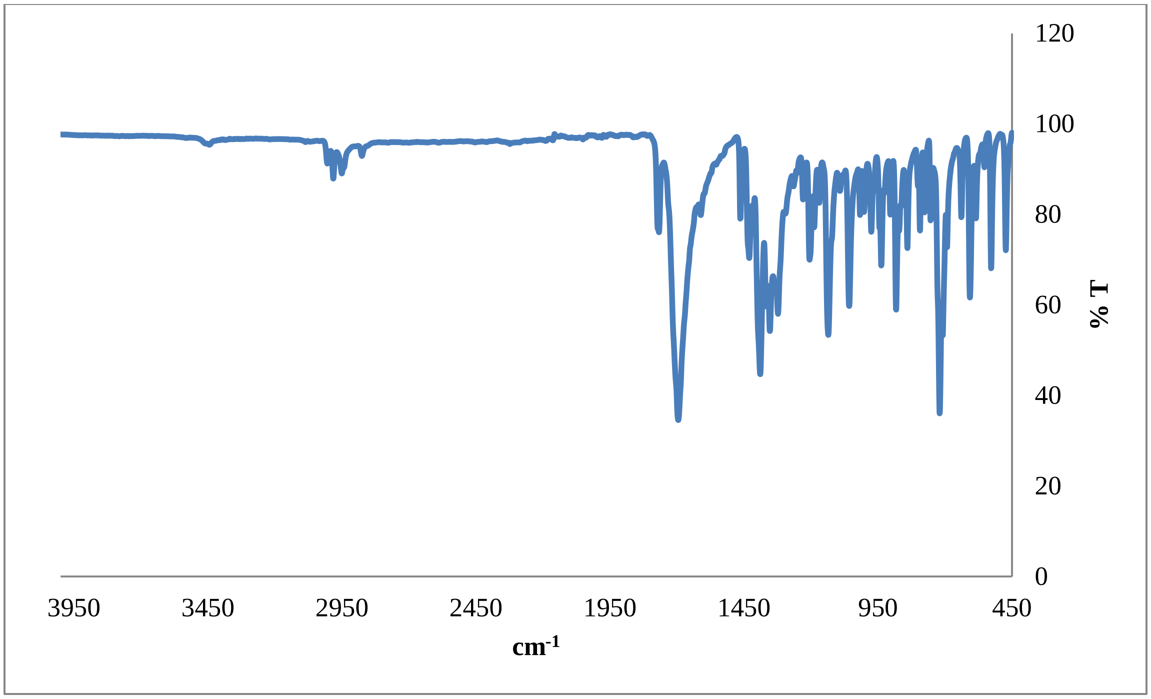

Figure S49. IR spectrum of **8**

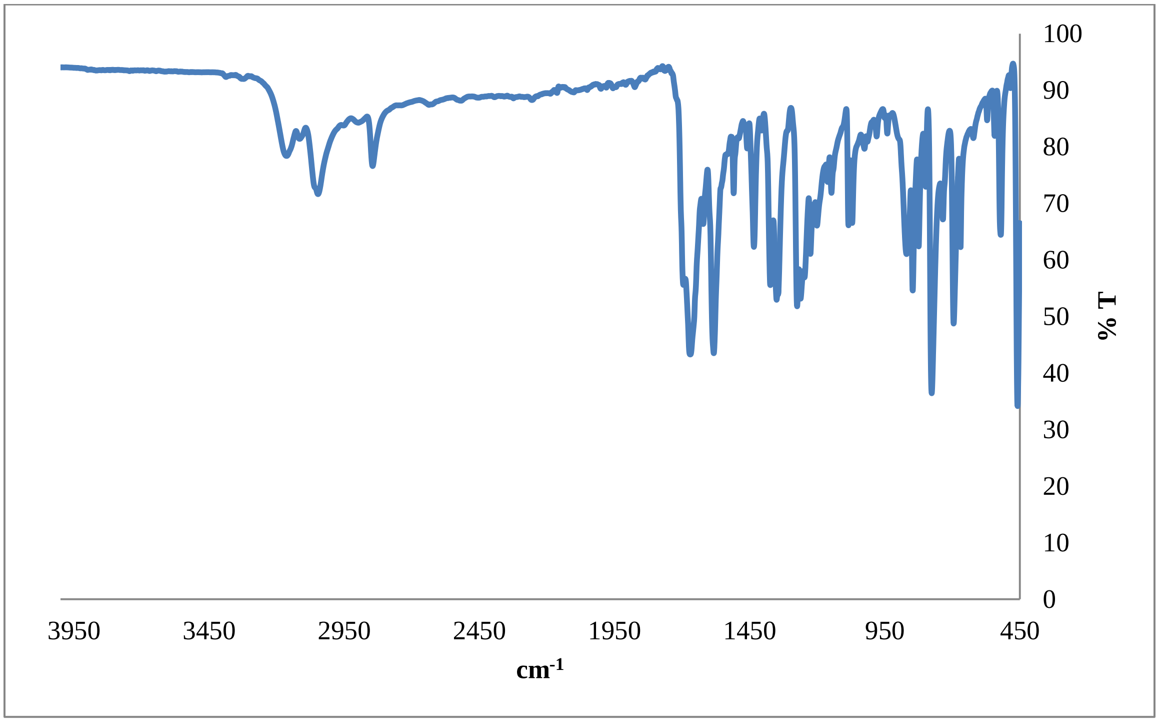

Figure S50. IR spectrum of **18**

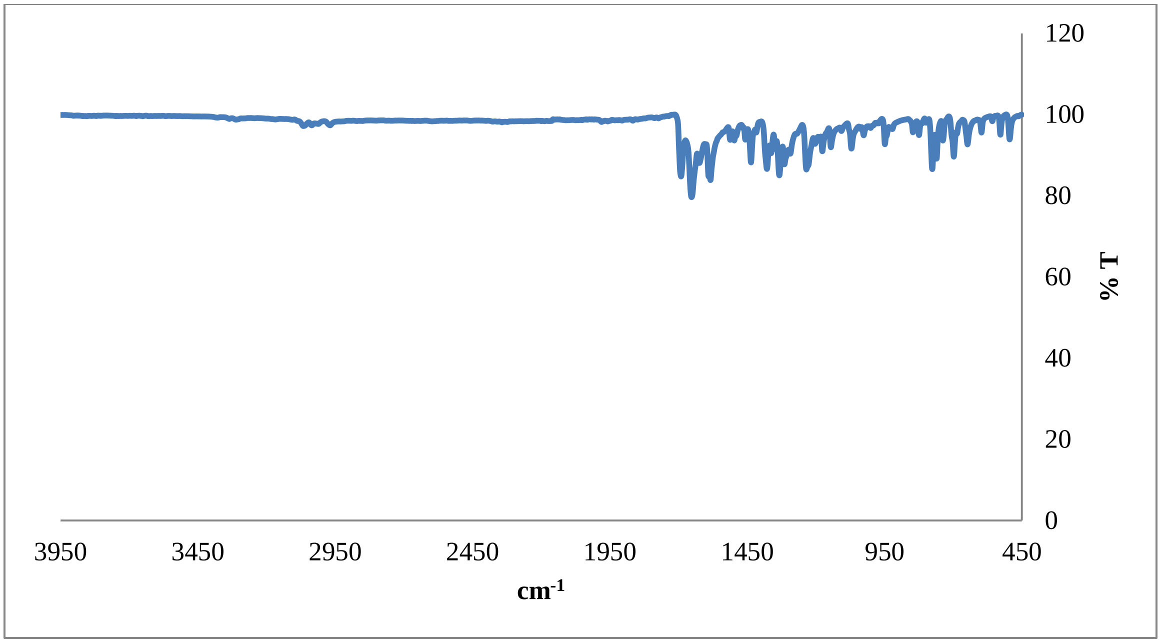

Figure S51. IR spectrum of **19**

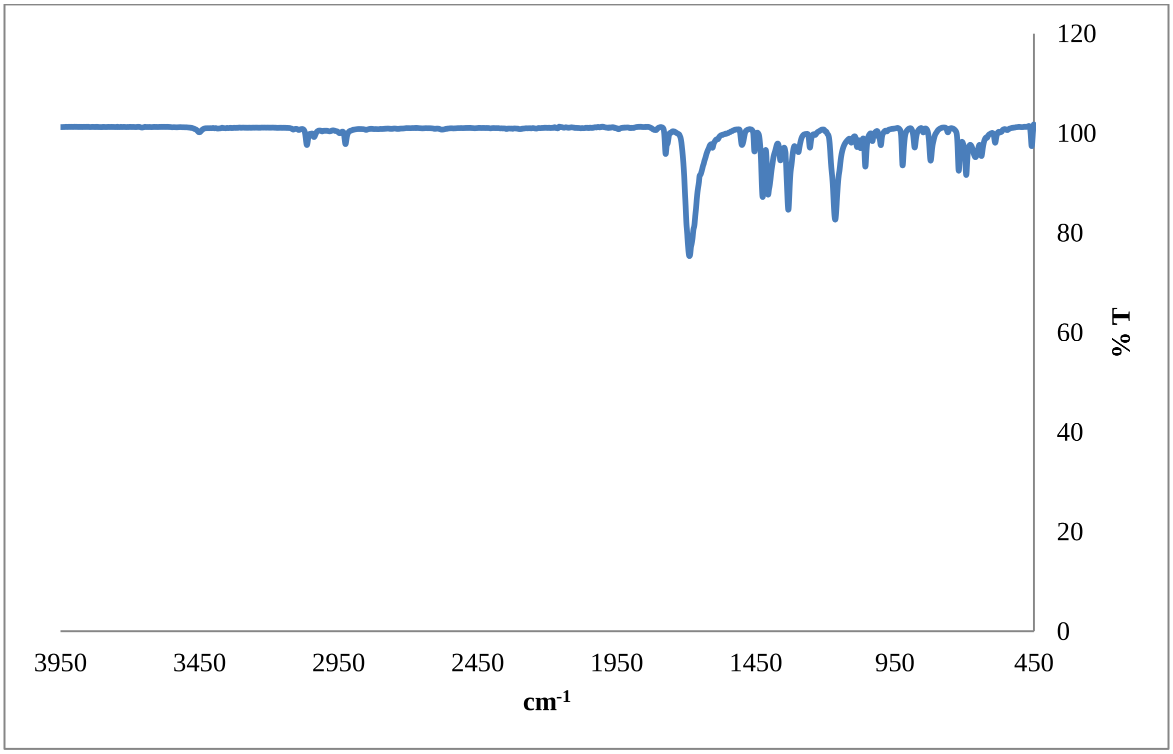

Figure S52. IR spectrum of **20**

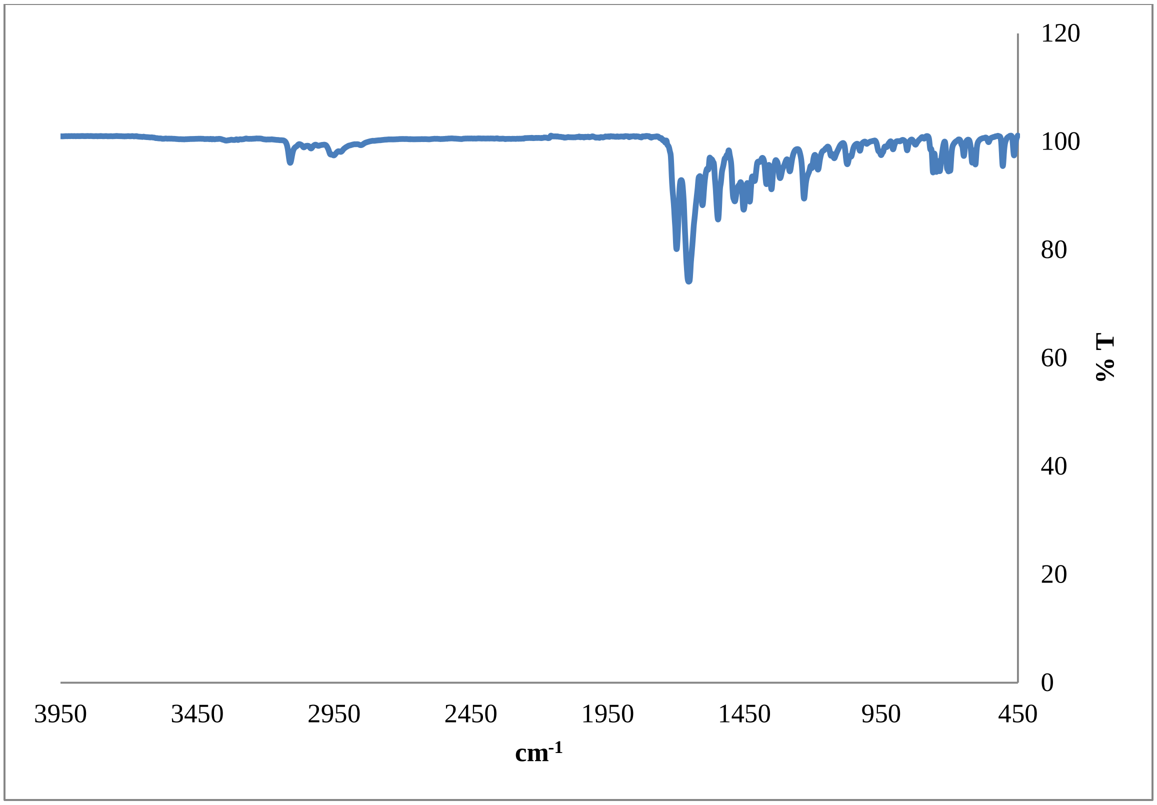

Figure S53. IR spectrum of **21**

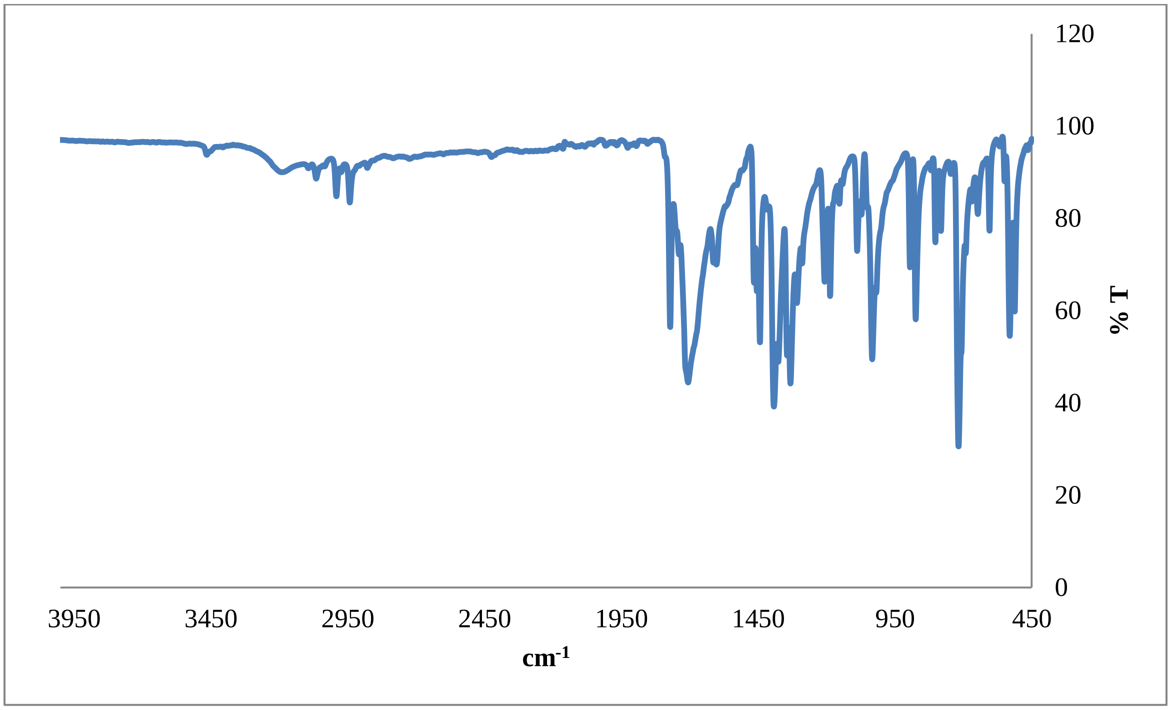

Figure S54. IR spectrum of **22**

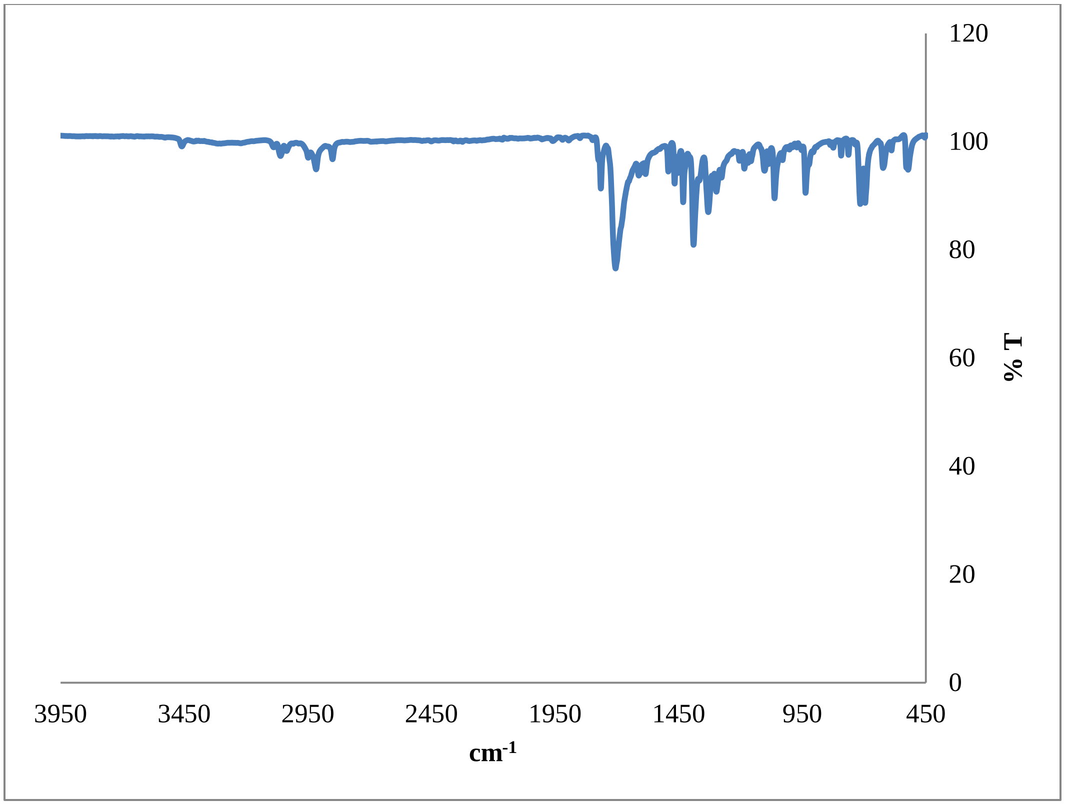

Figure S55. IR spectrum of **23**

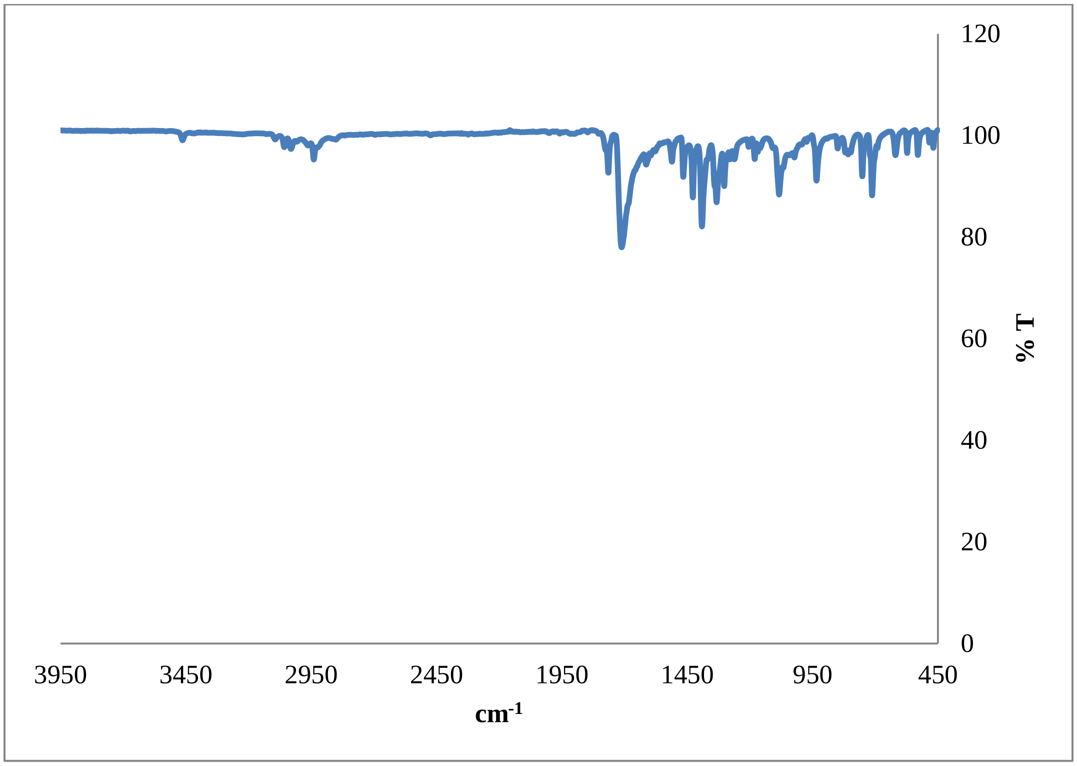

Figure S56. IR spectrum of **24**

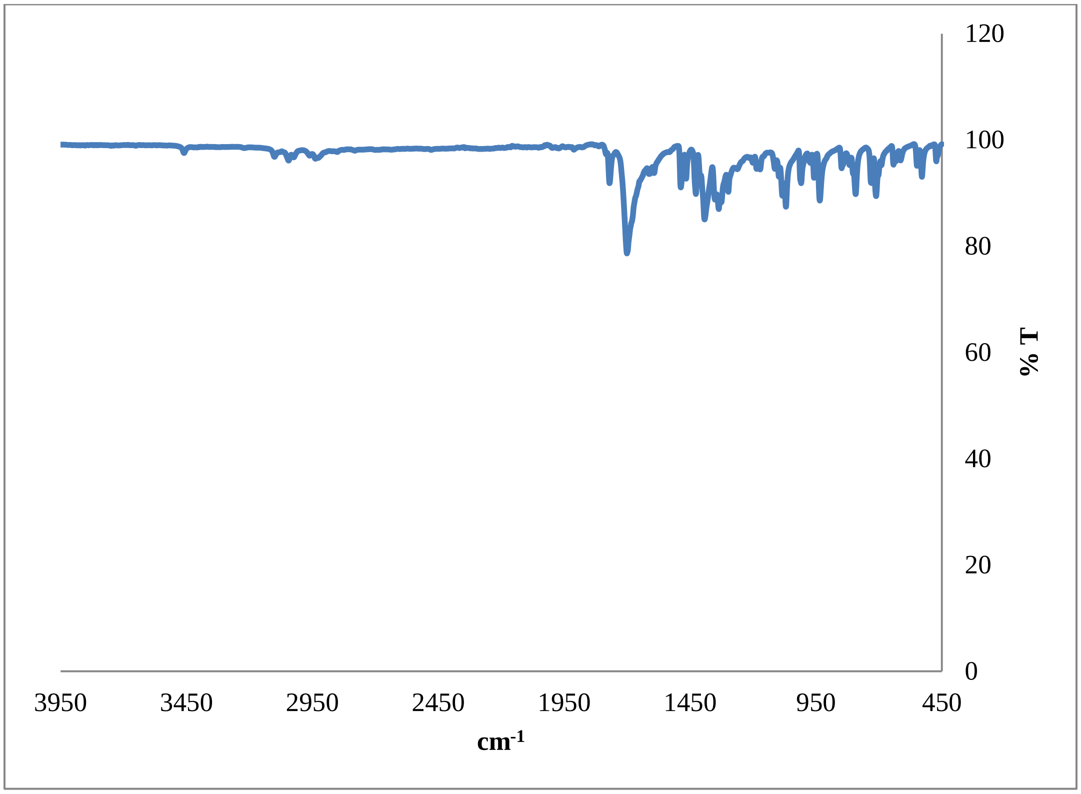

Figure S57. IR spectrum of **25**

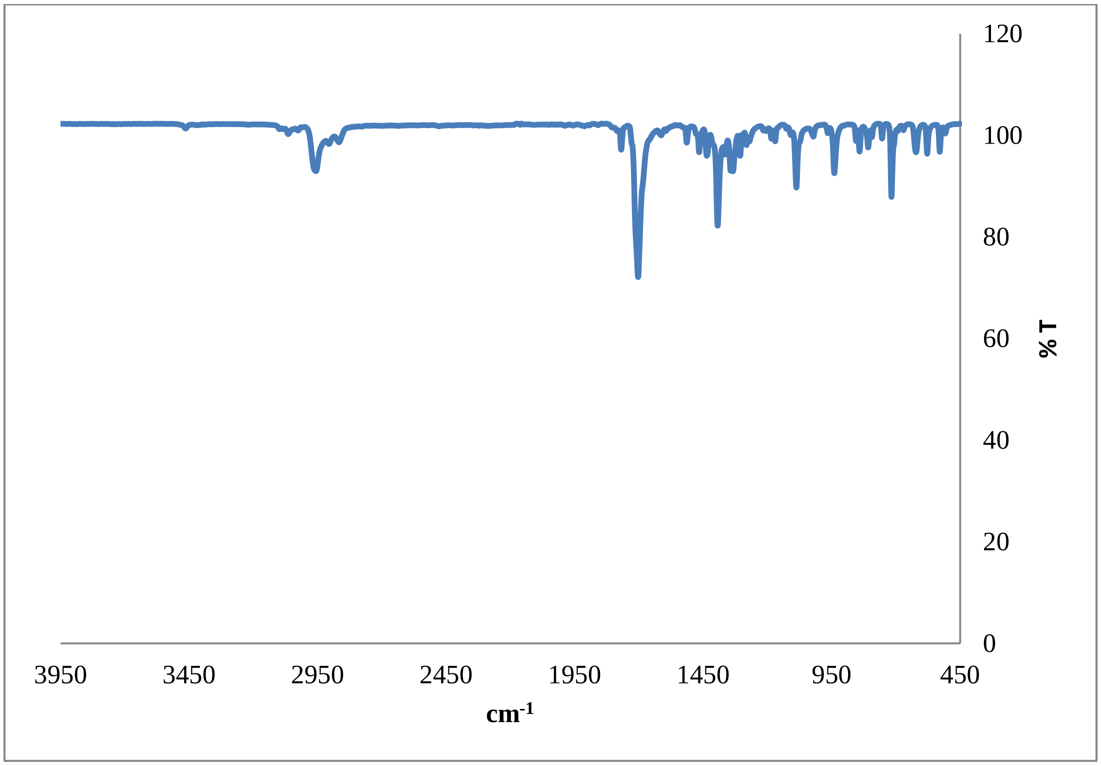

Figure S58. IR spectrum of **26**

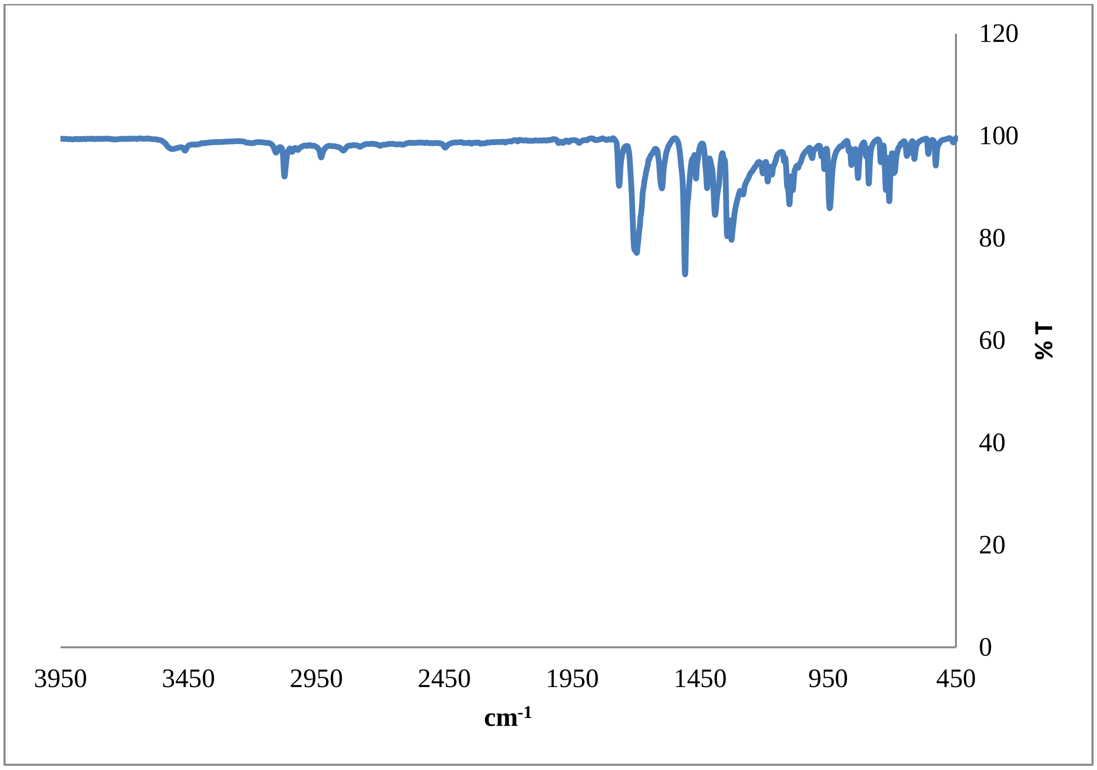

Figure S59. IR spectrum of **27**

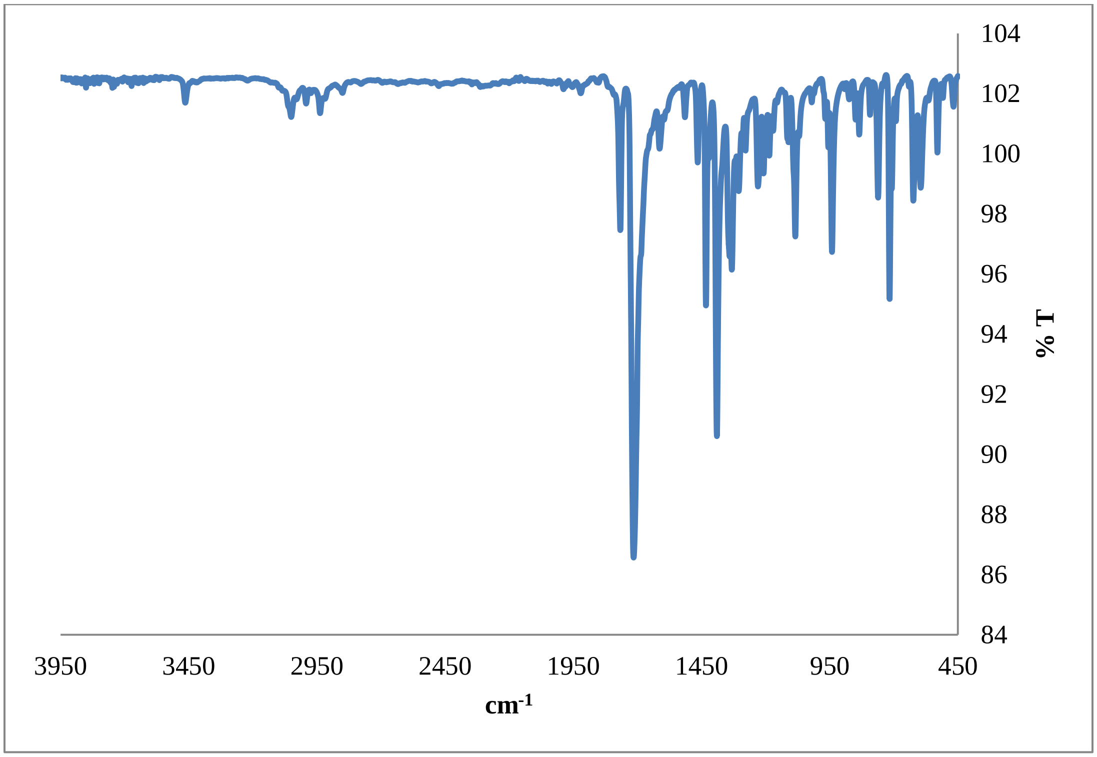

Figure S60. IR spectrum of **28**

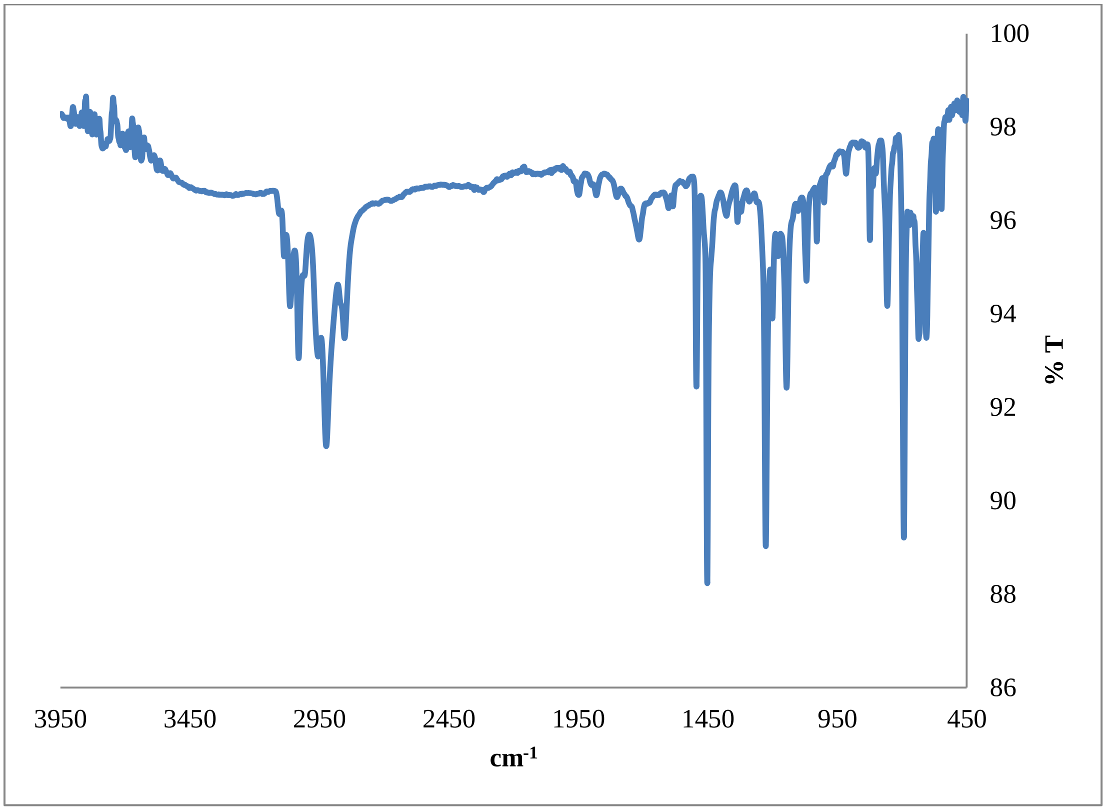

Figure S61. IR spectrum of **30**

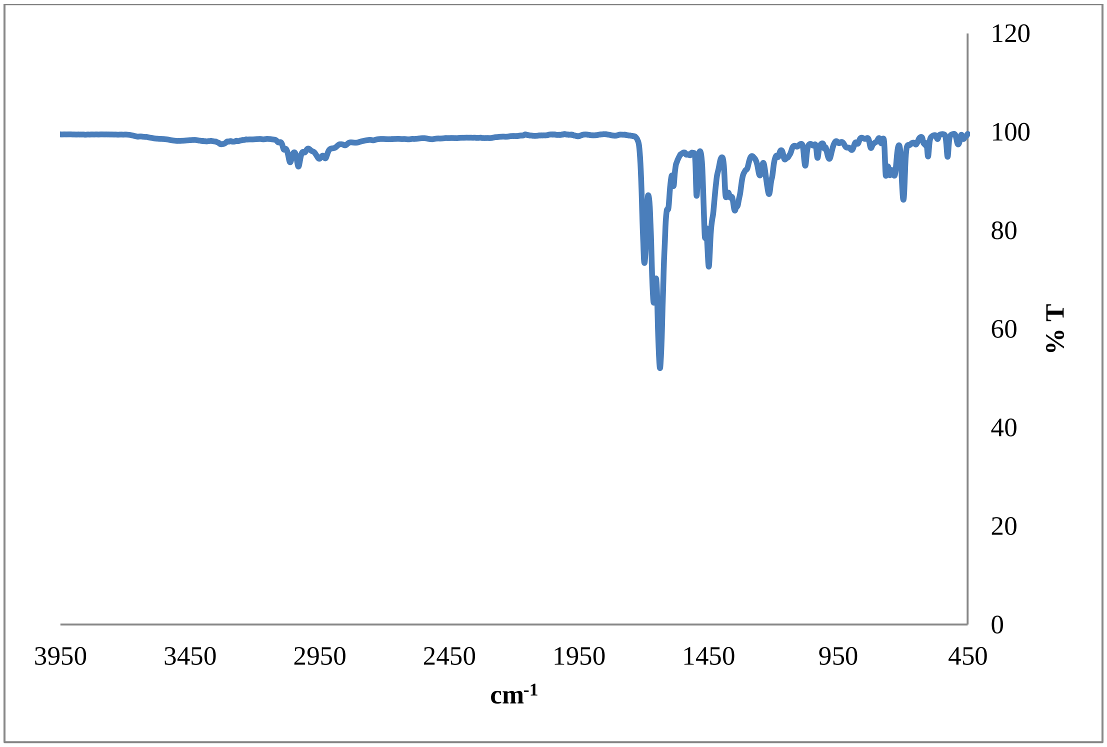

Figure S62. IR spectrum of **31**

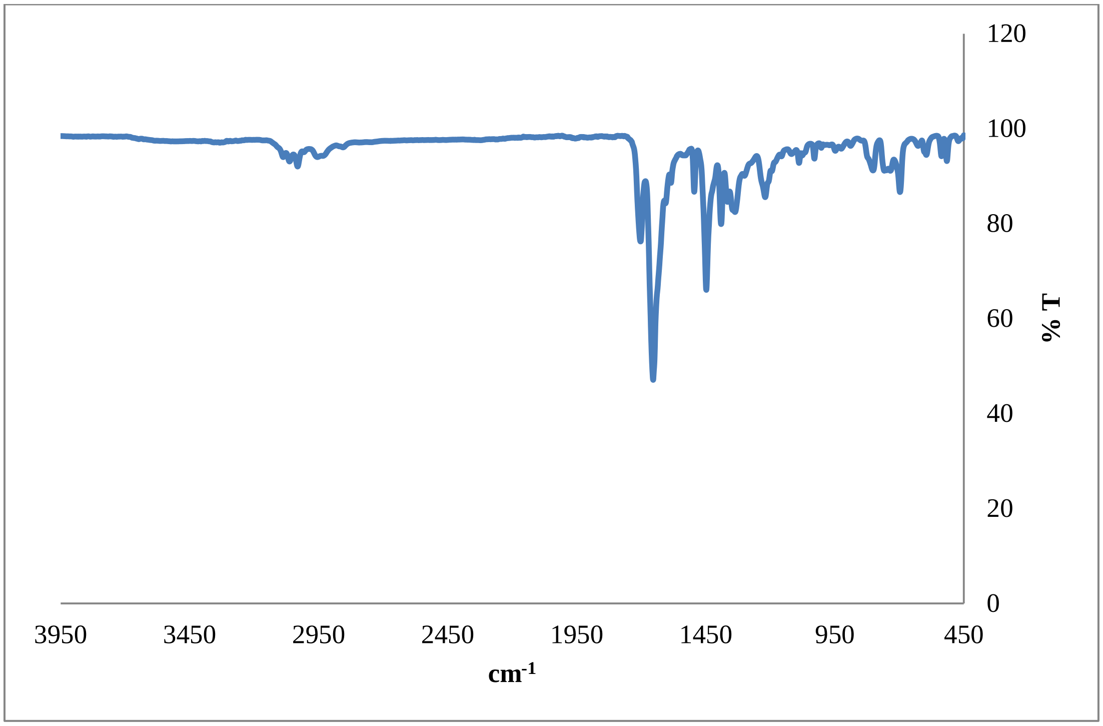

Figure S63. IR spectrum of **32**

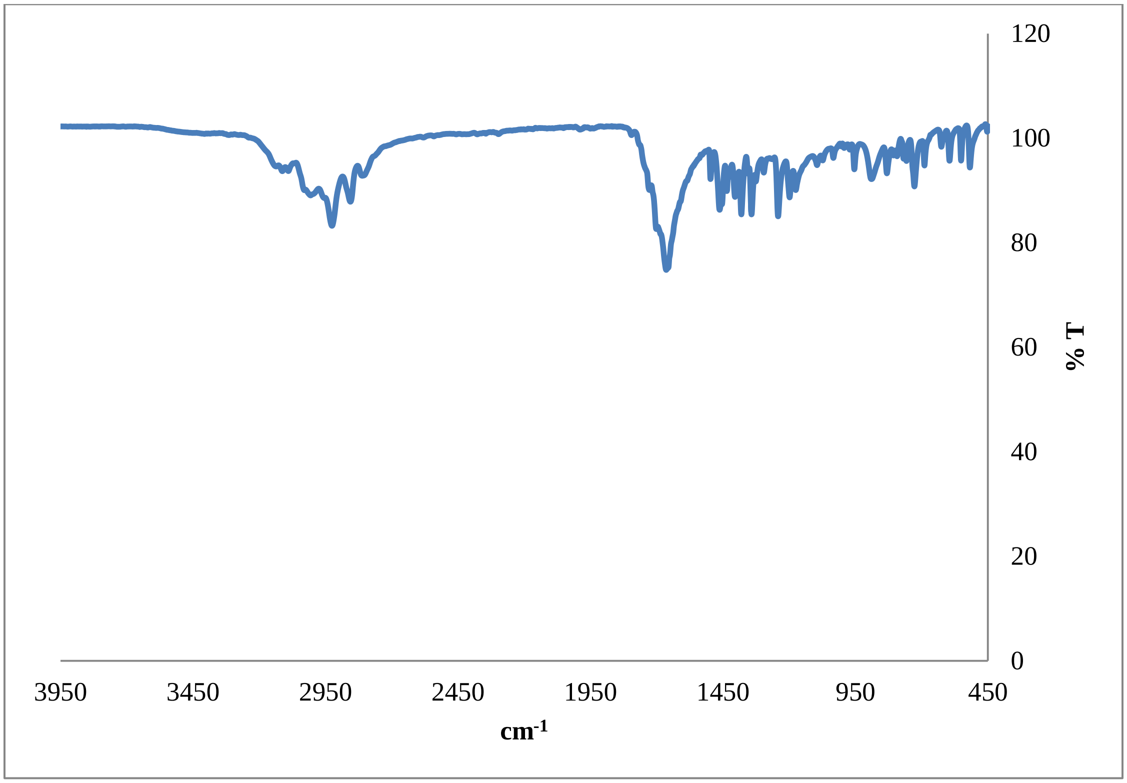

Figure S64. IR spectrum of **33**

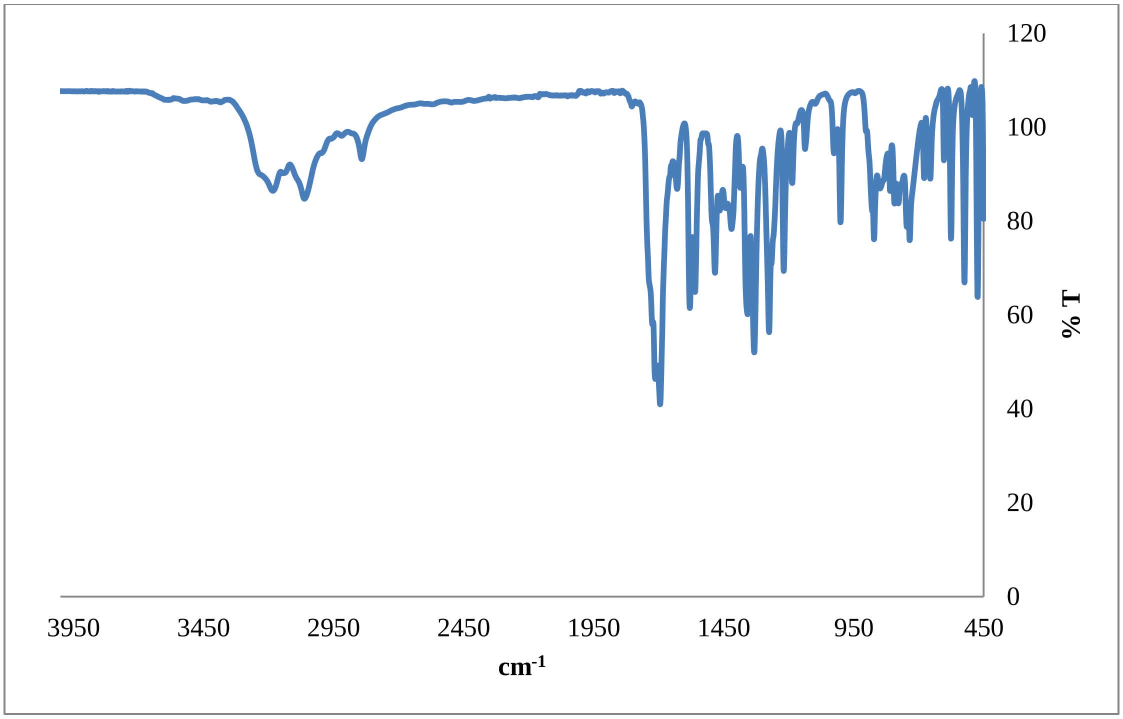

Figure S65. IR spectrum of **36**

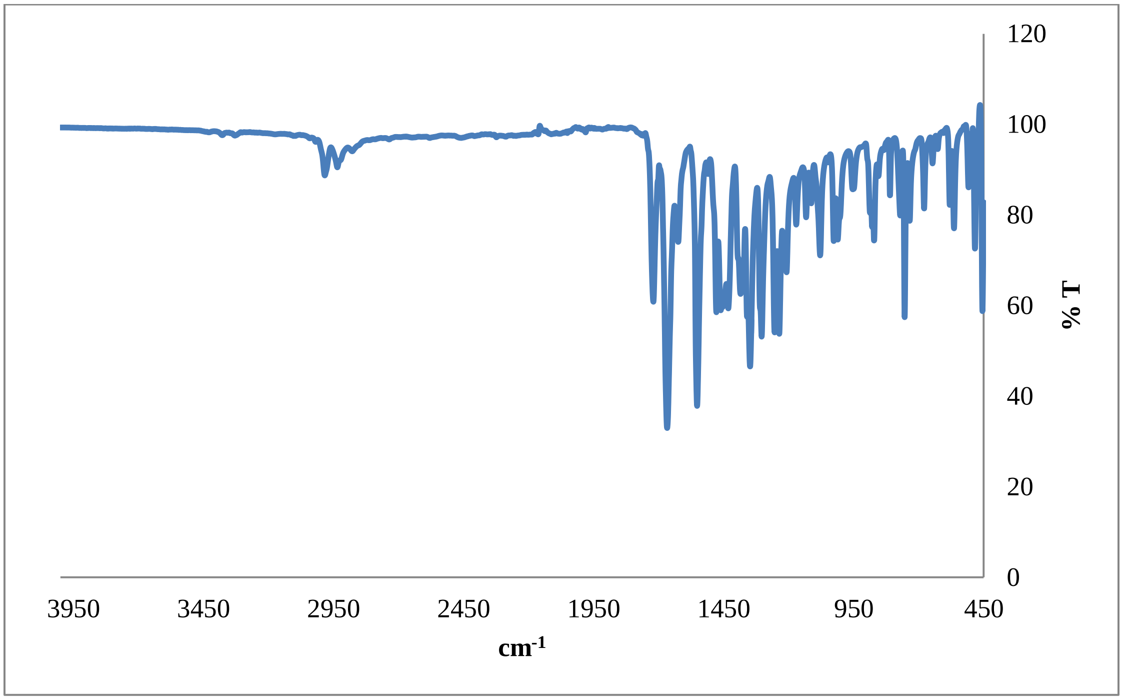

Figure S66. IR spectrum of **37**

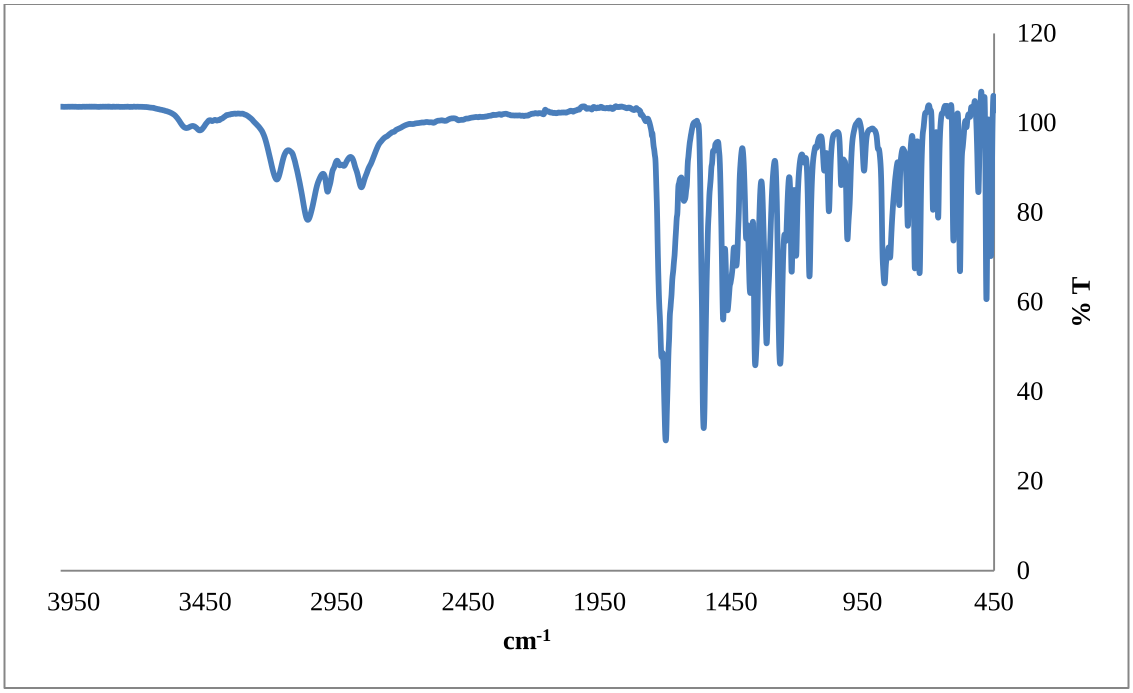

Figure S67. IR spectrum of **38**

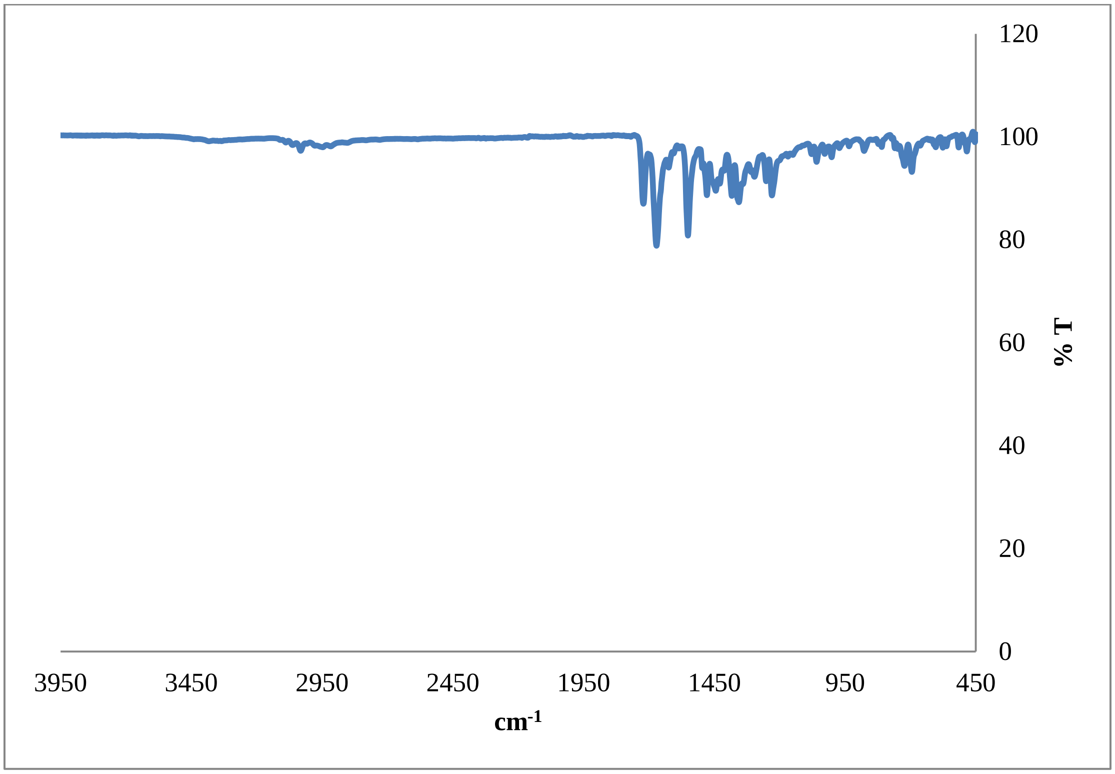

Figure S68. IR spectrum of **39**

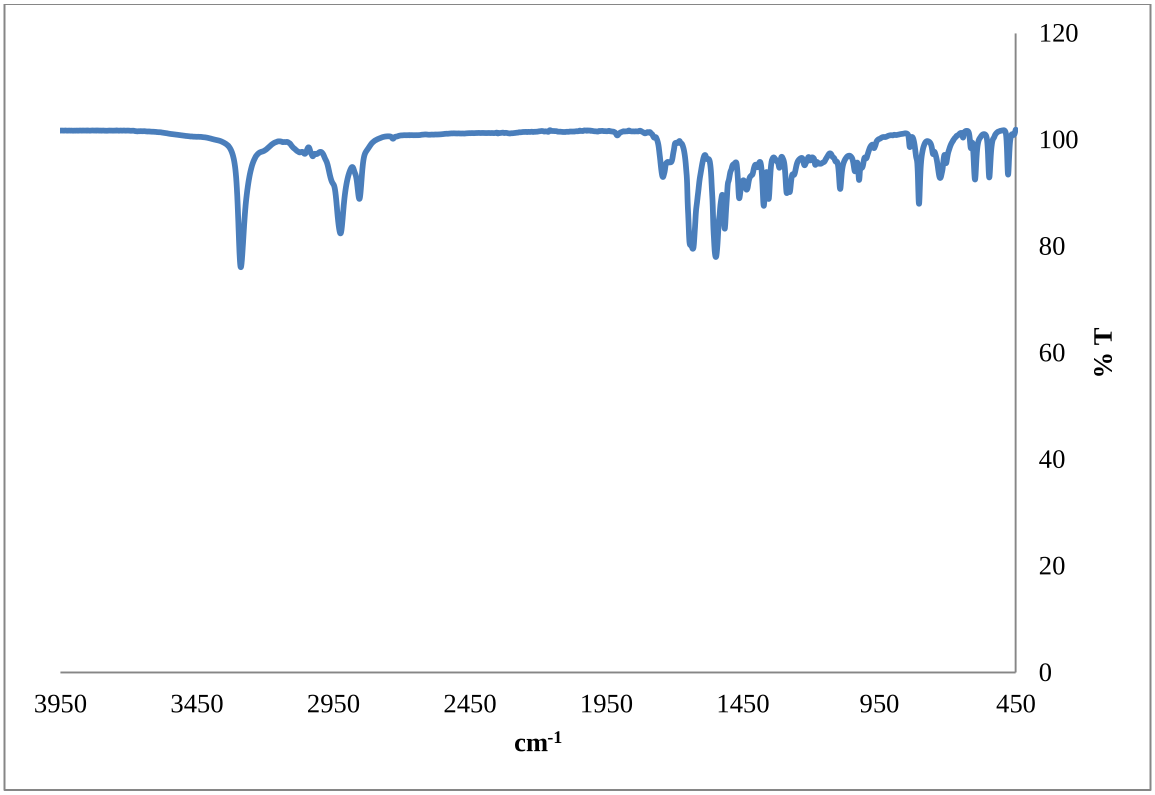

Figure S69. IR spectrum of **42**

Ex-situ following of deprotonation reaction

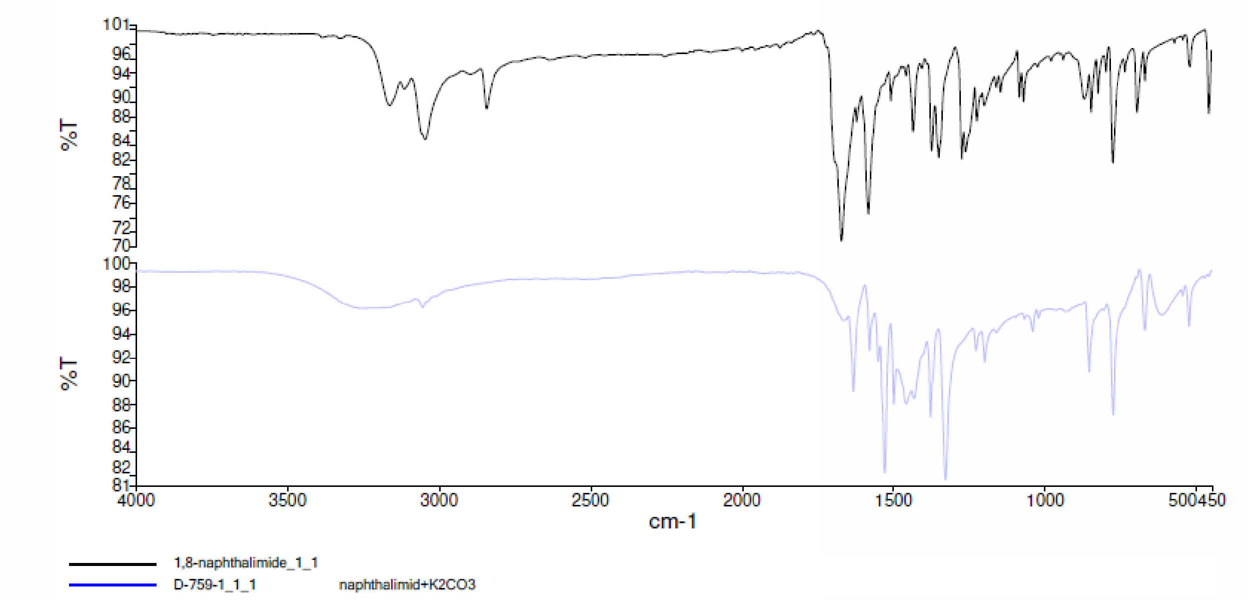

Figure S70. Ex situ IR spectroscopy of reaction of **11** and K_2CO_3: a) naphthalimide **11**; b) first step - phthalimide + K_2CO_3, 1 h milling

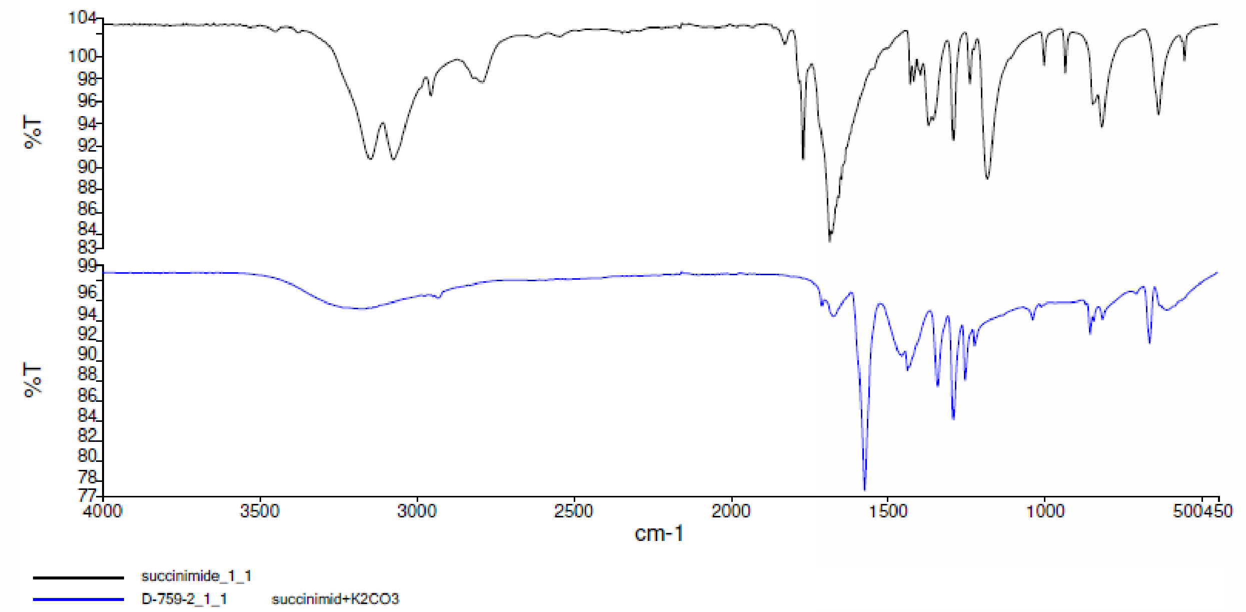

Figure S71. Ex situ IR spectroscopy of reaction of **13** and K_2CO_3: a) succinimide **13**; b) first step - phthalimide + K_2CO_3, 1 h milling

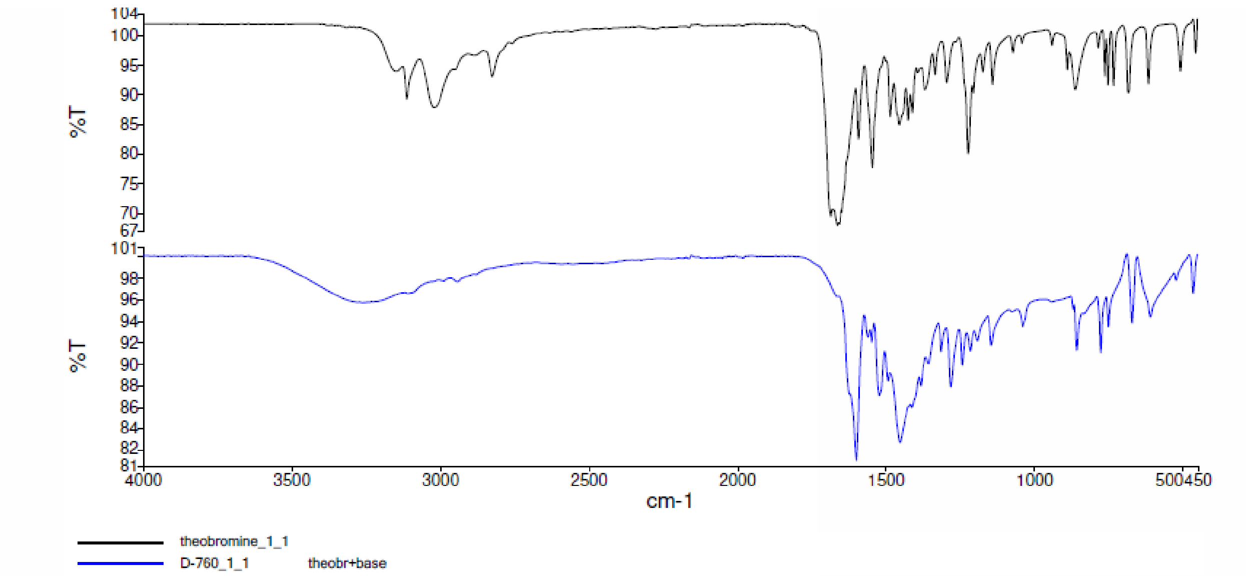

Figure S72. Ex situ IR spectroscopy of reaction of **14** and K_2CO_3: a) theobromine **14**; b) first step - phthalimide + K_2CO_3, 1 h milling

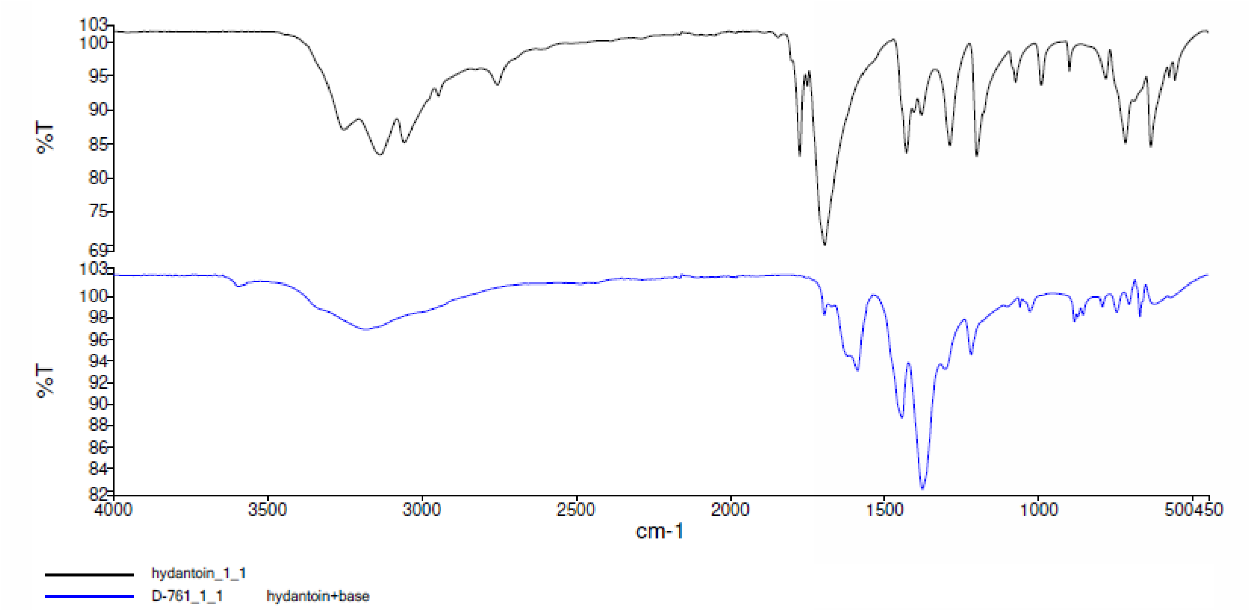

Figure S73. Ex situ IR spectroscopy of reaction of **15** and K_2CO_3: a) hydantoin **15**; b) first step - phthalimide + K_2CO_3, 1 h milling

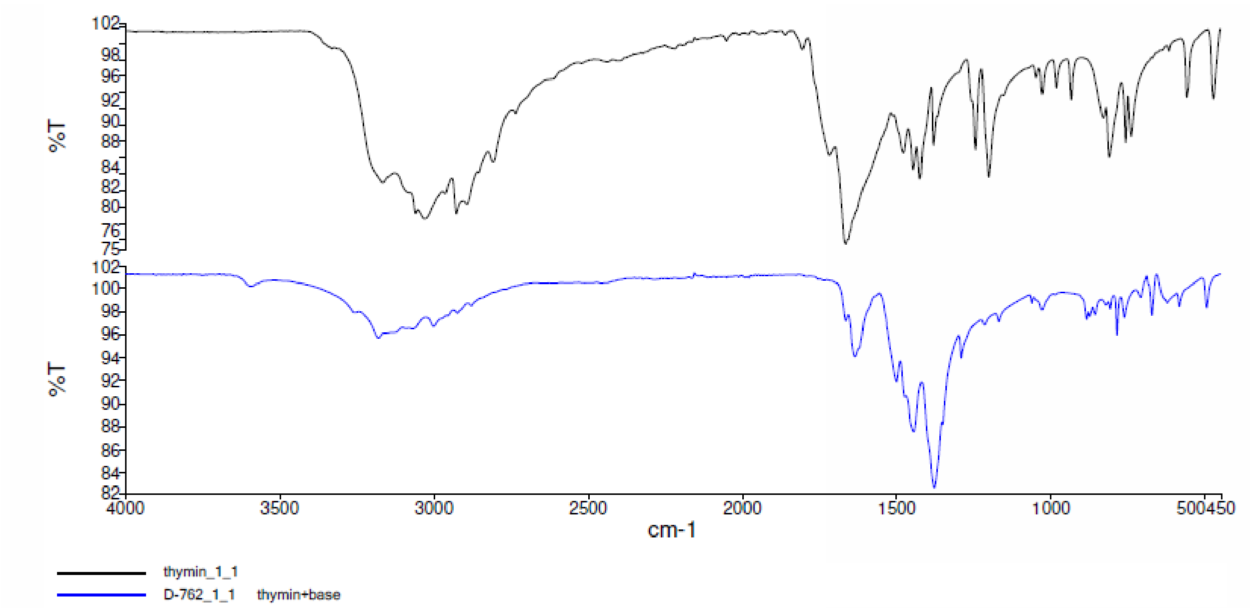

Figure S74. Ex situ IR spectroscopy of reaction of **16** and K_2CO_3: a) thymine **16**; b) first step - phthalimide + K_2CO_3, 1 h milling

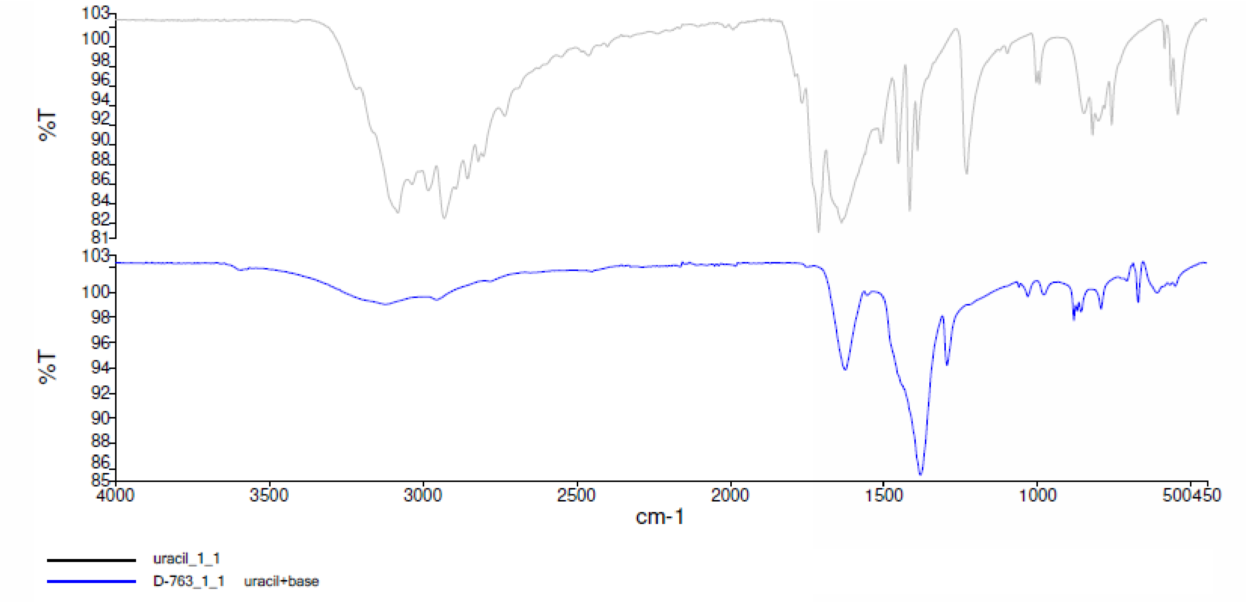

Figure S75. Ex situ IR spectroscopy of reaction of **17** and K_2CO_3: a) uracil **17**; b) first step - phthalimide + K_2CO_3, 1 h milling

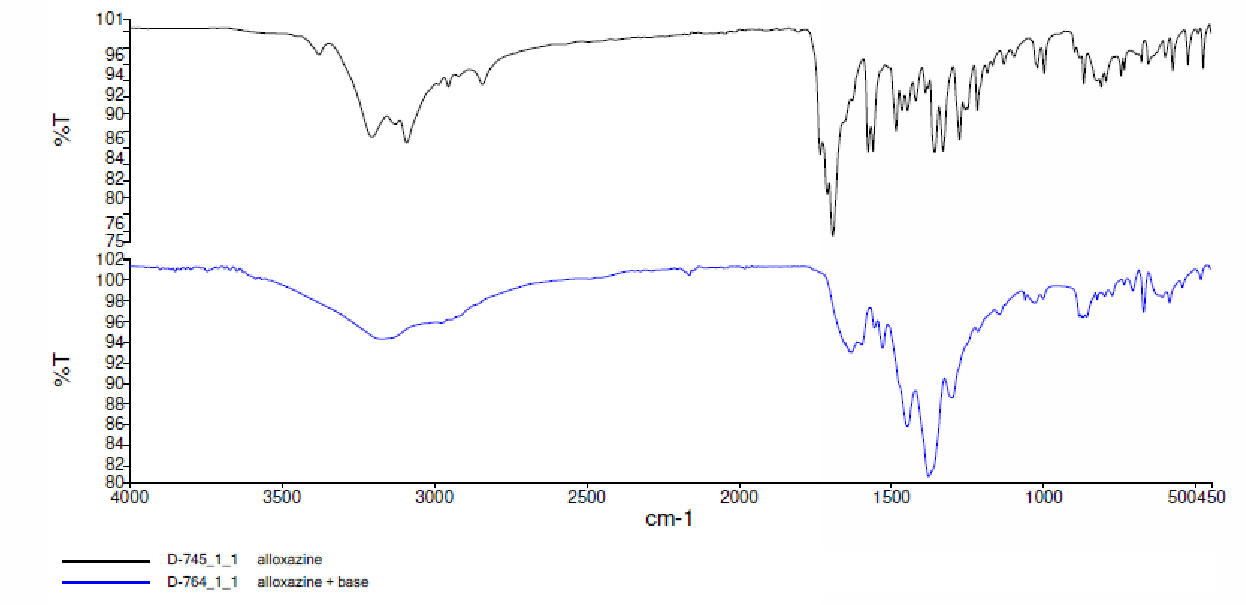

Figure S76. Ex situ IR spectroscopy of reaction of **36** and K_2CO_3: a) alloxazine **36**; b) first step - phthalimide + K_2CO_3, 1 h milling

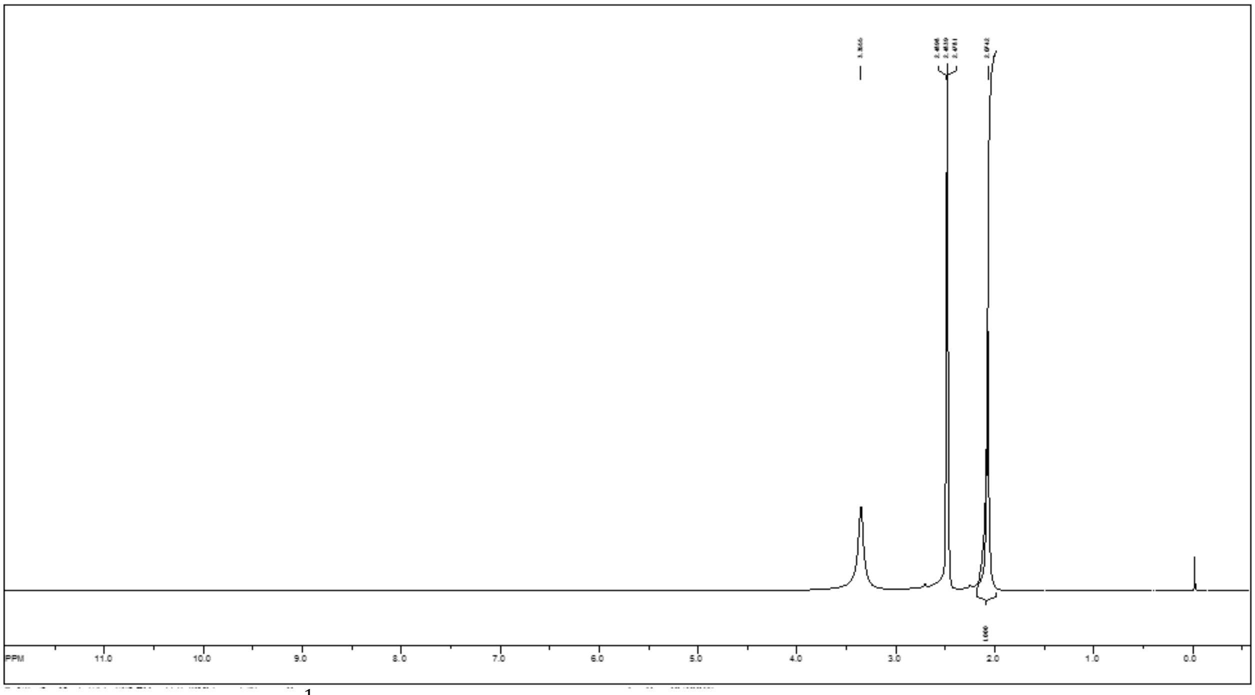

Figure S77. ^{1}H NMR spectrum of depotonated succinimide **13** in DMSO-d_6

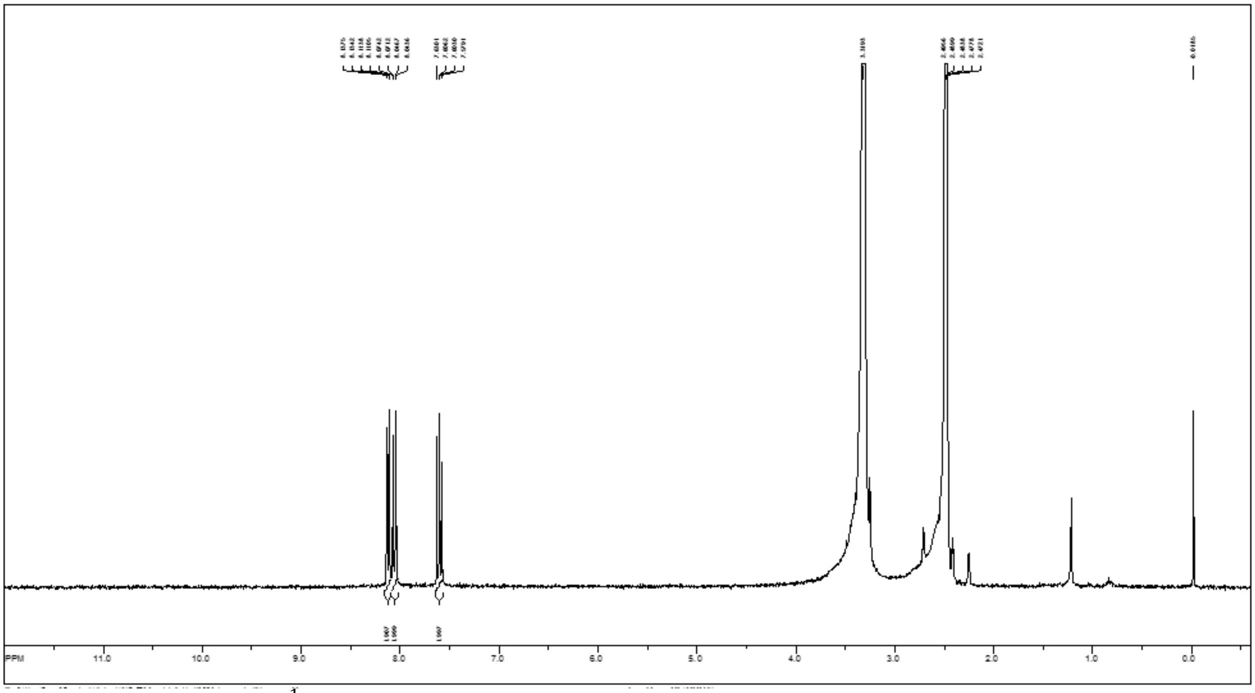

Figure S78. ^{1}H NMR spectrum of depotonated naphthalimide **11** in DMSO-d_6

Table S1. Physical state (PS) of reagents and products[a]

Substrate	PS	Alkyl halide	PS	Product	PS
**1**	S	**2**	L	**3**<br>**4**	S<br>S
		EtBr	L	**5**	S
		EtI	L	**5**	S
		BuCl	L	**6**	S
		BnBr	L	**7**	S
		N-(2-bromoethyl)phthalimide	S	**8**	S
		$ClCH_2CH_2Cl$	L	**9, 10**	
**11**	S	EtBr	L	**18**	S
		BnBr	L	**19**	S
**12**	S	EtBr	L	**22**	S
		BnBr	L	**23**	S
		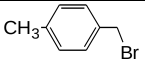	S	**24**	S
		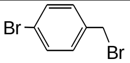	S	**25**	S
		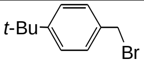	L	**26**	S
		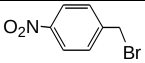	S	**27**	S
		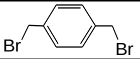	S	**28**	S
**13**	S	BnBr	L	**20**	S
**14**	S	BnBr	L	**21**	L
**15**	S	BnBr	L	**30**	L
**16**	S	BnBr	L	**31**	L
**17**	S	BnBr	L	**32**<br>**33**	L<br>S
**36**	S	EtBr	L	**37**<br>**38**	S<br>S
		BnBr	L	**39**	S

[a] L = liquid, S = solid

Computational details

Gaussian03 program package [26] implemented on dual core Opteron 240 personal computer under Linux operating system was used for all calculations. All structures were optimized at the B3LYP/6-31G* level and energies computed by B3LYP/6-311+G**//B3LYP/6-31G*+ZPVE method. Activation energies were estimated from the transition state calculations and all transition state structures were confirmed by vibrational analysis and the possesion of single negative mode of vibration.

References

1. Armarego, W. L. F.; Perrin, D. D. *Purification of laboratory chemicals*; 4th ed., Butterworth-Heinemann: Oxford, 1996.
2. Morgan, M. S.; Tipson, R. S.; Lowy, A.; Baldwin, W. E. *J. Am. Chem. Soc.* **1944**, *66*, 404-407. doi: 10.1021/ja01231a028
3. Briš, A.; Trošelj, P.; Margetić, D.; Flamigni, L.; Ventura, B. *ChemPlusChem* **2016**, *81*, 985-994. doi: 10.1002/cplu.201600231
4. Long, B. M.; Pfeffer, F. M. *Chem. Asian J.* **2014**, *9*, 1091-1098. doi: 10.1002/asia.201301677
5. Rush, A. M.; Nelles, D. A.; Blum, A. P.; Barnhill, S. A.; Tatro, E. T.; Yeo, G. W.; Gianneschi, N. C. *J. Am. Chem. Soc*. **2014**, *136*, 7615-7618. doi: 10.1021/ja503598z
6. Trošelj, P.; Đilović, I.; Matković-Čalogović, D.; Margetić, D. *J. Heterocycl. Chem.* **2013**, *50*, 83-90. doi: 10.1002/jhet.998
7. Tian, Z.; Xie, S.; Du, Y.; Ma, Y.; Zhao, J.; Gao, W.; Wang, C. *Eur. J. Med. Chem.* **2009**, *44*, 393-399. doi: 10.1016/j.ejmech.2008.02.044
8. Barooah, N.; Tamuly, C.; Baruah, J. B. *J. Chem. Sci.* **2005**, *117*, 117-122. doi: 10.1007/BF03356105
9. Zeng, H.-T.; Huang, J.-M. *Org. Lett.* **2015**, *17*, 4276-4279. doi: 10.1021/acs.orglett.5b02063
10. Bram, G.; Decodts, G.; Bensaid, Y.; Farnoux, C. C.; Galons, H.; Miocque, M. *Synthesis* **1985**, *5*, 543-545. doi: 10.1055/s-1985-31269
11. Le, Z.-G.; Chen, Z.-C.; Hu, Y.; Zheng, Q.-G. *Synthesis* **2004**, *2*, 208-212. doi: 10.1055/s-2003-44383
12. Cao, H.; Alper, H. *Org. Lett.* **2010,** *12*, 4126-4129. doi: 10.1021/ol101714p
13. Manske, R. H. F. *Org. Synth. Col.* Vol. 2, **1943**, p. 83, Vol. 12, **1932**, p. 10.
14. Hörtner, S. R.; Ritschel, T.; Stengl, B.; Kramer. C.; Schweizer, W. B.; Wagner, B.; Kansy, M.; Klebe, G.; Diederich, F. *Angew. Chem. Int. Ed. Engl.* **2007**, 46, 8266-8269. doi: 10.1002anie.200702961
15. Yedage, S. L.; Denvert S.; D'silva, D. S.; Bhanage, B. M. *RSC Adv.* **2015**, *5*, 80441-80449. doi: 10.1039/C5RA13094H
16. Baumgartner, C.; Eberle, C.; Diederich, F.; Lauw, S.; Rohdich, F.; Eisenreich, W.; Bacher, A. *Helv. Chim. Acta* **2007**, *90*, 1043-1067. doi: 10.1002/hlca.200790105
17. Karginov, V. A.; Nestorovich, E. A.; Yohannes, A.; Robinson, T. M.; Fahmi, N. E.; Schmidtmann, F.; Hecht, S. M.; Bezrukov, S. M. *Antimicrob. Agents Chemother.* **2006**, *50*, 3740-3753. doi: 10.1128/AAC.00693-06
18. Kalidas, P.; DeWitt, Blanton, C., Jr. *J. Pharm. Sci.* **1976**, *65*, 1527-1530. doi: 10.1002/jps.2600651028
19. Ulgheri, F.; Giunta, D.; Spanu, P. *Tetrahedron* **2008**, *64*, 11768-11775. doi: 10.1016/j.tet.2008.09.080
20. Ishikawa, I.; Itoh, T.; Takayanagi, H.; Oshima, J., Kawahara, N.; Mizuno, Y.; Ogura, H. *Chem. Pharm. Bull.* **1991**, *39*, 1922-1930. doi: 10.1248/cpb.39.1922
21. Botta, M.; Summa, V.; Saladino, R.; Nicoletti, R. *Synth. Commun.* **1991**, *21*, 2181-2187. doi: 10.1080/00397919108055451
22. Gonzalo, G.; Smit, C.; Jin, J.; Minnaard, A. J.; Fraaije, M. W. *Chem. Commun.* **2011**, *47*, 11050-11052. doi: 10.1039/c1cc14039f
23. Robak, A. J.; Branchaud, B. P. *Tetrahedron Lett.* **2005**, *46*, 5651-5654. doi: 10.1016/j.tetlet.2005.06.102

24. Firouzabadi, F.; Sardarian, A. R.; Badparva, H. *Synth. Commun.* **1994**, *24*, 601-607. doi: 10.1080/00397919408012637
25. Kanie, O.; Crawley, S. C.; Palcic, M. M.; Hindsgaul, O. *Carbohydr. Res.* **1993**, *243*, 139-164. doi: 10.1016/0008-6215(93)84087-M
26. *Gaussian 03*, Revision B.03, M. J. Frisch, G. W. Trucks, H. B. Schlegel, G. E. Scuseria, M. A. Robb, J. R. Cheeseman, J. A. Montgomery, Jr., T. Vreven, K. N. Kudin, J. C. Burant, J. M. Millam, S. S. Iyengar, J. Tomasi, V. Barone, B. Mennucci, M. Cossi, G. Scalmani, N. Rega, G. A. Petersson, H. Nakatsuji, M. Hada, M. Ehara, K. Toyota, R. Fukuda, J. Hasegawa, M. Ishida, T. Nakajima, Y. Honda, O. Kitao, H. Nakai, M. Klene, X. Li, J. E. Knox, H. P. Hratchian, J. B. Cross, C. Adamo, J. Jaramillo, R. Gomperts, R. E. Stratmann, O. Yazyev, A. J. Austin, R. Cammi, C. Pomelli, J. W. Ochterski, P. Y. Ayala, K. Morokuma, G. A. Voth, P. Salvador, J. J. Dannenberg, V. G. Zakrzewski, S. Dapprich, A. D. Daniels, M. C. Strain, O. Farkas, D. K. Malick, A. D. Rabuck, K. Raghavachari, J. B. Foresman, J. V. Ortiz, Q. Cui, A. G. Baboul, S. Clifford, J. Cioslowski, B. B. Stefanov, G. Liu, A. Liashenko, P. Piskorz, I. Komaromi, R. L. Martin, D. J. Fox, T. Keith, M. A. Al-Laham, C. Y. Peng, A. Nanayakkara, M. Challacombe, P. M. W. Gill, B. Johnson, W. Chen, M. W. Wong, C. Gonzalez, J. A. Pople, Gaussian Inc., Wallingford CT, **2004**.
